# Synthesis, characterization and antidiabetic studies of novel amantadine-derived Schiff base (AHB) and its Zn(ii), Co(ii), Cr(iii) and VO(iv) complexes†

**DOI:** 10.1039/d5ra00065c

**Published:** 2025-06-04

**Authors:** Aliya Ajaz, Muhammad Ashraf Shaheen, Muhammad Fayyaz ur Rehman, Maqsood Ahmad, Khurram Shahzad Munawar, Abu Bakar Siddique, Muhammad Ashfaq, Nazir Ahmad

**Affiliations:** a Institute of Chemistry, University of Sargodha 40100 Pakistan ashraf.shaheen@uos.edu.pk; b Department of Allied Health Sciences, Superior University Sargodha-Campus 40100 Pakistan; c Institute of Chemistry, The Islamia University of Bahawalpur Baghdad-ul-Jadeed Campus 63100 Pakistan; d Department of Chemistry, University of Mianwali Mianwali 42200 Pakistan; e Department of Physics, University of Sargodha 42200 Pakistan; f Department of Chemistry, Government College University Lahore 54000 Pakistan

## Abstract

Diabetes mellitus is a significant health issue in Pakistan, with rising prevalence rates posing serious challenges to public health. To tackle this issue, several strategies have been developed recently, among which the controlled conversion of dietary carbohydrates into glucose by inhibition of enzymes is an appropriate strategy. For this purpose, many synthetic drugs have been tried and explored. In this scenario, a new Schiff base ligand, (*E*)-2-((adamantan-2-ylimino)methyl)-6-bromo-4-chlorophenol (AHB), has been synthesized by a reflux reaction of amantadine with 3-bromo-5-chloro-2-hydroxybenzaldehyde. The transition metal complexes, [Cr(AHB)_3_], [Zn(AHB)_2_], [Co(AHB)_2_], and [VO(AHB)_2_], were also synthesized by reacting AHB with the respective metal salts. Structural elucidation of the prepared compounds was performed comprehensively by various physicochemical methods like UV-visible, ^1^H and ^13^C-NMR, FT-IR, mass spectrometry, elemental analysis, thermal analysis, and molar conductivity. Structural investigation of crystalline AHB was also performed by single-crystal X-ray crystallography. Based on the structural analysis, an octahedral geometry for the Cr(iii) complex, square planar geometry for the Co(ii) complex, tetrahedral geometry for the Zn(ii) complex, and square pyramidal geometry for the VO(iv) complex were proposed. The synthesized bidentate ligand was found to be coordinated with the metal ions *via* nitrogen and oxygen donor atoms. All of the synthesized compounds were found to be non-electrolytic. The thermogravimetric analysis demonstrated the thermally stable nature of complexes, more than the ligand. The hypoglycemic potential of the compounds was estimated by *in vitro* inhibitory activities of α-amylase and α-glucosidase enzymes. The tested ligand-AHB was found to be relatively inactive toward both enzymes, while its metal complexes were found to be good α-glucosidase inhibitors as compared to α-amylase inhibitors. Among all prepared compounds, the Zn(ii) complex was found to be a good α-glucosidase inhibitor comparable to the standard (acarbose), while other complexes showed a moderate inhibition of α-glucosidase. Both Cr(iii) and Co(ii) complexes were found to be effective α-amylase inhibitors. Based on the current findings, it can be anticipated that the synthesized compounds can be tried as effective therapeutic agents to cure diabetes mellitus.

## Abstract 2

Molecular docking and enzyme inhibition (α-amylase and α-glucosidase) activities of amantadine Schiff base (AHB) and its metal complexes to cure DM type II.

## Graphical abstract

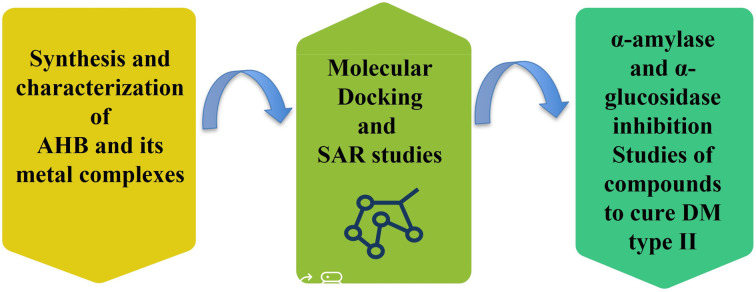

## 1 Introduction

Diabetes mellitus (DM) is a chronic metabolic condition marked by elevated blood glucose levels due to the body's ineffective utilization of insulin, preferably termed insulin resistance, and, over time, a gradual decline in the pancreatic beta cells' capacity to produce sufficient insulin.^1–3^ It may be recognized by fatigue, blurred vision, frequent urination, and gradual weight loss, and may result in serious complications, including cardiovascular diseases, neurological disorders, retinopathy, neuropathy, *etc.*^4–6^ In contrast to diabetes mellitus type 1, characterized by the immune system's destruction of insulin-producing cells, diabetes mellitus type 2 is predominantly a lifestyle-related condition influenced by factors including inadequate diet, insufficient physical activity, obesity, stress, and genetic susceptibility.^7–9^

Previously, several strategies have been opted to control and cure DM, like lowering glucose production in the liver by herbal extracts and allopathy drugs, stimulating the pancreas beta cells to produce more insulin, inhibition of ferroptosis by quercetin, injecting incretin-like hormones, *etc.*^10–12^ Among several therapies, inhibiting enzymes, like α-amylase and α-glucosidase, that are actively involved in glucose production by breaking down starch, to control blood glucose levels, is an effective strategy.^13,14^ It slows down carbohydrate digestion and reduces the spiking of glucose levels. To inhibit these enzymes, many organic moieties have been tried, but Schiff bases and their metal complexes are reported to be good enzyme inhibitors. Therefore, DM can be effectively controlled using these inhibitors.^15^

Schiff bases, named after the German scientist Hugo Schiff, are chemicals synthesized by the condensation reaction of a primary amine with a carbonyl molecule, often an aldehyde or a ketone. The fundamental structure of a Schiff base is the imine group, characterized by R_2_C=NR′ (where R′ is not hydrogen).^16^ Schiff bases of various organic moieties and resulting complexes have been reported to have diverse biomedical applications, like antimalarial, antifungal, antibacterial, anti-inflammatory, antiproliferative, antipyretic, and antiviral and antidiabetic.^16,17^ Among various organic moieties, the amantadine-derived compounds have attracted significant attention due to antimicrobial, anticancer, antiviral, antioxidant, and antidiabetic properties.^18^

Adamantane (C_10_H_16_) moiety having a highly symmetrical structure and low molecular weight provides a domain of highly critical lipophilicity, when inserted into the structure of any active drugs, and improves their lipophilicity and pharmacokinetic profiles.^19,20^ Several amino adamantanes (rimantadine, amantadine, memantine, tromantadine) have occupied a special place on the pharmaceutical market.^21^ Numerous biologically active amantadine-based complexes with the metals (Mg^2+^, Ca^2+^, Ni^2+^, Fe^3+^, Cr^3+^, Co^2+^, Mn^2+^, Cu^2+^, Cd^2+^, Fe^2+^, and Zn^2+^) have been prepared and are well-reported in the published literature.^22^ Few cobalt and cadmium complexes of adamantine-based derivatives were evaluated against *Artemia salina* and showed moderate activity.^23^ The complexes of platinum with amantadine (C_10_H_17_N) are adequately described due to their pharmaceutical significance as potent antineoplastic agents.^24^ Owing to the medicinal applications of this class of compounds, it has been anticipated that the adamantine-based Schiff bases and their metal complexes may act as efficient α-amylase and α-glucosidase inhibitors and can be effectively used to cure DM.

Following the above lead, and based on our previous work,^18^ a novel amantadine-derived ligand *via* condensation of 3-bromo-5-chloro-2-hydroxybenzaldehyde with the amantadine and its various transition compounds have been designed. The synthesized compounds were investigated *via* FT-IR, mass, NMR (^1^H & ^13^C), UV-visible, thermogravimetric, and elemental analyses (C/H/N). The crystalline structure of the ligand was also analyzed by single-crystal X-ray diffraction analysis. After successful characterization, the synthetic compounds have been evaluated for their potential to inhibit α-amylase and α-glucosidase by *in vitro* studies. Moreover, molecular docking studies were also performed to evaluate the specific interactions between synthesized compounds and α-amylase.

## 2 Experimental

### 2.1 Materials and methods

All the chemicals used for the designed work were of purely analytical grade. FT-IR spectral data were recorded on Shimadzu Prestige-21, while a UV 240 spectrophotometer (Shimadzu) was employed for recording UV-visible spectral data. Elico Conductivity Meter-CM82 was operated to analyze molar conductivity at 25 °C. Al sheets coated with silica gel (60 F254 Merck) were utilized to perform thin-layer chromatography. The mass spectral data was collected on an Agilent 6224, TOF-LC/MS system, both in +ve and −ve ions-ESI modes. NMR spectral data was accurately recorded on Bruker spectrometer at 400 MHz at the ambient temperature in deuterated DMSO. An elemental analyzer (Automatic CE-440-Exeter Analytical, Inc.) was utilized for C/H/N analysis, while melting temperatures were measured with Gallenkamp digital melting apparatus (MFB-595-010M). The X-ray crystallography was performed on the diffractometer (DS Venture-Bruker PHOTON II Detector) fitted with the micro-focus brilliance Mo radiation type at a temperature 273 K. TGA Q500-thermogravimetric analyzer was utilized to record the thermal degradation pattern at a precisely controlled heating rate (10 °C min^−1^). SpectraMax®, M2/M2e-microplate reader (Molecular Device Co., US) was employed to determine the α-amylase and α-glucosidase inhibition activities.

### 2.2 Preparation of Schiff base ligand (AHB)

**Scheme 1:**
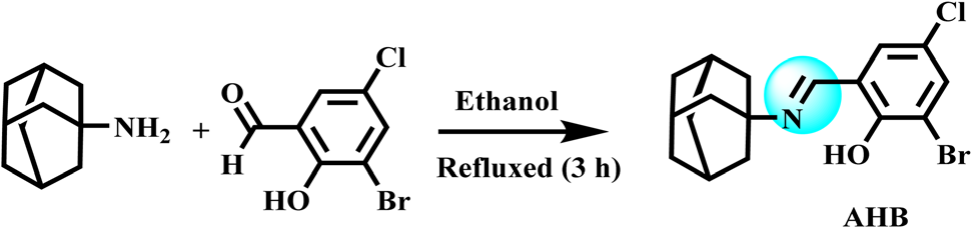
Preparation of (*E*)-2-((adamantan-2-ylimino)methyl)-6-bromo-4-chlorophenol (AHB).

The ligand and its transition metal complexes were prepared by adopting the reported methodology with a few modifications.^25^ A hot ethanolic solution (20 mL) of amantadine (6.6 mmol, 0.98 g) was added slowly into a reaction flask containing a hot ethanolic solution (25 mL) of 3-bromo-5-chloro-2-hydroxybenzaldehyde (6.6 mmol, 1.55 g) and refluxed for 3 h. On the reaction completion, the resultant solution was taken in a beaker and slowly evaporated for two days, resulting in the appearance of fine yellow crystals. These crystals were filtered off and washed thrice with chilled ethanol. The collected crystals of the ligand were later dried by placing them in a desiccator. The general method for the synthesis of AHB is illustrated in Scheme 1.

#### 2.2.1 AHB

Bright yellow; yield: 77 (%); mp: 145 °C; UV-Vis (nm): 269, 338, 433; ESI/MS: 370.04 [M + H]^+^; FT-IR (KBr, cm^−1^): 1627 (C=N), 1552 (C=C), 1213 (C–N), 729 (C–CI), 557 (C–Br), 2908, 2850 (C–H-aromatic, str); ^1^H-NMR data (DMSO-*d*_6_) (*δ*): 1.71–2.17 (m, CH and CH_2_– amantadine), 7.50 (s, Ar–H), 7.66 (s, Ar–H), 8.61 (s, N=CH benzylidenimin), 15.49 (s, OH); ^13^C-NMR (DMSO-*d*_6_); 29.09 (3C, adamantane), 35.76 (3C, adamantane), 42.19 (3C, adamantane), 56.72 (C–N=C), 115.64, 116.20, 117.31, 131.69, 135.93 (5C–Ar), 161.53 (C, phenolic OH), 165.54 (C=N); elemental analysis (CHN): calculated from chemical formula: C_17_H_19_BrCINO (exact mass: 367.03): C, 55.38; H, 5.19; N, 3.80; found; C, 55.42; H, 5.14; N, 3.82.

The structure of the ligand (AHB) was also confirmed by the single-crystal XRD data with a CCDC number of 2235552.

### 2.3 Preparation of metal complexes of AHB

**Scheme 2:**
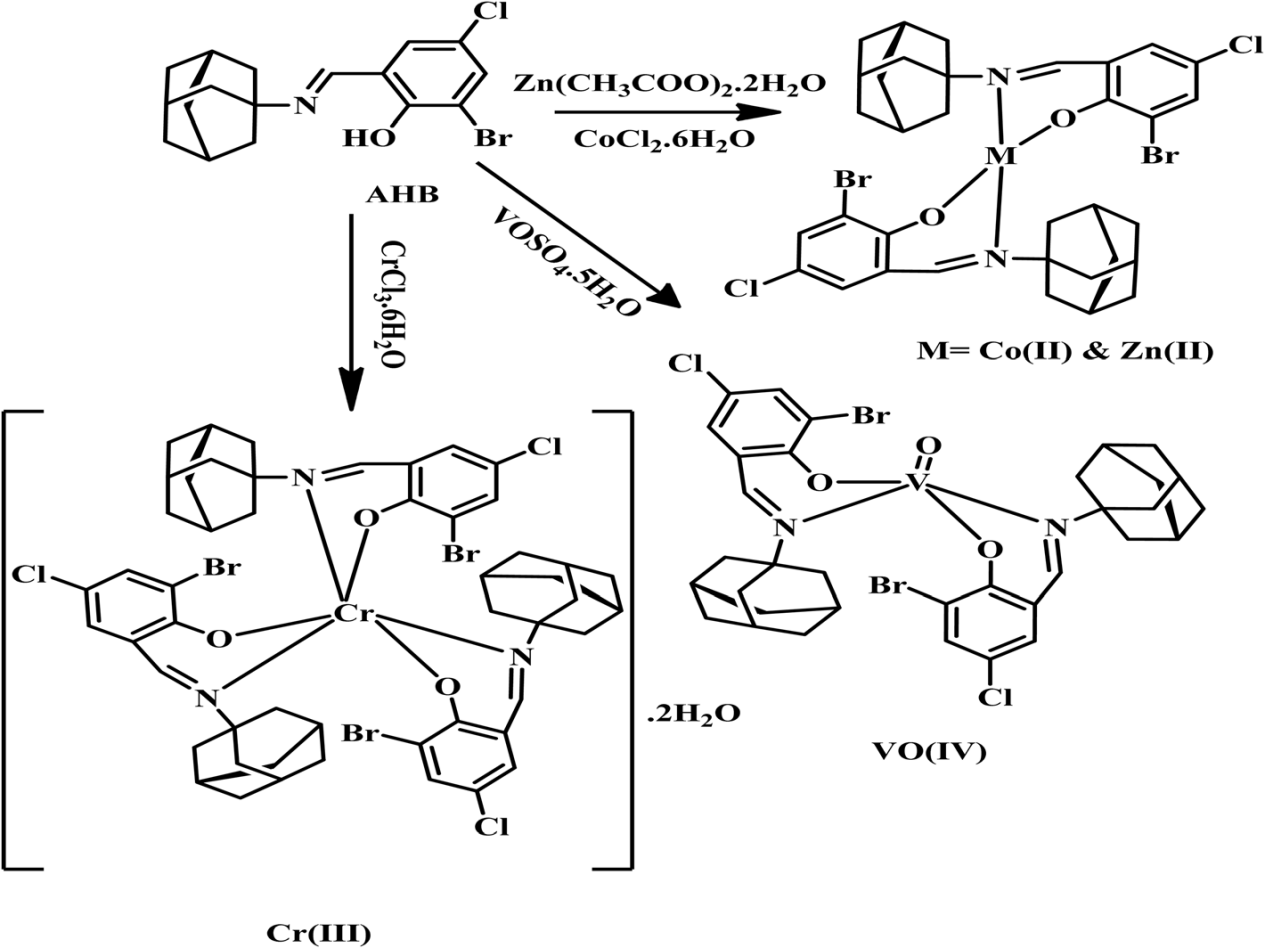
Synthesis of metal complexes of AHB.

The transition metal complexes of AHB were synthesized following the reported general procedure.^26,27^ Briefly, 15 mL of hot ethanol solution of respective metal salts like Zn(CH_3_COO)_2_·2H_2_O (0.21 g, 1 mmol), CoCl_2_·6H_2_O (0.23 g, 1 mmol), CrCl_3_·6H_2_O (0.26 g, 1 mmol), and VOSO_4_·5H_2_O (0.24 g, 1 mmol) was added dropwise in 15 mL of hot EtOH solution of AHB (0.46 g, 2 mmol) with constant stirring. The pH of all the resultant solutions was then adjusted between 8–8.5 with the help of 0.1% ethanolic solution of NaOH. Finally, the reaction solution was refluxed for 3–4 h. The final solution was then left at 37 °C overnight to get the precipitated complexes, which were then vacuum-filtered and washed three times with ice-cold EtOH. The route of synthesis of metal complexes of AHB is represented by Scheme 2.

#### 2.3.1 [Zn(AHB)_2_]

Yellow amorphous powder; mp: above 300 °C; yield: 67%; UV-Vis (nm): 275, 384, 450; molar conductivity (Ω^−1^ cm^2^ mol^−1^): 2.1; selected FT-IR (KBr, cm^−1^): 1610 (C=N), 1205 (C–N), 740 (C–CI), 555 (C–Br), 2910, 2850 (str, C–H, aliphatic), 501 (Zn–N), 424 (Zn–O); elemental analysis (CHN): chemical formula: C_34_H_36_Br_2_Cl_2_N_2_O_2_Zn (exact mass: 831.98): calculated: C, 51.00; H, 4.53; N, 3.50; found: C, 51.05; H, 4.55; N, 3.47.

#### 2.3.2 [Co(AHB)_2_]

Orange amorphous powder; mp: above 300 °C; yield: 56%; UV-Vis (nm): 325, 445, 602; *μ*_eff_: 3.87 B.M.; molar conductivity (Ω^−1^ cm^2^ mol^−1^): 3.9; selected FT-IR (KBr, cm^−1^): 1598 (C=N), 1205 (C–N), 742 (C–CI), 555 (C–Br), 2912, 2852 (str, C–H, aliphatic), 513 (Co–N), 445 (Co–O); elemental analysis (CNH): chemical formula: C_34_H_36_Br_2_Cl_2_N_2_O_2_Co (exact mass: 794.31): calculated: C, 51.41; H, 4.57; N, 3.53; found: C, 51.45; H, 4.52; N, 3.56.

#### 2.3.3 [Cr(AHB)_3_]

Green amorphous powder; mp: above 300 °C; yield: 34%; UV-Vis (nm): 335, 452, 600; *μ*_eff_: 3.87 B.M.; molar conductivity (Ω^−1^ cm^2^ mol^−1^): 4.2; selected FT-IR (KBr, cm^−1^): 1614 (C=N), 1209 (C–N), 746 (C–CI), 555 (C–Br), 2914, 2850 (str, C–H, aliphatic), 530 (Cr–N), 428 (Cr–O), 3323 (lattice H_2_O, broad); elemental analysis (CNH): chemical formula: C_51_H_54_Br_3_Cl_3_N_3_O_3_Cr (exact mass: 1158.66): calculated: C, 52.87; H, 4.73; N, 3.63; found: C, 52.88; H, 4.74; N, 3.65.

#### 2.3.4 [VO(AHB)_2_]

Dark green amorphous powder; yield: 70%; mp: above 300 °C; UV-Vis (nm): 324, 356, 440, 600; *μ*_eff_: 1.73 B.M.; molar conductivity (Ω^−1^ cm^2^ mol^−1^): 7.5; selected FT-IR (KBr, cm^−1^): 1625 (C=N), 1541 (C=C), 1139 (C–N), 736 (C–CI), 623 (C–Br), 2908, 2850.7 (str, C–H, aliphatic), 952 (V=O), 468 (V–O), 553 (V–N). CHN elemental analysis: chemical formula: C_34_H_36_Br_2_Cl_2_N_2_O_3_V (exact mass: 816.99): calculated: C, 50.90; H, 4.52; N, 3.49; found: C, 50.97; H, 4.64; N, 3.59.

### 2.4 Inhibition studies

#### 2.4.1 α-amylase inhibition activity

*In vitro* α-amylase inhibition studies were performed following a reported methodology with slight modification.^20^ First, to prepare our reaction mixture, 10 μL of enzyme solution (0.1 mg mL^−1^) was combined with a 10 mM phosphate buffer at pH 6.9 (6 mM NaCl), and then mixed with the various concentrations of the tested sample. The resultant reaction mixtures placed in a microplate were incubated for 30 minutes at 37 °C. After the incubation period, 40 μL of starch solution (1%) was added; subsequently, this assay plate was incubated at 37 °C for 10 minutes. Finally, the reaction was terminated by adding 20 μL of 1.0 M HCl to the prepared solution, followed by the addition of 75 μL of iodine solution. Quantification was performed spectrophotometrically at 580 nm. Acarbose at 50 μg mL^−1^ was used as a reference inhibitor in all reactions, and the tests were conducted in triplicate to obtain concordant results. Inhibition activity (%) was measured using eqn (1).1
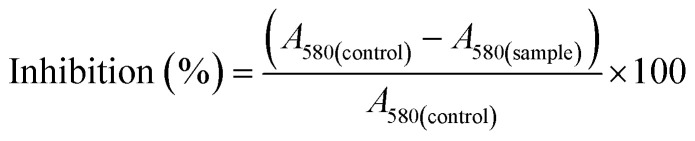
Here, the control sample excluded the α-amylase and the tested sample. The inhibitor concentration used to inhibit α-amylase activity (50%) under assay was determined as IC_50_ values. The findings were depicted as means ± SD values, obtained from microplate readers.

#### 2.4.2 α-glucosidase inhibitory activity

The previously reported methodology for α-glucosidase inhibition studies was followed with a few modifications.^21^ A test sample solution (10 μL) and phosphate buffer solution (30 μL, 0.1 M, pH 6.8) containing α-glucosidase (0.2 U mL^−1^, enzyme solution) were added to a 96-well microplate and incubated for 10 min at 37 °C. After the pre-incubation, pNPG (substrate, 30 μL, 5 mM) prepared in 0.1 M phosphate buffer (pH 6.8) was poured into the wells of the microplate, which were then incubated at 37 °C for 20 minutes. The enzymatic reaction was terminated by adding 40 μL of Na_2_CO_3_ (0.2 M) solution into each well of the plate, and the absorbance values (*A*) were measured at 405 nm with a 96-well plate reader, comparing with the control sample. Acarbose at 50 μg mL^−1^ was used as a reference inhibitor. For inhibition studies, various concentrations of test samples were utilized. The inhibition effect was expressed as % inhibitory activity and calculated according to eqn (2).2
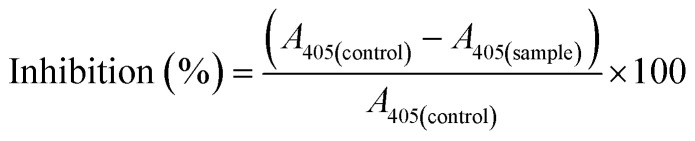


The inhibitor concentrations used under the assay environment for 50% inhibition of α-glucosidase activity were expressed as IC_50_ results. All data were presented as means ± SD values, obtained from a microplate reader for all experiments.

#### 2.4.3 Molecular docking analysis

Molecular docking analysis is an important tool to anticipate the plausible mode of action of organic and inorganic compounds toward specific proteins and enzymes and estimate the activity of synthesized compounds.^28^*In silico* antidiabetic efficacy of the synthesized compounds was evaluated to inhibit the normal functioning of α-amylase protein using a YASARA (Yet Another Scientific Artificial Real Application) software version 20.7.4.^29^ 3D structure of α-amylase was harvested from the protein databank. AutoDock LGA module and AMBER03 force fields in YASARA with 100 global docking runs and 1000 random seed values were used for docking. Then, the AutoDock local search (LGA-LS) method in YASARA was used to rescore docked compounds.^28^

## 3 Results and discussion

### 3.1 Characterization of (*E*)-2-((adamantan-1-ylimino)methyl)-6-bromo-4-chlorophenol (AHB) and its metal complexes

The amantadine-derived ligand (AHB) was prepared as shown in Scheme 1. In brief, amantadine was kept refluxed with 3-bromo-5-chloro-2-hydroxybenzaldehyde in an equimolar molar ratio using ethanol for 3 h, while metal complexes were prepared with the respective metal salts under reflux conditions in a basic environment by using 0.1% NaOH for 3 h.

### 3.2 UV-visible and magnetic moment studies

The UV-visible absorbance spectral data of ligand (AHB) and its coordinated complexes were collected in DMSO within the 200–800 nm range at 25 °C (Fig. S1†). For AHB, the bands observed around 269 nm are due to the aromatic system, while a band around 338 nm is attributable to the possible n → π* transitions because of the imine chromophore. A band that appeared around 430 nm exhibits n → π* transitions because of the charge transfer within the AHB, which may be due to the presence of conjugation. The spectrum of zinc complex displayed absorptions at 275, 384, and 450 nm due to the benzene group, –HC=N group, and charge transfer transitions, respectively. In the cobalt complex, the first absorption at 325 nm represents π → π* transitions due to the benzene ring, while the second absorption at 445 nm is due to the –HC=N group, which shows the n → π* transition, and the third absorption at 602 nm indicates charge transfer confirming the ligand metal coordination upon complexation. The Cr(iii) complex exhibits absorption bands at 335, 452, and 600 nm. The absorption band appeared around 452 nm may be because of the –HC=N group, which shows the n → π* transition, while the absorption at 600 nm is because of the charge transfer, showing the ligand coordination to the Cr(iii) in the complex. The oxovanadium complex displayed absorption bands at 324, 356, 440, and 600 nm, which show the transition due to –HC=N chromophore, charge transfer, and possible d–d transition. It was observed that in all metal complexes, absorption corresponding to the –HC=N group exhibited a red shift toward a longer wavelength (lower energy) as compared to the ligand upon metal complexation. This red shift indicates the ligand coordination to the metal ion *via* the –HC=N group.^30^

The prepared Cr(iii) complex was found to have a magnetic moment value of 3.87 B.M., which indicates the presence of three unpaired electrons, which is characteristic of the octahedral geometry of Cr(iii) complexes.^31^ The observed magnetic moment of the V(iv) complex is 1.73 B.M., which falls within the range mostly observed for square pyramidal geometry.^32^ The magnetic moment value for the Co(ii) complex is 4.58 B.M., which agrees well with the known values for Co(ii) complexes in square planar geometry.^39^ The Zn(ii) complex is diamagnetic due to the absence of unpaired electrons.^40^

### 3.3 FT-IR spectral studies

FT-IR spectral data provide a piece of detailed information to explain AHB coordination to the selected metal ions. The observed stretching vibrations of the synthesized Schiff base and its transition metal complexes are shown in Table 1. The FT-IR spectral data of AHB (Fig. S2†) shows stretching vibrations for the OH group at 3234 cm^−1^. This OH group may be involved in intramolecular hydrogen bonding.^27^ The disappearance of OH-group stretching vibrations in all coordinated complexes (Fig. S3–S6†) indicates that the OH group is involved in metal complexation. A strong band that was detected at 1627 cm^−1^ for the free ligand is attributed to the azomethine (–HC=N) vibration modes.^17^ A decrease (10–29 cm^−1^) in stretching vibrations was noticed for the –HC=N group on metal complexation, which indicates that the azomethine group gets involved in the metal coordination *via* nitrogen by the decreasing electron density on metal ions, while in the case of vanadium (Fig. S6†), it showed minor decreased in stretching frequency by 2 cm^−1^. The complexation was supported further by the decrease in the frequencies of the C–N group from ∼1213 cm^−1^ (in ligand) to a lower stretching frequency of 1139 to 1209 cm^−1^ (in all complexes). New bands appeared in 424–468 cm^−1^ may be because of observed stretching vibrations for the metal–oxygen (M–O) bond. The bands, which appeared at 501–553 cm^−1^ may be because of observed metal–nitrogen (M–N) stretching vibrations in complexes.^33–35^ In the FT-IR data of the oxovanadium complex (Fig. S6†), a distinctive peak was displayed at 952 cm^−1^, which is because of the stretching vibrations of the V=O bond, confirming the vanadium complexation.^36^ The FT-IR spectrum of chromium coordinated complex exhibited a broad peak around 3323 cm^−1^, which is given to *ν*(–OH) stretching vibrations of the attached water molecule, which shows the existence of lattice or coordinated H_2_O molecules.^37^

**Table 1:** FT-IR absorption bands of ligand (AHB) and its coordination complexes (cm^−1^)

Compounds	HC=N	C–N	M–N	M–O	H_2_O	OH	V=O
AHB	1627	1213	—	—	—	3234	—
[Zn(AHB)_2_]	1610	1205	501	424	—	—	—
[Co(AHB)_2_]	1598	1205	513	445	—	—	—
[Cr(AHB)_3_]	1614	1209	530	428	3323	—	—
[VO(AHB)_2_]	1625	1139	553	468	—	—	952

### 3.4 Mass spectrometric studies

LC-MS spectral data of AHB, as shown in Fig. S7,† indicate the molecular ion M^+^ peak at *m*/*z* = 370.04, which confirms the proposed molecular formula and structure of the ligand (AHB). Appeared molecular ion M^+^ peak is displayed as a base peak, while its intensity shows stability along with its abundance. The calculated molecular ion was found in good agreement with the expected structure of the designed ligand.

### 3.5 NMR spectral studies

**Fig. 1:**
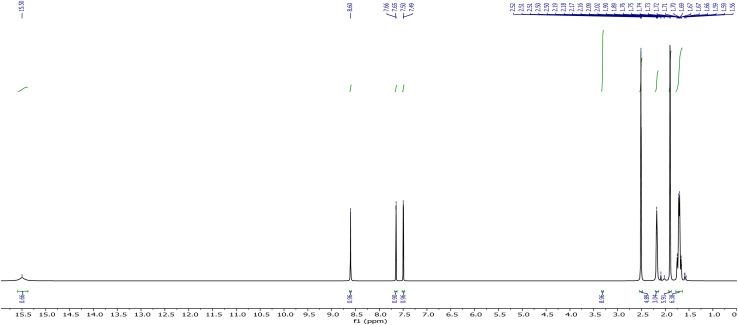
^1^H-NMR spectrum of (*E*)-2-((adamantan-2-ylimino)methyl)-6-bromo-4-chlorophenol (AHB).

**Fig. 2:**
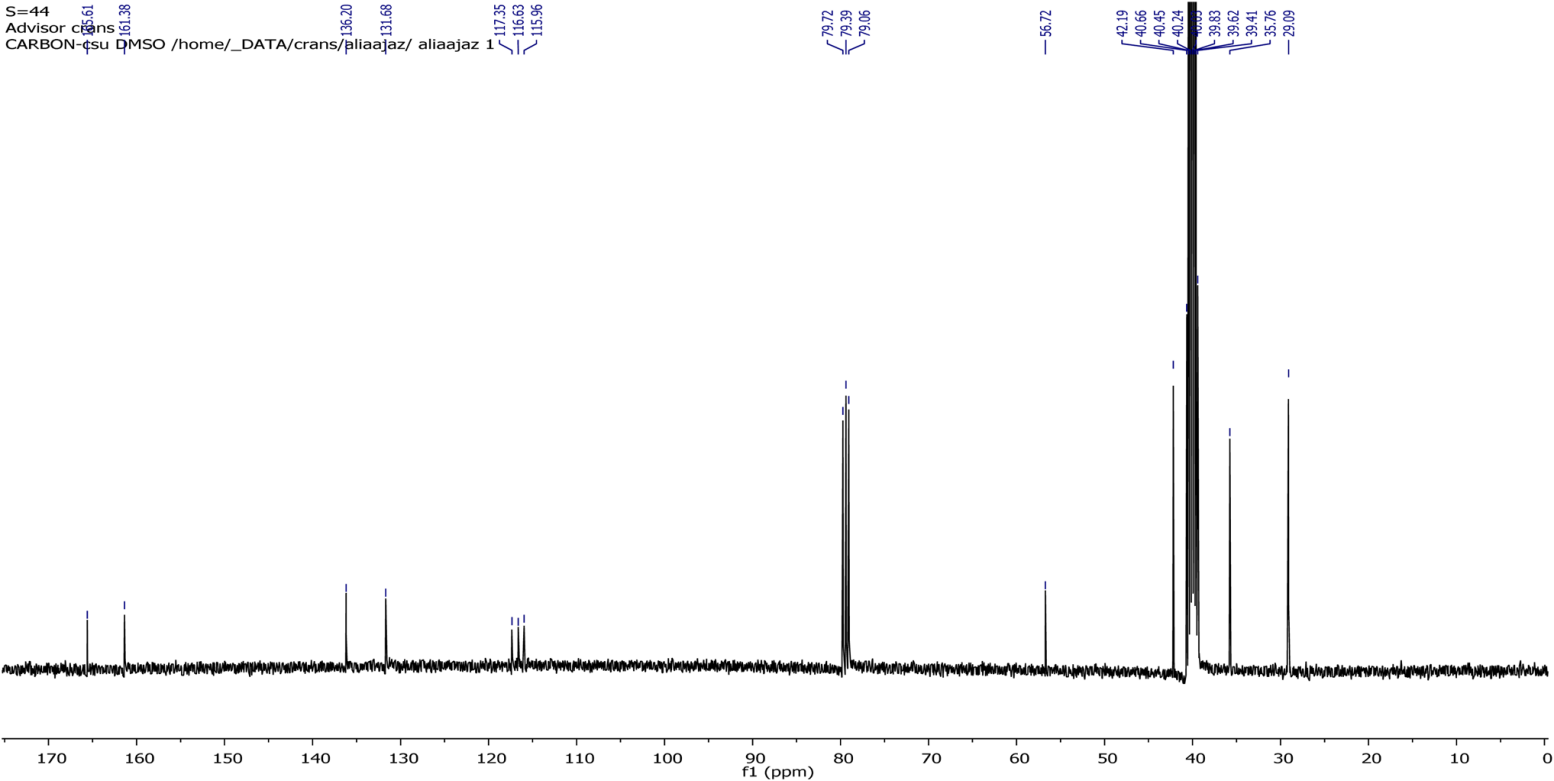
^13^C-NMR spectrum of (*E*)-2-((adamantan-2-ylimino)methyl)-6-bromo-4-chlorophenol (AHB).

The NMR data of AHB have been recorded in deuterated DMSO, and both spectra are given in Fig. 1 and 2. The proton NMR spectrum of the prepared compound (AHB) displayed its aliphatic and aromatic ring protons (Ar–H). The signal at 15.49 ppm for the ligand is attributed to the phenolic (–OH) proton. Two doublets at 7.50 and 7.65 ppm [^5^*J* = 0.4 Hz] are due to the ring aromatic protons. A sharp singlet that appeared at 8.61 ppm is assigned to the –HC=N proton.^38^ The aliphatic protons of amantadine were identified at 1.71–2.17 ppm.

In the case of ^13^C-NMR spectral data of AHB, the carbon atoms of amantadine appeared at 29.02, 35.60, and 42.27 ppm, however, the carbon atom of amantadine bonded to the nitrogen of the –HC=N– group displayed a signal at 56.73 ppm. The carbon atom of the imine group exhibited a strong signal at 165.54 ppm, while the carbon atom of the ring directly linked to the OH gave a signal at 161.53 ppm. The aromatic carbons displayed signals at 115.64, 116.63, 117.31, 131.69, and 136.20 ppm.^39^

### 3.6 Single crystal XRD analysis for ligand

The bright yellow crystals of the AHB were grown in an ethanolic medium. A better-quality crystal was selected for the X-ray crystallography. The selected crystal was then fixed on a goniometer head by utilizing grease. The resultant data was later recorded through Bruker's D8 Venture, having a PHOTON II type detection system and high brilliance Mo micro-focus (IμS) source (*λ* = 0.71073 Å) (Bruker, 2016), at the ambient conditions under the cooling Oxford Cobra Cryosystem by using both omega and phi scanning methods. APEX3 crystallography tool was used to process the diffracted intensities.

Multiscan absorption corrections were performed. Table 2 sums up the crystallographic details along with refinement information. The crystalline structure was solved into the monoclinic space group for AHB by using SHELXL (Sheldrick, 2008). All of the hydrogen atoms could be positioned into different electron density-Fourier maps, although they were fixed utilizing the riding structure model.

**Table 2:** Single crystal XRD details of (*E*)-2-((adamantan-2-ylimino)methyl)-6-bromo-4-chlorophenol (AHB)

Parameter	Value
CCDC	2235552
Chemical formula	C_17_H_19_BrClNO
*M*_r_	368.69
Crystal system, space group	Monoclinic, *P*2_1_
Temperature (K)	273
*a*, *b*, *c* (Å)	8.669 (3), 6.8018 (18), 13.799 (4)
*β* (°)	102.534 (8)
*V* (Å^3^)	794.2 (4)
*Z*	2
Radiation type	Mo Kα
*μ* (mm^−1^)	2.75
Crystal size (mm)	0.3 × 0.11 × 0.05
Diffractometer	Bruker's D8 Venture
Absorption correction	Multi-scan
No. of measured, independent and observed [*I* > 2*σ*(*I*)] reflections	18 535, 3213, 1808
*R*_int_	0.062
(sin *θ*/*λ*)_max_ (Å^−1^)	0.625
*R*[*F*^2^ > 2*σ*(*F*^2^)], w*R*(*F*^2^), *S*	0.137, 0.429, 1.54
No. of reflections	3213
No. of parameters	191
No. of restraints	1
H-atom treatment	H atom parameters constrained
Δ*ρ*_max_, Δ*ρ*_min_ (e Å^−3^)	2.32, −1.56

**Fig. 3:**
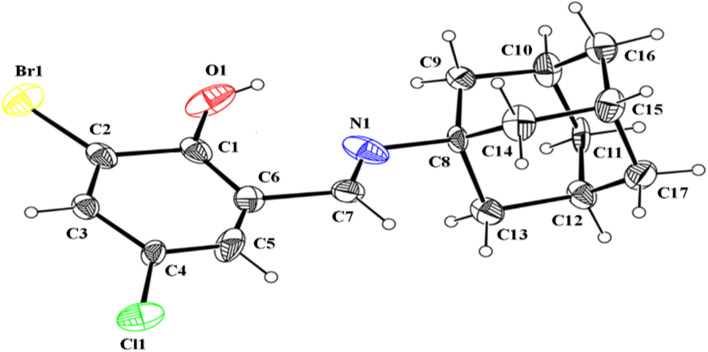
ORTEP representation of (*E*)-2-((adamantan-2-ylimino)methyl)-6-bromo-4-chlorophenol (AHB) drawn at a 20% probability.^49^

**Fig. 4:**
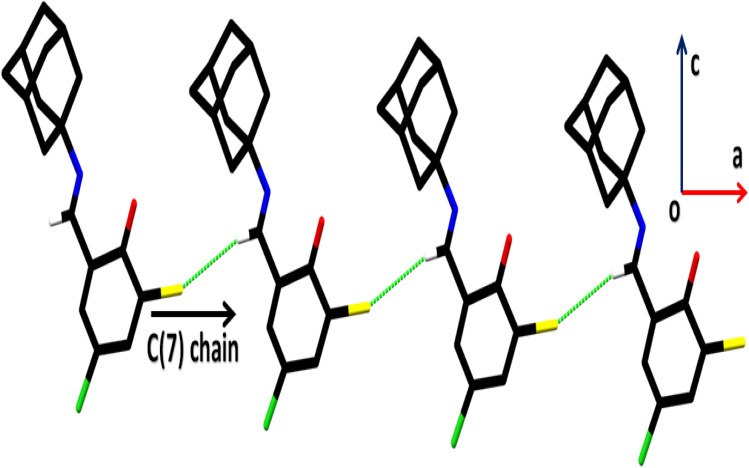
Packing diagram of AHB. Only selected hydrogen atoms are displayed for simplicity.

**Fig. 5:**
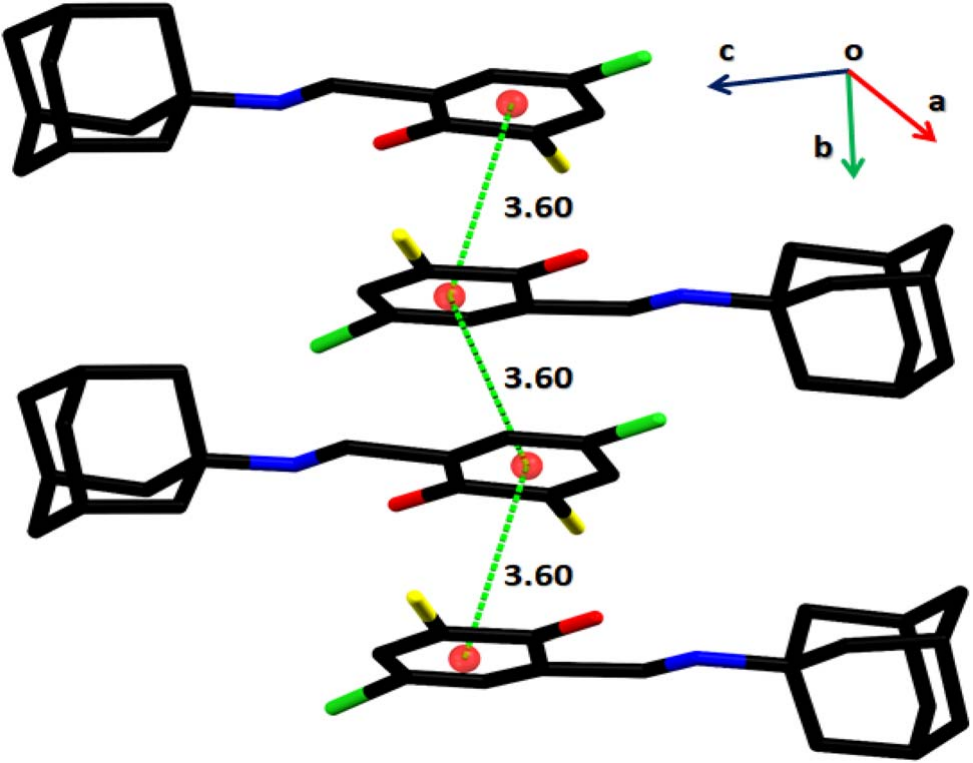
Graphical demonstration of off-set π⋯π stacking interactions in AHB. H-atoms are omitted for clarity, and the distances are dignified in Å.

In the crystal structure of (*E*)-2-((adamantan-2-ylimino)methyl)-6-bromo-4-chlorophenol (AHB) (Fig. 3 and Table 2), the substituted phenyl and amantadine rings are bridged by the imine group. The aldehyde part (C1–C7/N1/O1/Cl1/Br1) is nearly planar. The average C–C bond length in the amantadine ring (C8–C17) is 1.52 Å. An enol tautomeric form is adopted by the molecule with O–H⋯N bonding and constructing an S (6) loop. A similar S (6) H-bonded loop is noticed in the related crystal structure by Cambridge structural database search on CSD 2022, September 2022.^40–42^ No conventional intermolecular hydrogen bonding is the prominent point of the crystal packing. The molecules of the AHB are interlaced *via* C–H⋯Br bonding and form a C(7) chain that spreads alongside the *a*-axis (Fig. 4 and Table S1†), where CH is from the imine group (C7/N1). The offset π⋯π stacking interactions among six-membered rings is the additional strengthening factor of the crystal packing with the distance shown in Fig. 5. As a result of this interaction, a molecular chain is built up that runs alongside the *b*-axis (Fig. 5).

### 3.7 Hirshfeld surface analysis

**Fig. 6:**
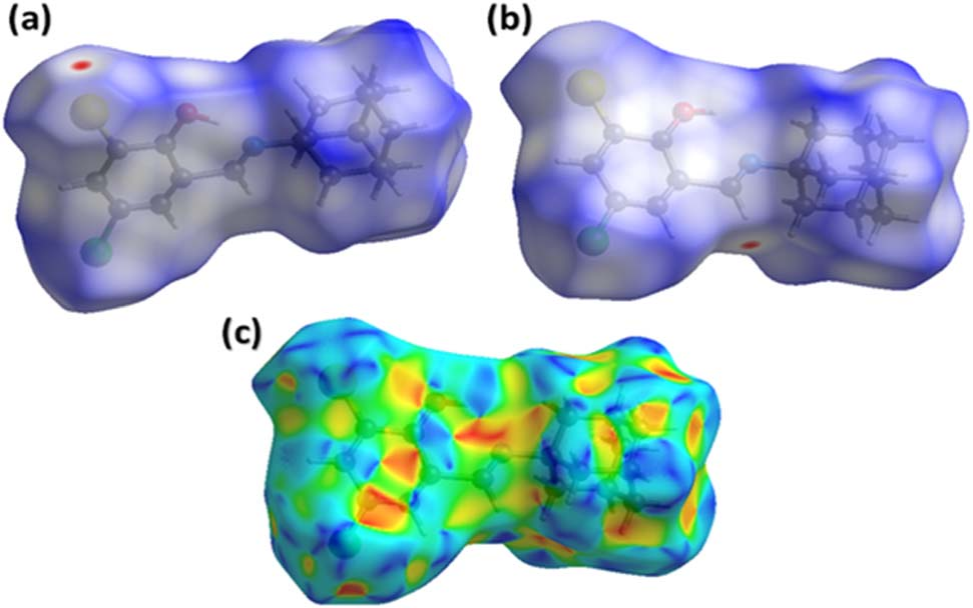
Hirshfeld surfaces plotted over *d*_norm_ in the range from 0.0714 to 1.4812 a.u., (a) first view, (b) second view. (c) Hirshfeld surface plotted over shape index in the range from −1 to 1 a.u.

The main curiosity of the scientists working in the supramolecular chemistry arena is to gain knowledge about the intermolecular interactions present in a single crystal. The properties of the single crystals are dependent on intermolecular interactions. Under that perception, an attempt is made from our side to reconnoitre the intermolecular interactions in AHB by carrying out a Hirshfeld surface analysis using Crystal Explorer version 21.5.^43^ Hirshfeld surface conspired over normalized distances (*d*_norm_) is the separator of the shorter contacts from the longer ones.^44,45^ Two different views of the surface (Fig. 6a and b) are added as it is impossible to show all red spots on one surface because of graphical limitations. The red area around CH of the imine is the H-bond donor indicator (Fig. 6a), whereas the red spot around the Br-atom is the H-bond acceptor indicator (Fig. 6b). The white region around the substituted phenyl ring is the indicator of the involvement of this ring in an interaction weaker than H-bonding. That interaction is confirmed by repeated red and blue triangular-shaped areas around the aromatic ring on the Hirshfeld surface plotted over the shape index (Fig. 6c).

**Fig. 7:**
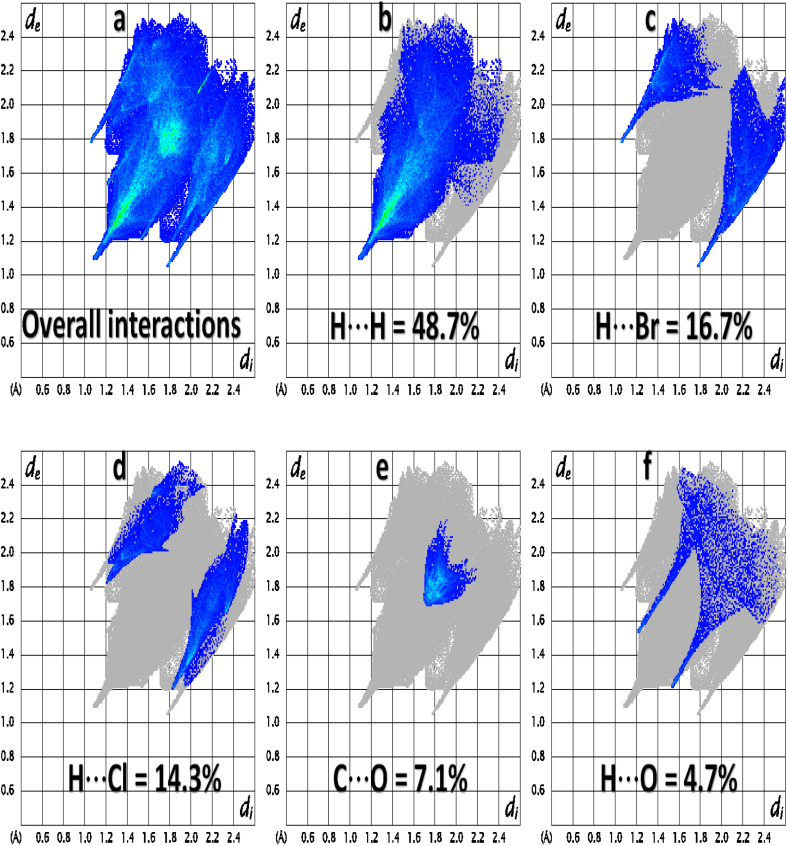
Important 2D fingerprint plots of (*E*)-2-((adamantan-2-ylimino)methyl)-6-bromo-4-chlorophenol (AHB). 2D plots for (a) Overall interactions, (b) H⋯H interactions, (c) H⋯Br interactions, (d) H⋯Cl interactions, (e) C⋯O interactions, (f) H⋯O interactions.

For a single crystal, each interatomic connection has an exceptional contribution to the supramolecular assembly. Some contacts have significant contributions, while others have negligible small contributions.^46,47^ 2D fingerprint plots provide support for the contacts in the crystal packing. Fig. 7a is the 2D plot for overall interactions on which the central sky blue triangular-shaped region shows the contribution of C⋯C contacts. The H⋯H contact is the supreme important contact with the support of 48.7% in the crystal packing and has the equal smallest value for *d*_i_ and *d*_e_ (1.1 Å) as shown in Fig. 7b. Regardless of the above contact, H⋯Br, H⋯Cl, C⋯O, and H⋯O are imperative with support shown in (Fig. 7c–f). The enrichment ratio is assimilated by dividing the actual contribution of the pair in the crystal packing with the corresponding value of the random contact. The C⋯C contact is the most probable with an enrichment ratio of 7.32 (Table S2†).

The properties that a single crystal holds are influenced by its packing efficiency. A crystal, which has a small value of packing efficiency will have a poor mechanical response, *i.e.*, the response in the case of applied stress. To find the packing efficiency, we calculated the voids in AHB. The spherical electron density of the atoms placed at the suitable positions is added up for the calculation of voids by using the idea of procrystal electron density.^48^ Fig. S8† shows two views of the voids in AHB. The volume of voids is found to be 108.81 Å^3^, which inferred that the crystal efficiency is 86.3%. Since the packing efficiency is high suggesting that the crystal has no large cavity and is predicted to exhibit good mechanical properties like melting point, elastic constant *etc.*

**Fig. 8:**
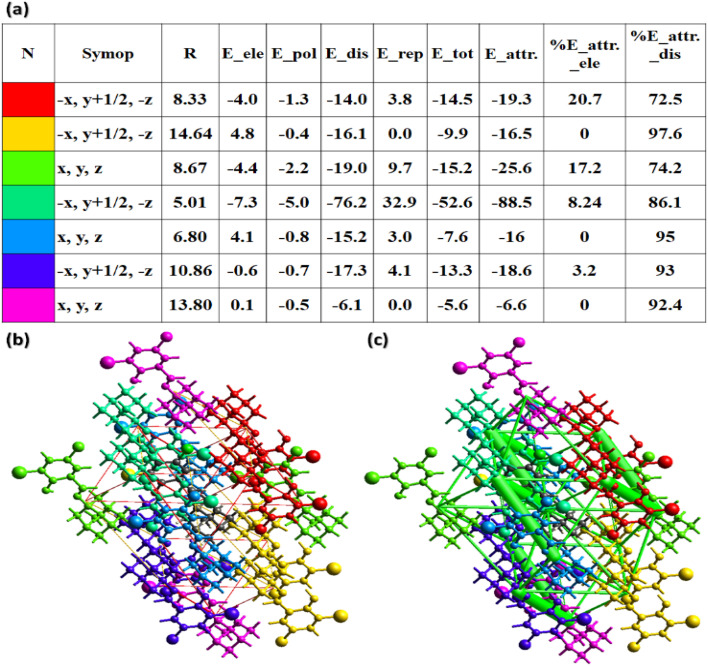
(a) Graphical view of the output obtained for the interaction energies between molecular pairs, (b) energy framework for coulomb energy, (c) energy framework for dispersion energy in the ligand (AHB) crystal structure.

The crystal packing can be additionally investigated through the interaction energy calculations by using the HF/3-21G electron density model on Crystal Explorer version 21.5.^49^ A 3.8 Å cluster of molecules is generated to carry out the calculations. Additional details are listed in Table S3.† The coulomb energy may be positive (repulsive) or negative (attractive), whereas dispersion energy is always attractive. These two energies play a significant role in crystal packing as compared to other kinds of energies. The Coulomb energy is attractive for all the molecular pairs excluding the pairs with intermolecular distances of 14.64, 6.80, and 13.80 Å (Fig. 8a). Coulomb and dispersion energies have the greatest contribution for the pairs with distances of 8.33 and 14.64 Å. In order to further distinguish the involvement of coulomb and dispersion energies in the crystal packing, energy frameworks are constructed.^50^ The distance between the molecular pairs is denoted by cylinders and the width of the cylinders is proportional to the strength of the interaction (Fig. 8b and c). The width of the cylinders indicates that the dispersion energy performed a dominant part in the stabilization of the crystal packing.

### 3.8 Molar conductivity measurement

Measurement of the molar conductance of prepared coordinated compounds was done in DMSO at a concentration of 1 × 10^−3^ mol dm^−3^. The measured conductivity values of prepared chelates were found within the 2–7 ohm^−1^ cm^2^ mol^−1^ range, which indicated the non-electrolytic nature of metal chelates.^51^

### 3.9 Thermal analysis of AHB and metal complexes

**Fig. 9:**
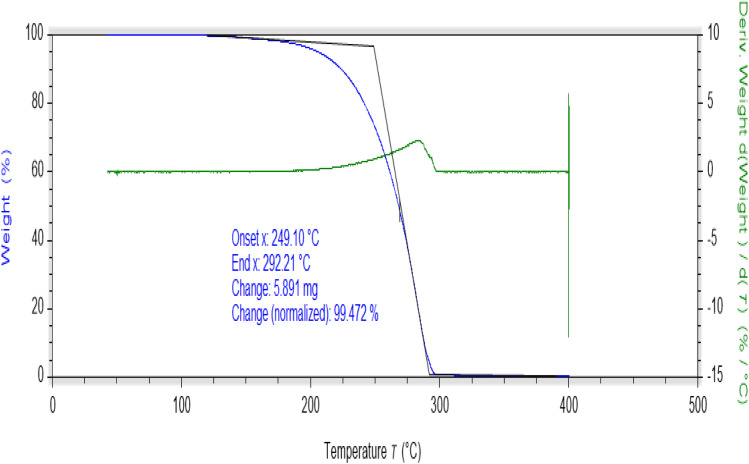
Thermogram of (*E*)-2-((adamantan-1-ylimino)methyl)-6-bromo-4-chlorophenol (AHB).

Thermogravimetric (TGA) studies were performed at a suitably controlled heating rate (10 °C min^−1^) as a function of temperature under the nitrogen gas flow. The thermogram of AHB is shown in Fig. 9, while the thermograms of coordinated compounds are shown in Fig. S9–S12.† Thermal degradation of AHB occurred completely in one step in the range of 249–292 °C, while the prepared metal complexes were thermally degraded in multiple steps (Fig. S8–S11†). The thermal degradation study of the Zn(ii) complex has shown two thermal steps. The first thermal degradation occurred within the range of 393–403 °C, indicating the removal of substitutions, while the second decomposition, around 413–428 °C, indicated the loss of ligand molecules, leading to the metal oxide formation. On the other side, thermal degradation of the cobalt complex occurred in two steps, around 253–390 °C and 404–472 °C. The first thermal step indicated the elimination of substituents, while the next step involved the remaining loss of ligand. A thermogram of chromium indicated the thermal decomposition in three thermal steps. The first stage of degradation shows the removal of lattice water molecules,^52^ while the second stage indicates the loss of substitutions in the range of 175–262 °C. The third decomposition step showed the removal of another thermally degradable part in the range of 475–558 °C. The thermogram of the oxovanadium complex exhibited two decomposition steps around 394–408 °C and 421–464 °C, which involved the removal of substitutions and other degradable moieties leading to the oxide formation above 464 °C. Chromium and cobalt complexes were not found to decompose completely even at 900 °C, some undecomposed mass was left behind therefore final decomposition residue was not possible to be measured. All of the prepared complexes were found thermally more stable as compared to the AHB.

### 3.10 Assay for α-glucosidase and α-amylase inhibitory studies

**Fig. 10:**
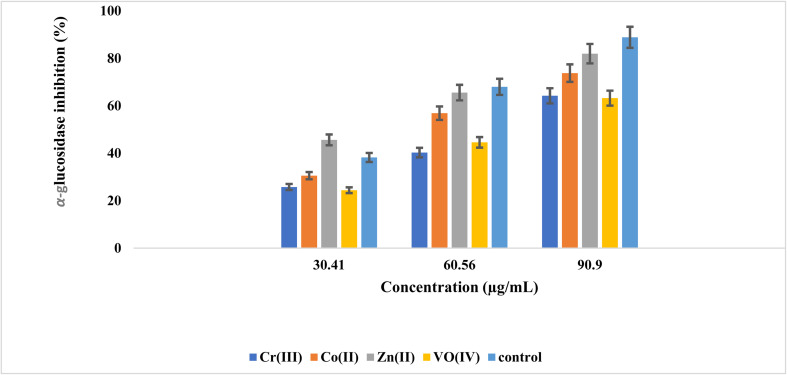
Concentration-dependent inhibition of α-glucosidase by metal complexes of (*E*)-2-((adamantan-1-ylimino)methyl)-6-bromo-4-chlorophenol (AHB).

**Fig. 11:**
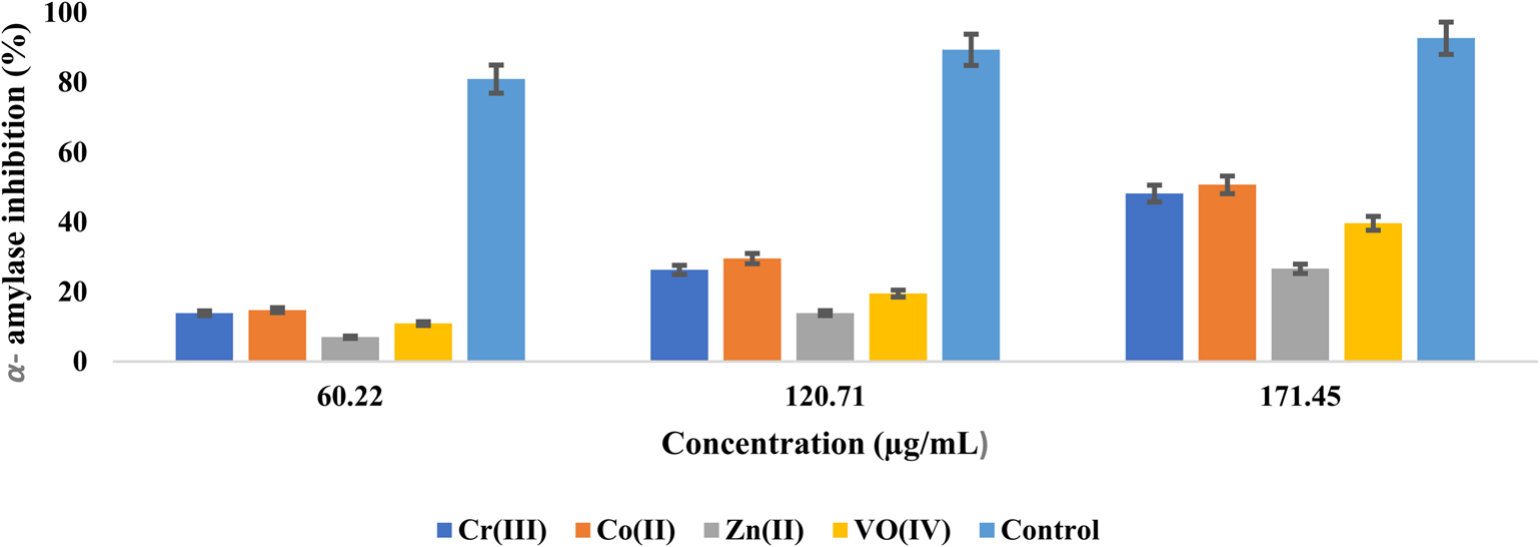
Concentration-dependent inhibition of α-amylase by metal complexes of (*E*)-2-((adamantan-1-ylimino)methyl)-6-bromo-4-chlorophenol (AHB).

α-amylase and α-glucosidase are fundamental enzymes that are responsible for the carbohydrate breakdown into glucose. α-amylase causes the hydrolysis of starch into glucose, while α-glucosidase involves the cleavage of disaccharides into glucose before absorption.^53,54^ DM can be controlled by lowering the glucose cellular uptake through the inhibitory enzymes mainly involved in carbohydrate breakdown, and these enzyme activities can be inhibited by using potent inhibitors to treat hyperglycemia.^55^ The α-glucosidase inhibitory effect was determined at concentrations of 30.41, 60.56, and 90.90 μg mL^−1^, and α-amylase inhibitory effects were measured at 60.22, 120.71, and 171.45 μg mL^−1^. The obtained inhibition data are depicted in Fig. 10 and 11. Both inhibition studies showed an increase in activity with the increase in inhibitor concentration, which illustrated a robust inhibitor dose-dependent response. The screening findings against the α-glucosidase demonstrated high inhibitory action of zinc complex, having an IC_50_ calculation of 73.08 ± 0.99 μg mL^−1^, then by Co(ii), oxovanadium, and chromium complexes with IC_50_ values of 105.15 ± 1.78, 133 ± 1.99, and 136 ± 1.99 μg L^−1^, respectively. High inhibitory action against α-amylase was exhibited by the Co complex with an IC_50_ value of 136.78 ± 2.03 μg mL^−1^, subsequently by the chromium, oxovanadium, and zinc complexes having IC_50_ values of 139.30 ± 2.53, 169.97 ± 4.43, and 258.78 ± 7.29 μg mL^−1^, respectively, as given in Table 4. The literature-based analogous amantadine-based compounds supported the above results, showing their strong inhibition effect against α-amylase and α-glucosidase enzymes.^16^

### 3.11 Molecular docking results

*In silico* interactions of synthesized drug candidates with the targeted enzymes is a rational strategy to anticipate the mode of action of drugs.^28^ α-amylase is actively involved in the breakdown of sugar moieties and increases the blood glucose level by abrupt action on carbohydrates. The blood glucose level can be sufficiently controlled by inhibiting the activity of this enzyme, resulting in the slow production of glucose and controlled release.

**Fig. 12:**
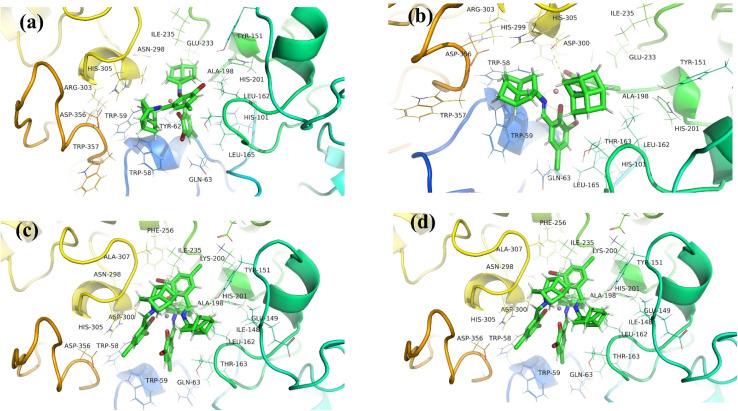
Molecular docking interactions of α-amylase and synthesized complexes, (a) Co(AHB)_2_, (b) Zn(AHB)_2_, (c) Cr(AHB)_3_, and (d) VO(AHB)_2_.

The synthesized complexes showed good binding energies with the active sites of α-amylase, as shown in Fig. 12. The docking score values and mode of binding interactions are shown in Table 3. Among all the complexes, the Zn(AHB)_2_ complex showed highest binding score (−9.573 kcal mol^−1^) with side chain amino acids (TRP-58, TRP-59, TYR-62, GLN-63, TYR-151, LEU-162, THR-163, LEU-165, ARG-195, ASP-197, ALA-198, HIS-201, GLU-233, HIS-299, ASP-300, ARG-303, HIS-305, ASP-356, and TRP-357) of α-amylase with minimum dissociation constant value (96.138 nM). Many types of interactions including H-bonding, hydrophilic, and hydrophobic interactions were observed between complex and protein molecule. Following the Zn(AHB)_2_ complex, VO(AHB)_2_ complex showed good binding score of −9.302 kcal mol^−1^, with amino acid side chains (TRP-58, TRP-59, TYR-62, GLN-63, HIS-101, TYR-151, LEU-162, THR-163, LEU-165, ARG-195, ASP-197, ALA-198, LYS-200, HIS-201, GLU-233, ILE-235, HIS-299, HIS-305, and GLY-306) with dissociation constant of 151.895 nM. However, a minimum binding score was observed between α-amylase and Co(AHB)_2_ followed by Cr(AHB)_3_ complex with binding constant values of −9.097 kcal mol^−1^ and −8.378 kcal mol^−1^, respectively. Main amino acids involved in interactions between α-amylase and Co(AHB)_2_ are TRP-58, TRP-59, TYR-62, GLN-63, LEU-162, THR-163, LEU-165, HIS-201, GLU-233, ILE-235, ASP-300 and HIS-305 and amino acids involved in interactions between α-amylase and Cr(AHB)_3_ are ILE-148, TYR-151, LEU-162, THR-163, ALA-198, LYS-200, HIS-201, GLU-233, ILE-235, ASP-300, GLY-306, ALA-307, and ASP-356. The enzyme inhibitory potential of the metal complexes is greatly varied on variation in coordination number, geometry, charge, and electronic properties of the central metal, rigidity of the ligand, and stability of the complex. In molecular docking studies, it was observed that the binding energy of the tetrahedral complex [Zn(AHB)_2_] with the α-amylase was superior to the octahedral complex [Cr(AHB)_3_]. Moreover, it was also observed that the size played the main role in the binding of complexes with amino acids in the grooves of the protein. Smaller-sized complexes have shown maximum interaction with the amino acids by adjusting in the grooves of proteins as shown by the [Zn(AHB)_2_], [VO(AHB)_2_] and [Co(AHB)_2_] with binding energies of −9.573, −9.302, and −9.097 kcal mol^−1^, respectively as compared to large sized octahedral complex [Cr(AHB)_3_] with binding energy value of −8.378 kcal mol^−1^. Although these results contradict the *in vitro* studies, however, these contradictions can be observed due to different environmental conditions during experimental *in vitro* studies, like pH, temperature, *etc.* than *in silico* findings.

**Table 3:** Molecular docking results of interactions between amino acids of α-amylase and synthesized complexes

Sr. no.	Compound	Protein	Binding energy (kcal mol^−1^)	Dissociation constant (nM)	Main contacting amino acid residues
1	Co(AHB)_2_	α-amylase	−9.097	214.698	TRP-58, TRP-59, TYR-62, GLN-63, LEU-162, THR-163, LEU-165, HIS-201, GLU-233, ILE-235, ASP-300 & HIS-305
2	Zn(AHB)_2_		−9.573	96.138	TRP-58, TRP-59, TYR-62, GLN-63, TYR-151, LEU-162, THR-163, LEU-165, ARG-195, ASP-197, ALA-198, HIS-201, GLU-233, HIS-299, ASP-300, ARG-303, HIS-305, ASP-356 & TRP-357
3	Cr(AHB)_3_		−8.378	722.511	ILE-148, TYR-151, LEU-162, THR-163, ALA-198, LYS-200, HIS-201, GLU-233, ILE-235, ASP-300, GLY-306, ALA-307 & ASP 356
4	VO(AHB)_2_		−9.302	151.895	TRP-58, TRP-59, TYR-62, GLN-63, HIS-101, TYR-151, LEU-162, THR-163, LEU-165, ARG-195, ASP-197, ALA-198, LYS-200, HIS-201, GLU-233, ILE-235, HIS-299, HIS-305 & GLY-306

**Table 4:** α-glucosidase and α-amylase inhibition activities (%)

Compounds	IC_50_ (μg mL^−1^)	
	α-glucosidase	α-amylase
[Zn(AHB)_2_]	73.08 ± 0.99	258.78 ± 7.29
[Co(AHB)_2_]	105.15 ± 1.78	136.78 ± 2.03
[Cr(AHB)_3_]	136.15 ± 2.74	139.30 ± 2.53
[VO(AHB)_2_]	133 ± 1.99	169.97 ± 4.43
Control	84.15 ± 1.20	78.57 ± 0.99

## 4 Statistical analysis

For each experiment, analysis was performed in triplicates. Statistical findings were studied with a statistical method (ANOVA). Statistical significance at a level of *p* < 0.05 was accepted.

## 5 Conclusions

In conclusion, a novel crystalline ligand (AHB) and its metal compounds were synthesized. All prepared compounds were characterized successfully by using different spectroscopic techniques, mass spectrometry, and elemental analysis. Crystallographic data of the synthesized ligand was investigated that showed the monoclinic, *P*2_1_ system of crystals of AHB. Molar conductivity data revealed that all complexes are non-electrolytes, while the thermal decomposition pattern of prepared metal complexes showed better thermal stability of complexes than ligand. [Zn(AHB)_2_] exhibited good α-glucosidase activity, while other complexes were found to have moderate inhibition against α-glucosidase. While [Cr(AHB)_3_] and [Co(AHB)_2_] were determined to be potent α-amylase inhibitors. The present research work findings demonstrated that the synthesized ligand and its metal complexes can be used as effective antidiabetic agents by inhibiting α-glucosidase and α-amylase.

## Data availability

All evaluated data are available in the manuscript. Additional information/data can be provided upon reasonable request.

## Author contributions

Conceptualization and methodology, Aliya Ajaz and Muhammad Ashraf Shaheen; software, Aliya Ajaz, Muhammad Fayyaz ur Rehman, Maqsood Ahmad, Muhammad Ashfaq; validation, Aliya Ajaz, Muhammad Fayyaz ur Rehman, Khurram Shahzad Munawar and Nazir Ahmad; formal analysis, Aliya Ajaz, Khurram Shahzad Munawar and Nazir Ahmad; investigation, Aliya Ajaz, Muhammad Fayyaz ur Rehman, Muhammad Ashfaq and Maqsood Ahmad; original draft preparation, Aliya Ajaz and Khurram Shahzad Munawar; writing—review and editing, Aliya Ajaz, Abu Bakar Siddique, Muhammad Fayyaz ur Rehman, Muhammad Ahshraf Shaheen, Khurram Shahzad Munawar and Nazir Ahmad; supervision, Muhammad Ashraf Shaheen, Muhammad Fayyaz ur Rehman. All authors have carefully read and agreed to the published version of the manuscript.

## Conflicts of interest

All authors have no conflict of interest.

## Supplementary Material

RA-015-D5RA00065C-s001

RA-015-D5RA00065C-s002
