# Supplementary material for: Synthesis, characterization and antidiabetic studies of novel amantadine-derived Schiff base (AHB) and its Zn(ii), Co(ii), Cr(iii) and VO(iv) complexes
Source: RSC Adv. 2025 Jun 4;15(23):18752–65. doi: 10.1039/d5ra00065c (PMC12134986; doi:10.1039/d5ra00065c)
Supplement: RA-015-D5RA00065C-s001 [file RA-015-D5RA00065C-s001.pdf]

Supplementary Information (SI) for RSC Advances.
This journal is © The Royal Society of Chemistry 2025

Supplementary Information

Synthesis, characterization and antidiabetic studies of novel amantadine-derived Schiff base (AHB) and its Zn(II), Co(II), Cr(III) and VO(IV) complexes

Aliya Ajaz [1], Muhammad Ashraf Shaheen [1,2]*, Muhammad Fayyaz ur Rehman [1], Maqsood Ahmad [3], Khurram Shahzad Munawar [1,4], Abu Bakar Siddique [1], Muhammad Ashfaq [5], Nazir Ahmad [6]

[1] *Institute of Chemistry, University of Sargodha, 40100, Pakistan*

[2] *Department of Allied Health Sciences, Superior University, Sargodha-Campus, 40100, Pakistan*

[3] *Institute of Chemistry, The Islamia University of Bahawalpur, Baghdad-ul-Jadeed Campus, 63100, Pakistan*

[4] *Department of Chemistry, University of Mianwali, Mianwali, 42200, Pakistan*

[5] *Department of Physics, University of Sargodha, 42200, Pakistan*

[6] *Department of Chemistry, Government College University Lahore, 54000, Pakistan*

* **Corresponding Authors**: ashraf.shaheen@uos.edu.pk

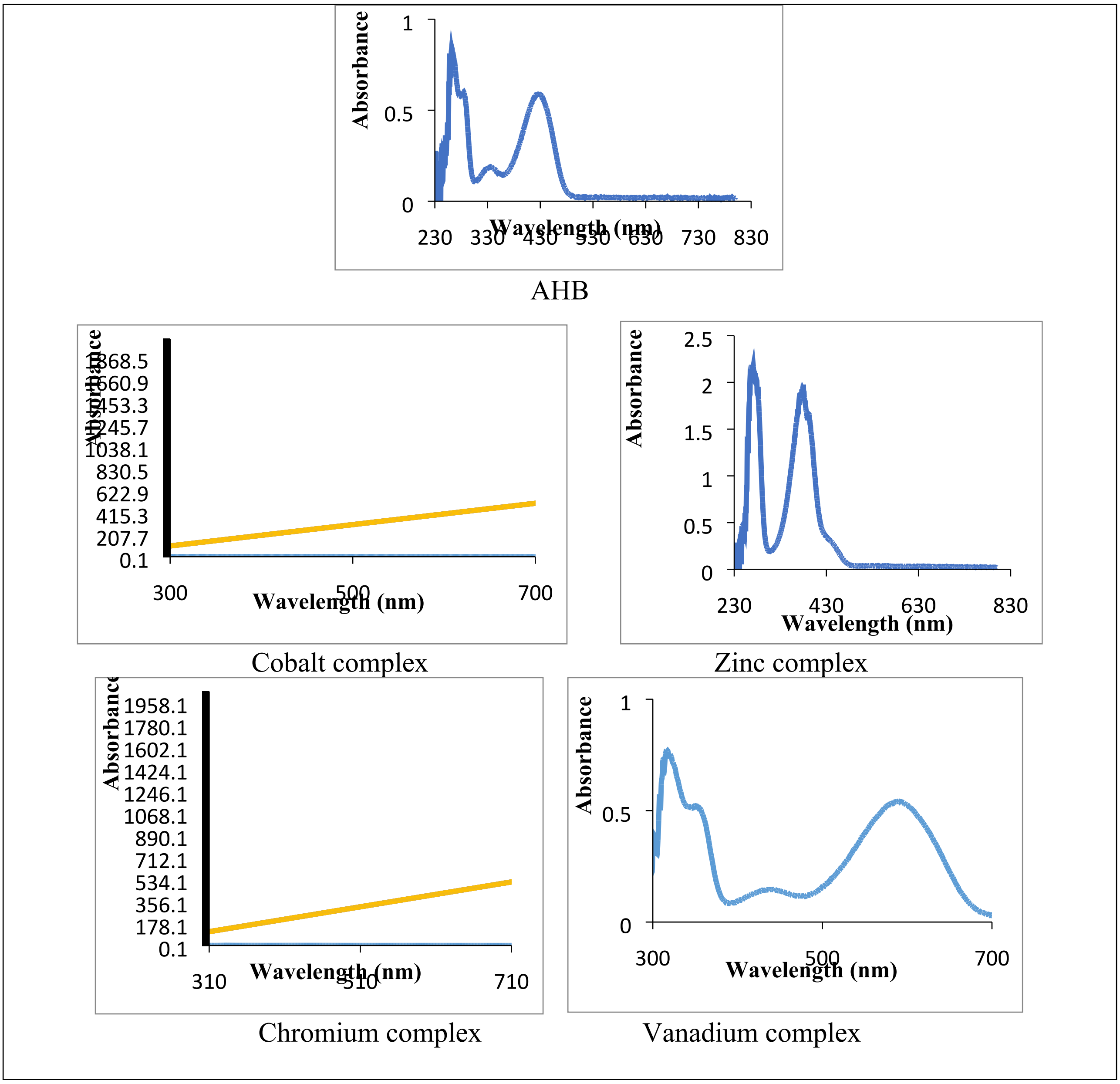

Figure S1. UV-Vis spectra of ligand (AHB) and its metal complexes in DMSO solvent.

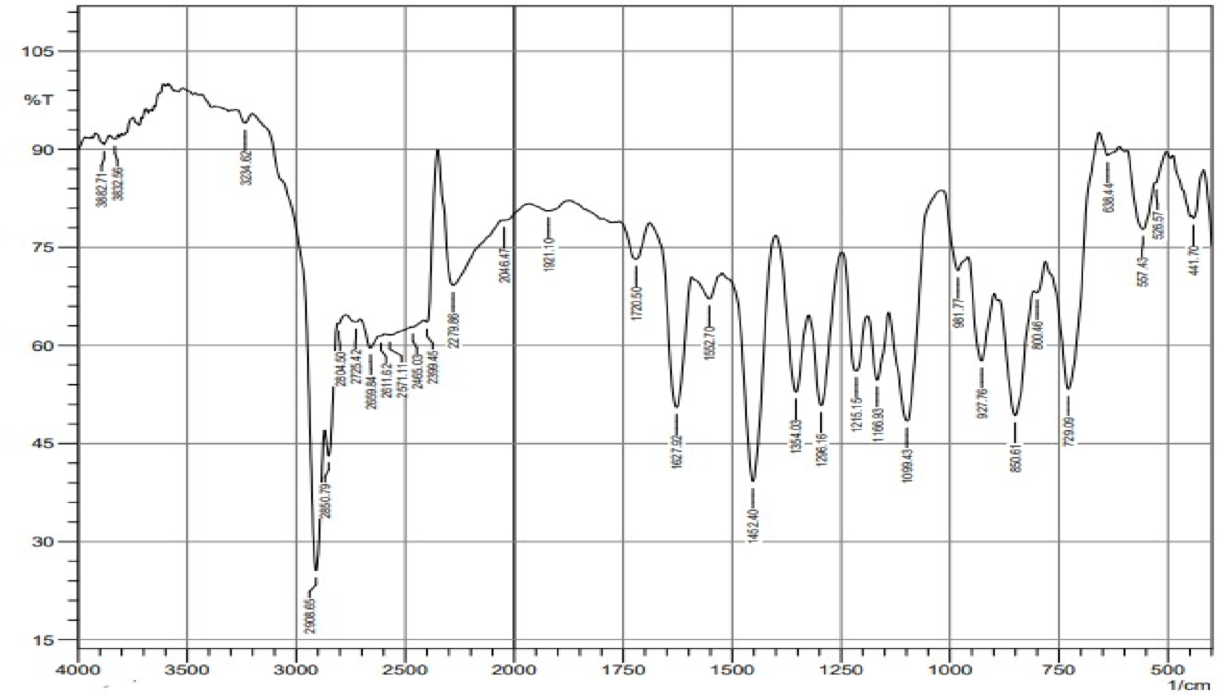

Figure S2. FT-IR spectrum of (*E*)-2-((adamantan-1-ylimino)methyl)-6-bromo-4-chlorophenol (AHB)

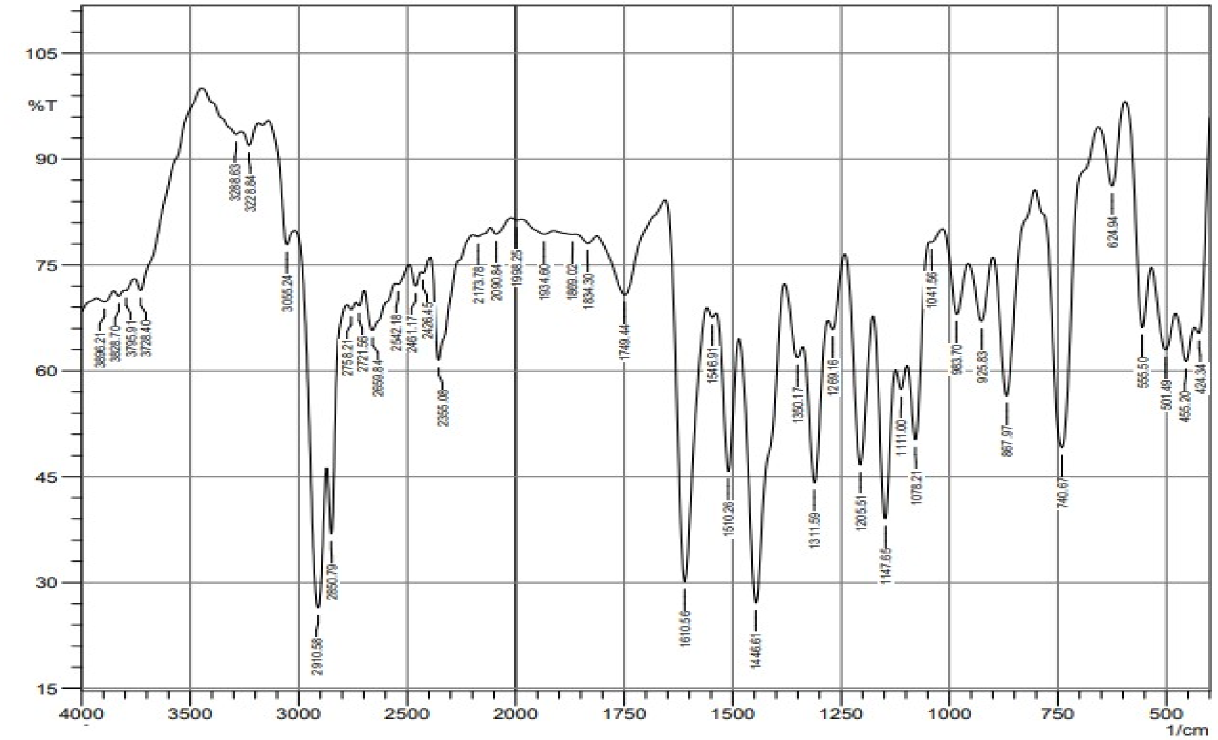

Figure S3. FT-IR spectrum of Zn(II) complex of (*E*)-2-((adamantan-1-ylimino)methyl)-6-bromo-4-chlorophenol (AHB)

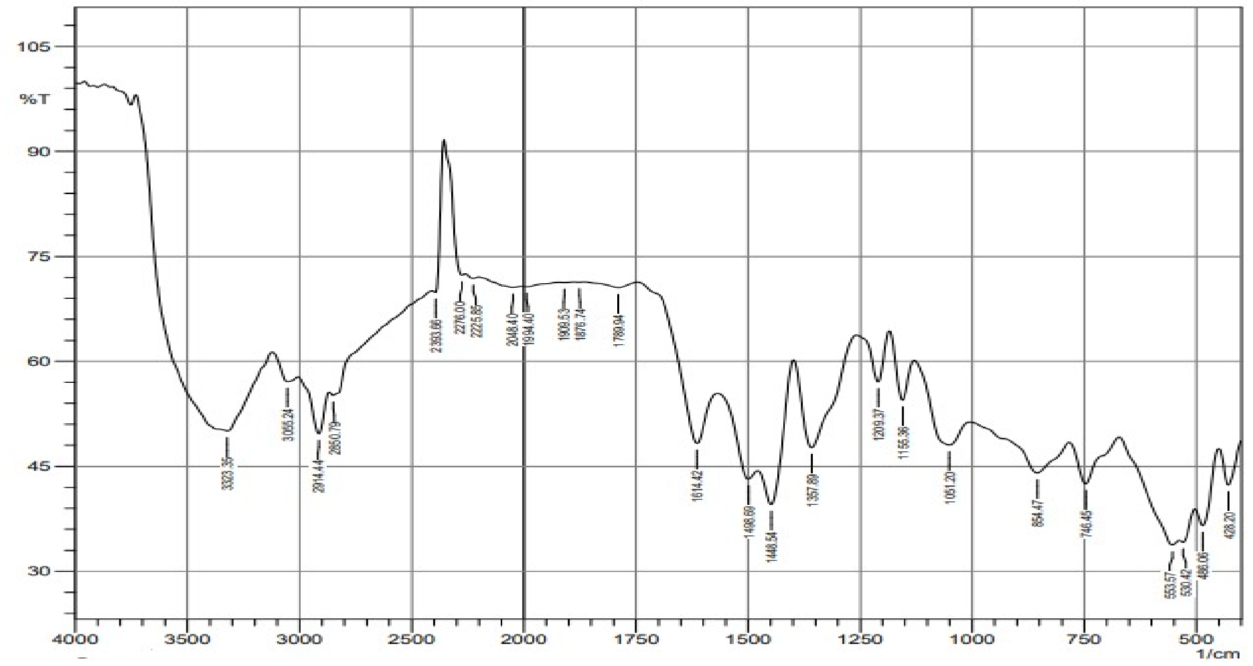

Figure S4. FT-IR spectrum of Cr(III) complex of (*E*)-2-((adamantan-1-ylimino)methyl)-6-bromo-4-chlorophenol (AHB).

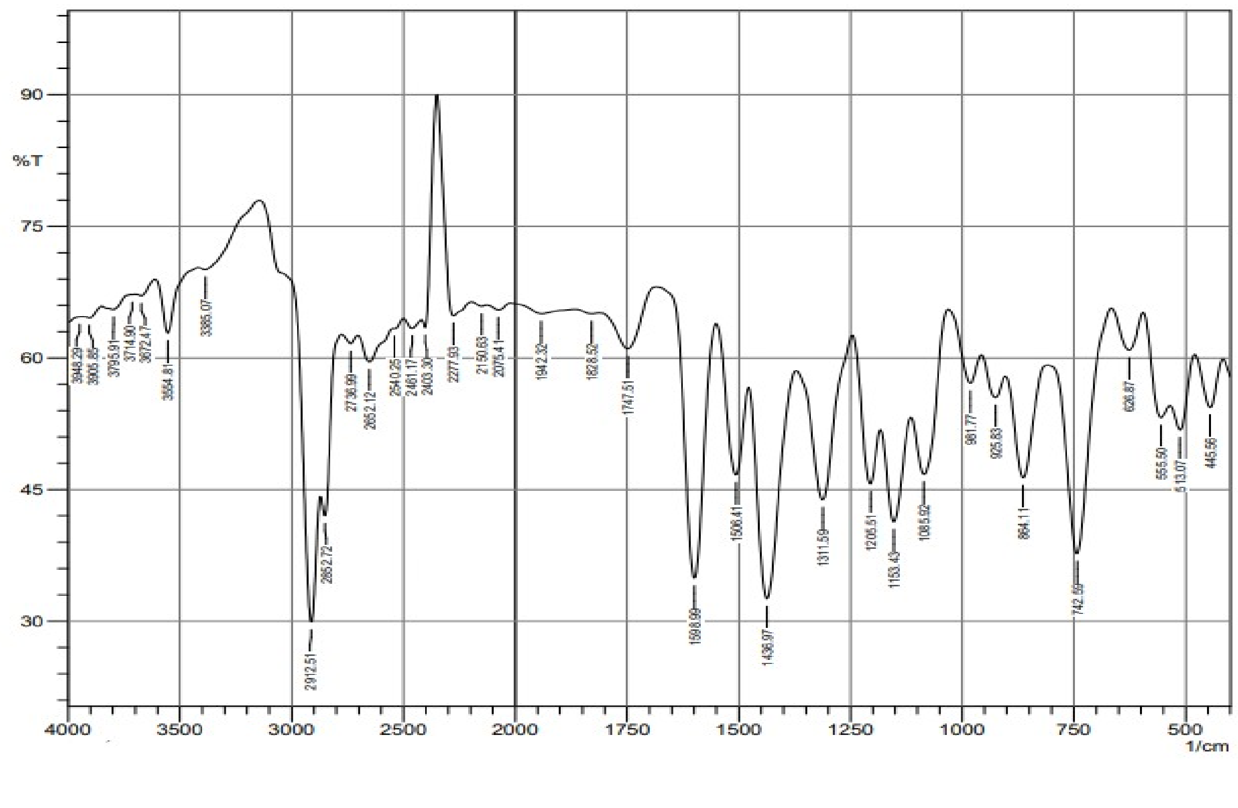

Figure S5. FT-IR spectrum of Co(II) complex of (*E*)-2-((adamantan-1-ylimino)methyl)-6-bromo-4-chlorophenol (AHB)

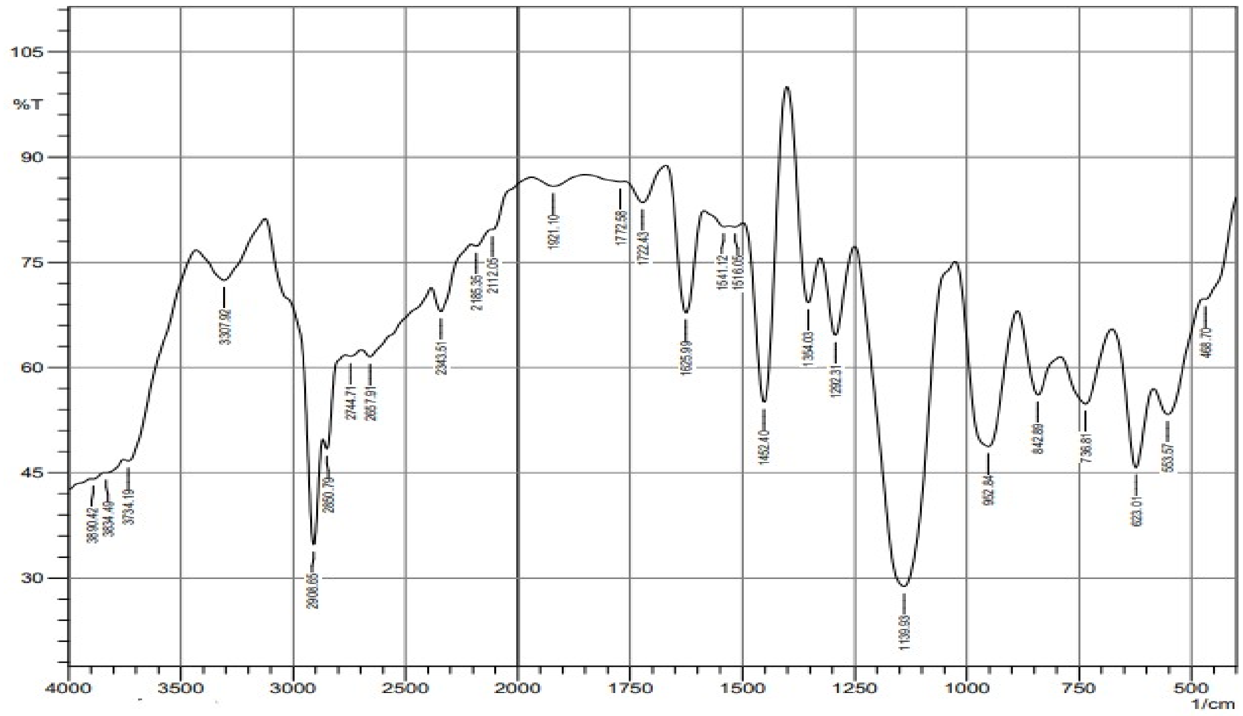

Figure S6. FT-IR spectrum of VO(IV) complex of (*E*)-2-((adamantan-1-ylimino)methyl)-6-bromo-4-chlorophenol (AHB).

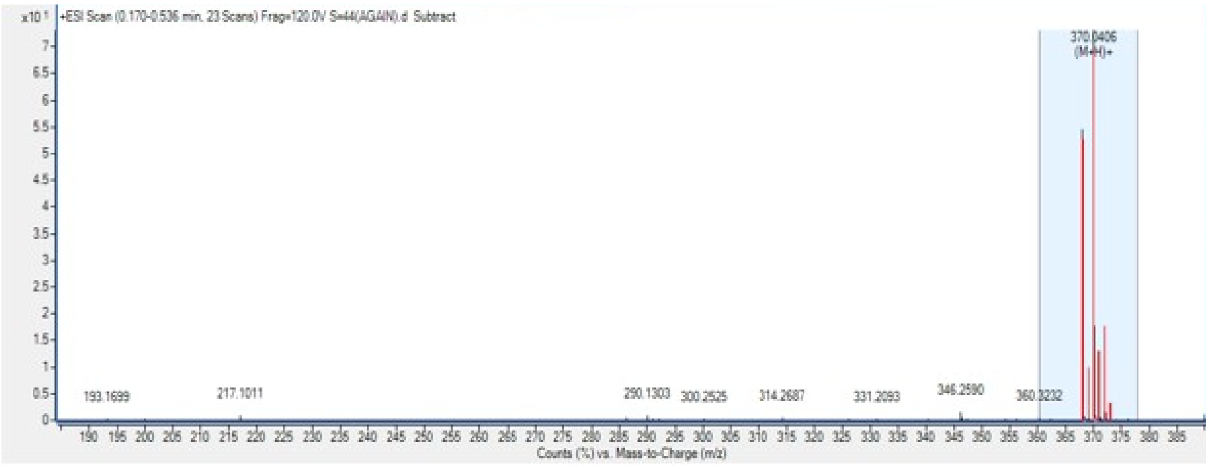

Figure S7. Mass spectrum of (*E*)-2-((adamantan-1-ylimino)methyl)-6-bromo-4-chlorophenol (AHB).

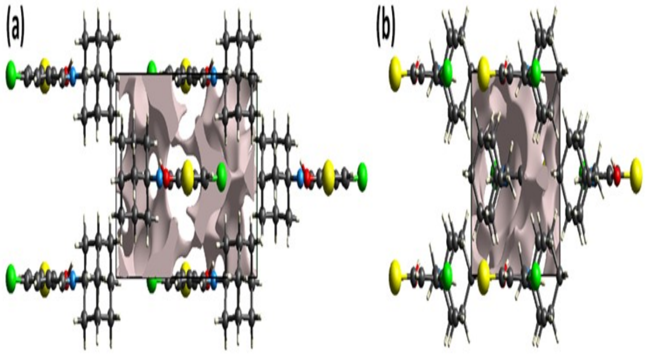

Figure S8. Graphical representation of voids in ligand (AHB) viewed alongside **(a)** *a*-axis and **(b)** *c*-axis.

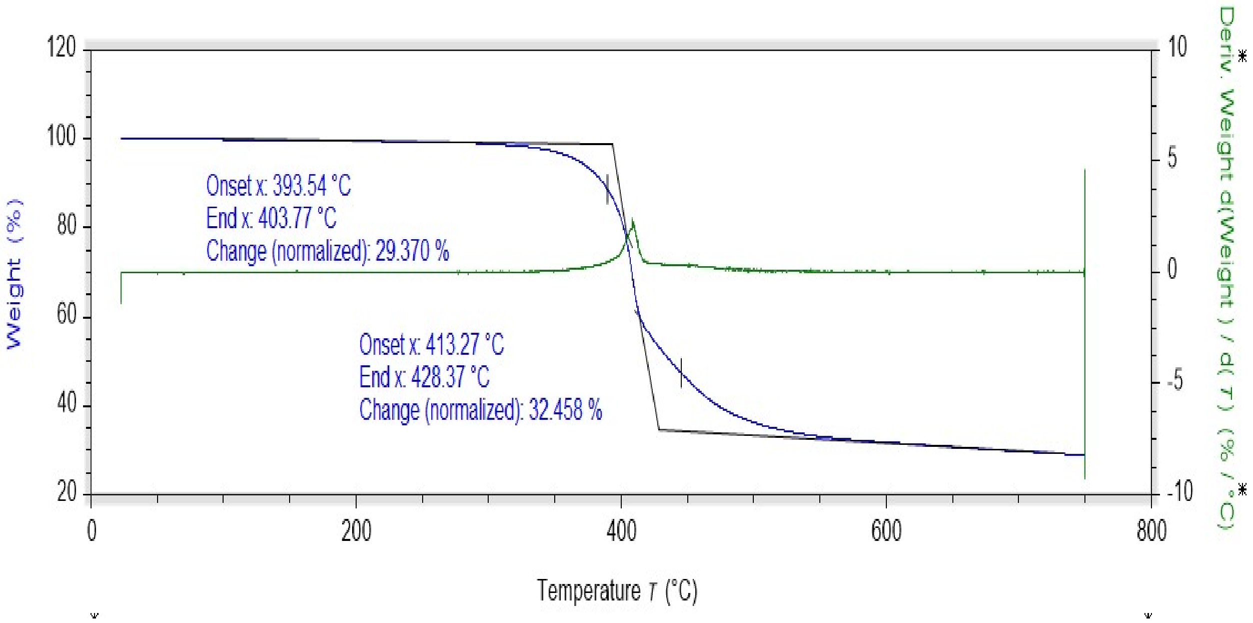

Figure S9. Thermogram Zn(II) complex of (*E*)-2-((adamantan-1-ylimino)methyl)-6-bromo-4-chlorophenol (AHB).

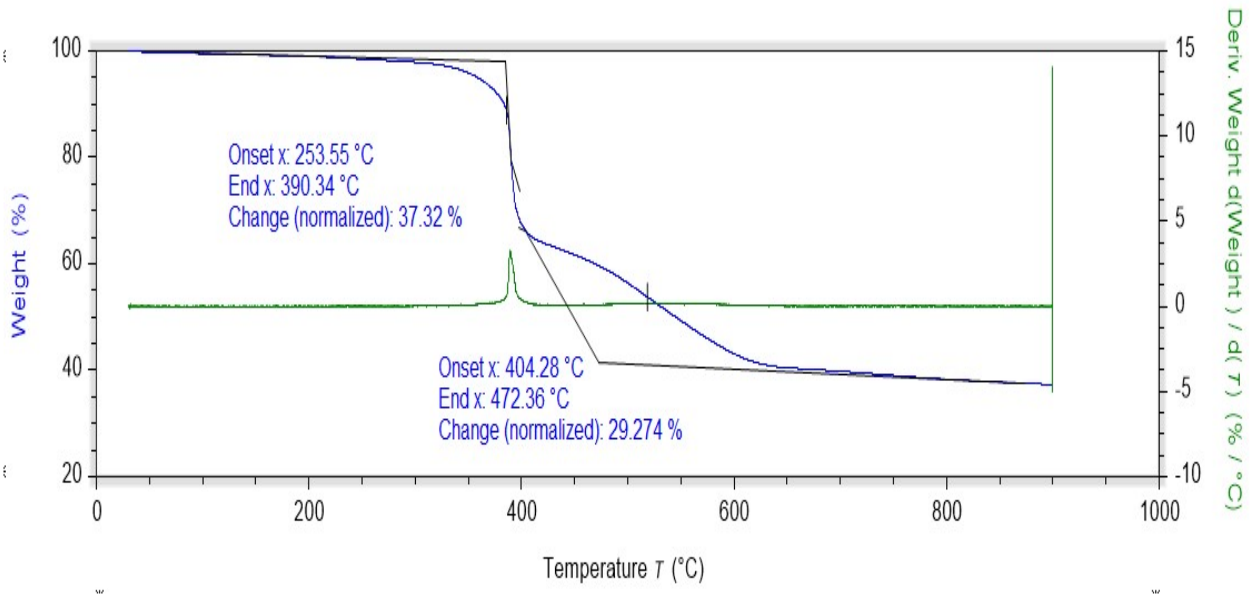

Figure S10. Thermogram Co(II) complex of (*E*)-2-((adamantan-1-ylimino)methyl)-6-bromo-4-chlorophenol (AHB).

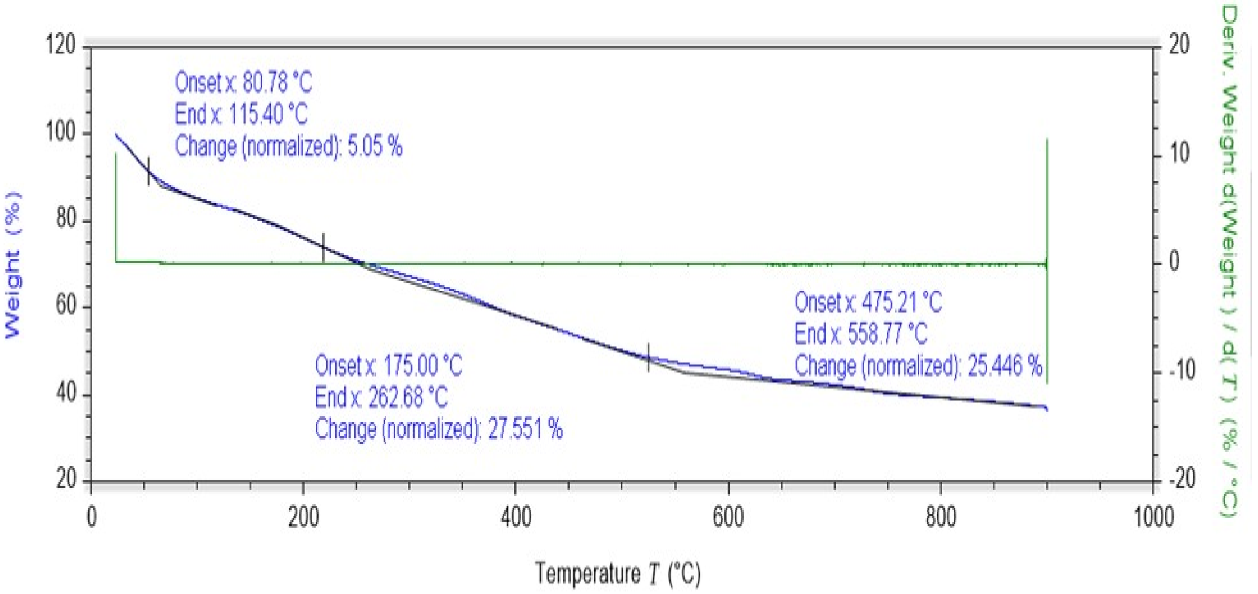

Figure S11. Thermogram Cr(III) complex of (*E*)-2-((adamantan-1-ylimino)methyl)-6-bromo-4-chlorophenol (AHB).

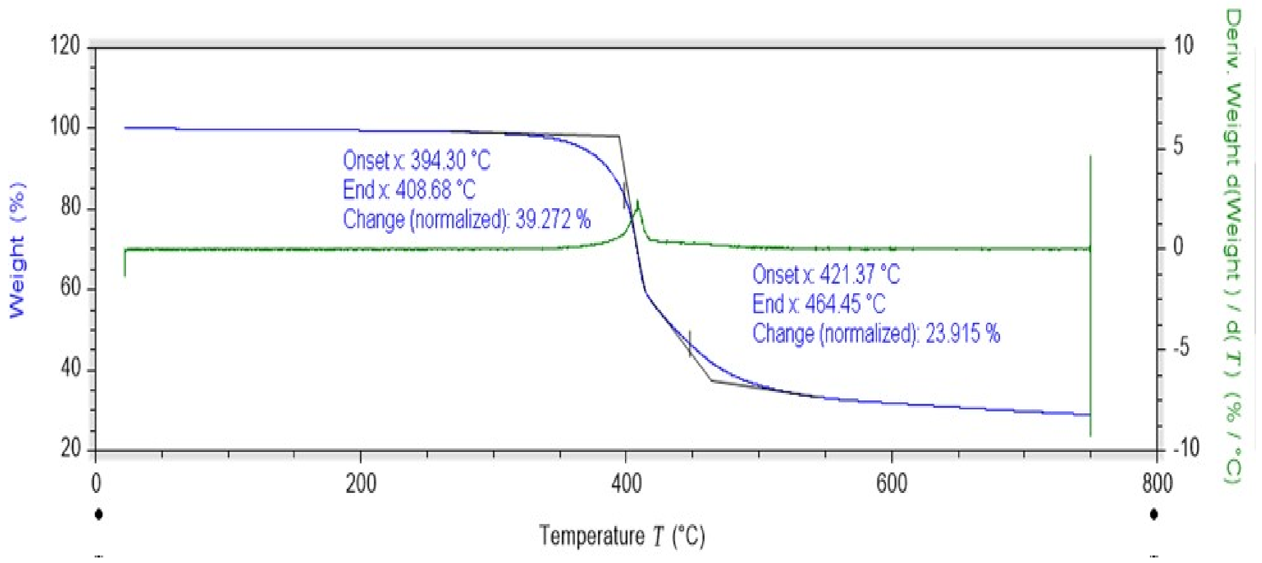

Figure S12. Thermogram oxovandium(IV) complex of (*E*)-2-((adamantan-1-ylimino)methyl)-6-bromo-4-chlorophenol (AHB).

Table S1. Hydrogen-bond geometry (Å,°) for (*E*)-2-((adamantan-2-ylimino)methyl)-6-bromo-4-chlorophenol (AHB).

D*—H···*A	***D*—H**	**H···*A***	***D*···*A***	***<(D*—H···*A)°***
O1—H1···N1	0.82	2.10	2.63 (3)	121
C7—H7···Br1^{i}	0.93	2.97	3.856 (15)	159

Symmetry code: (i) *x*−1, *y*, *z*.

Table S2: Enrichment ratio for the pairs of chemical species in (*E*)-2-((adamantan-1-ylimino)methyl)-6-bromo-4-chlorophenol (AHB).

	Atom	H	C	N	O	Cl	Br
**Contact %**	**H**	48.7	3.2	1	4.7	14.3	16.7
	**C**	3.2	7.1	0.2	1.9		0.2
	**N**	1	0.2				
	**O**	4.7	1.9			1.2	
	**Cl**	14.3			1.2		
	**Br**	16.7	0.2				0.8
**Surface%**		68.65	9.85	0.6	3.9	7.75	9.25
**Random Contacts %**	**Atom**	**H**	**C**	**N**	**O**	**Cl**	**Br**
	**H**	47.13					
	**C**	13.52	0.97				
	**N**	0.82	0.12	0.00			
	**O**	5.35	0.77	0.05	0.15		
	**Cl**	10.64	1.53	0.09	0.60	0.60	
	**Br**	12.70	1.82	0.11	0.72	1.43	0.86
**Enrichment ratio**	**Atom**	**H**	**C**	**N**	**O**	**Cl**	**Br**
	**H**	1.03					
	**C**	0.24	7.32				
	**N**	1.21	1.69	0.00			
	**O**	0.88	2.47	0.00	0.00		
	**Cl**	1.34	0.00	0.00	1.99	0.00	
	**Br**	1.31	0.11	0.00	0.00	0.00	0.93

Table S3: Cartesian coordinates of the atoms used in interaction energy calculations.

Label Symbol x,y,z Occ.

H17A H -3.1473 3.4339 10.7299 1.000
H17B H -3.2976 3.3694 12.4797 1.000
Br1 BR 5.9913 3.3288 6.6440 1.000
C3 C 3.7531 3.3601 5.1146 1.000
H3 H 4.4152 3.4162 4.2594 1.000
N1 N 1.1170 3.3057 9.3254 1.000
C1 C 3.2503 3.3125 7.4894 1.000
C5 C 1.5093 3.2717 5.9228 1.000
H5 H 0.4482 3.1957 5.7204 1.000
C6 C 1.8622 3.3465 7.0260 1.000

O1 O 3.6703 3.3057 8.7004 1.000
H1 H 3.0694 3.8483 9.2579 1.000
C7 C 0.7960 3.2581 8.3165 1.000
H7 H -0.2620 3.1498 8.1120 1.000
C8 C 0.1357 3.3329 10.5027 1.000
C14 C -0.7784 4.5096 10.5202 1.000
H14A H -1.4096 4.4731 9.6408 1.000
H14B H -0.1844 5.4126 10.4517 1.000
C15 C -1.6754 4.5912 11.7864 1.000
H15 H -2.2522 5.5078 11.7825 1.000
C17 C -2.5919 3.3873 11.6584 1.000
C16 C -0.7856 4.5436 12.9313 1.000
H16B H -0.0942 5.3757 12.8828 1.000
C12 C -1.7223 2.1018 11.6853 1.000
H12 H -2.3844 1.2447 11.6794 1.000
C11 C -0.9130 2.0609 12.9583 1.000
H11B H -0.3339 1.1464 12.9901 1.000
C10 C -0.0006 3.2513 12.9798 1.000
C13 C -0.7574 1.9929 10.4259 1.000
H13A H -1.3262 1.9550 9.5051 1.000
H13B H -0.1369 1.1076 10.4900 1.000
C9 C 0.8824 3.4281 11.7338 1.000
H9A H 1.3699 4.3940 11.7804 1.000
H9B H 1.6525 2.6666 11.7383 1.000
C2 C 4.2396 3.3601 6.3539 1.000
C4 C 2.3267 3.2785 4.9368 1.000
Cl1 CL 1.6075 3.3533 3.4106 1.000
H16A H -1.3656 4.6432 13.8406 1.000
H11A H -1.5771 2.0811 13.8135 1.000
H10 H 0.6159 3.2233 13.8698 1.000

Unselected Atoms
560 atoms, Cartesian coordinates
Label Symbol x y z Occ.

H5 H -3.4428 -0.2052 7.7498 1.000
H11A H -4.4122 -1.3198 13.1268 1.000
Cl1 CL -4.6022 -0.0476 10.0595 1.000
Br1 BR -2.6777 3.3288 6.6440 1.000
H5 H -3.4428 6.5966 7.7498 1.000
O1 O -4.9987 3.3057 8.7004 1.000
H1 H -5.5996 3.8483 9.2579 1.000
H14A H -1.5851 1.0722 3.8293 1.000
H15 H -0.7424 2.1069 1.6877 1.000
H16A H -4.6236 1.2423 13.0997 1.000

H12 H -0.6102 4.6456 1.7907 1.000
H11A H -4.4122 5.4820 13.1268 1.000
H13A H -1.6685 5.3559 3.9651 1.000
Cl1 CL -4.6022 6.7542 10.0595 1.000
C15 C -4.3139 1.1903 15.1539 1.000
H15 H -3.7371 2.1069 15.1578 1.000
C16 C -5.2037 1.1427 14.0089 1.000
H16B H -5.8951 1.9748 14.0574 1.000
C12 C -4.2670 5.5027 15.2549 1.000
H12 H -3.6049 4.6456 15.2609 1.000
C11 C -5.0763 5.4618 13.9820 1.000
H11B H -5.6554 4.5473 13.9502 1.000
H16A H -4.6236 8.0441 13.0997 1.000
Br1 BR -0.3169 -0.0721 6.8261 1.000
C3 C 1.9212 -0.0408 8.3555 1.000
N1 N 4.5574 -0.0952 4.1448 1.000
C1 C 2.4241 -0.0884 5.9807 1.000
C5 C 4.1650 -0.1292 7.5473 1.000
H5 H 5.2262 -0.2052 7.7498 1.000
C6 C 3.8121 -0.0544 6.4441 1.000
O1 O 2.0041 -0.0952 4.7698 1.000
C7 C 4.8783 -0.1428 5.1537 1.000
C14 C -0.7784 -2.2922 10.5202 1.000
H14A H -1.4096 -2.3287 9.6408 1.000
H14B H -0.1844 -1.3892 10.4517 1.000
C15 C -1.6754 -2.2106 11.7864 1.000
H15 H -2.2522 -1.2940 11.7825 1.000
C16 C -0.7856 -2.2582 12.9313 1.000
H16B H -0.0942 -1.4261 12.8828 1.000
H10 H 2.0638 -0.1776 13.0705 1.000
C2 C 1.4347 -0.0408 7.1163 1.000
C4 C 3.3476 -0.1224 8.5333 1.000
Cl1 CL 4.0668 -0.0476 10.0595 1.000
C17 C -3.3974 -0.0136 15.2819 1.000
H17B H -2.6917 -0.0315 14.4606 1.000
H16A H -1.3656 -2.1586 13.8406 1.000
C10 C 2.6803 -0.1496 13.9604 1.000
H9B H 1.0272 -0.7343 15.2020 1.000
Br1 BR -0.3169 6.7297 6.8261 1.000
C3 C 1.9212 6.7610 8.3555 1.000
H3 H 1.2592 0.0153 9.2107 1.000
N1 N 4.5574 6.7066 4.1448 1.000
C1 C 2.4241 6.7134 5.9807 1.000
C5 C 4.1650 6.6726 7.5473 1.000
H5 H 5.2262 6.5966 7.7498 1.000
C6 C 3.8121 6.7474 6.4441 1.000

O1 O 2.0041 6.7066 4.7698 1.000
H1 H 2.6050 0.4474 4.2122 1.000
C7 C 4.8783 6.6590 5.1537 1.000
H7 H 5.9364 6.5507 5.3582 1.000
C8 C 5.5386 6.7338 2.9675 1.000
C14 C 6.4528 1.1087 2.9500 1.000
H14A H 7.0839 1.0722 3.8293 1.000
H14B H 5.8588 2.0117 3.0184 1.000
H17A H 5.5217 3.4339 10.7299 1.000
H17A H 0.1526 0.0330 2.7402 1.000
H16A H 1.6290 4.6432 0.3704 1.000
H16A H 4.0454 1.2423 13.0997 1.000
H11A H 1.4175 2.0811 0.3434 1.000
H11A H 4.2568 5.4820 13.1268 1.000
H10 H 3.6106 3.2233 0.3997 1.000
H10 H 2.0638 6.6242 13.0705 1.000
C13 C 6.4318 5.3938 3.0443 1.000
H13A H 7.0005 5.3559 3.9651 1.000
H13B H 5.8113 4.5085 2.9801 1.000
H9A H 4.3045 0.9931 1.6897 1.000
H9B H 4.0219 6.0675 1.7319 1.000
C2 C 1.4347 6.7610 7.1163 1.000
C4 C 3.3476 6.6794 8.5333 1.000
Cl1 CL 4.0668 6.7542 10.0595 1.000
H14B H 2.8642 2.0117 16.4885 1.000
C17 C -3.3974 6.7882 15.2819 1.000
H17A H -2.8420 0.0330 16.2104 1.000
H17B H -2.6917 6.7703 14.4606 1.000
C16 C 3.4653 1.1427 14.0089 1.000
H16B H 2.7739 1.9748 14.0574 1.000
C11 C 3.5927 5.4618 13.9820 1.000
H11B H 3.0136 4.5473 13.9502 1.000
C10 C 2.6803 6.6522 13.9604 1.000
H13B H 2.8167 4.5085 16.4503 1.000
C9 C 1.7973 0.0272 15.2064 1.000
H9A H 1.3098 0.9931 15.1598 1.000
H9B H 1.0272 6.0675 15.2020 1.000
Cl1 CL -1.3871 3.3533 16.8808 1.000
H3 H 1.2592 6.8171 9.2107 1.000
C12 C -1.7223 8.9036 11.6853 1.000
H12 H -2.3844 8.0465 11.6794 1.000
C11 C -0.9130 8.8627 12.9583 1.000
H11B H -0.3339 7.9482 12.9901 1.000
C13 C -0.7574 8.7947 10.4259 1.000
H13A H -1.3262 8.7568 9.5051 1.000
H13B H -0.1369 7.9094 10.4900 1.000

H17A H -2.8420 6.8348 16.2104 1.000
C9 C 1.7973 6.8290 15.2064 1.000
H9A H 1.3098 7.7949 15.1598 1.000
H5 H 9.1172 3.1957 5.7204 1.000
H7 H 8.4070 3.1498 8.1120 1.000
H14A H 7.2594 4.4731 9.6408 1.000
H13A H 7.3428 1.9550 9.5051 1.000
C5 C -4.5040 -0.1292 7.5473 1.000
C11 C -5.0763 -1.3400 13.9820 1.000
C4 C -5.3214 -0.1224 8.5333 1.000
C2 C -4.4294 3.3601 6.3539 1.000
C5 C -4.5040 6.6726 7.5473 1.000
C1 C -5.4187 3.3125 7.4894 1.000
C14 C -2.2162 1.1087 2.9500 1.000
C15 C -1.3193 1.1903 1.6838 1.000
C12 C -1.2724 5.5027 1.7848 1.000
C13 C -2.2372 5.3938 3.0443 1.000
C4 C -5.3214 6.6794 8.5333 1.000
C14 C -5.2109 1.1087 16.4201 1.000
C10 C -5.9887 -0.1496 13.9604 1.000
C13 C -5.2319 5.3938 16.5144 1.000
C10 C -5.9887 6.6522 13.9604 1.000
C16 C -5.2037 7.9445 14.0089 1.000
C8 C 5.5386 -0.0680 2.9675 1.000
H7 H 5.9364 -0.2511 5.3582 1.000
C8 C 0.1357 -3.4689 10.5027 1.000
C17 C -2.5919 -3.4145 11.6584 1.000
C10 C -0.0006 -3.5505 12.9798 1.000
C12 C -4.2670 -1.2991 15.2549 1.000
C11 C 3.5927 -1.3400 13.9820 1.000
H1 H 2.6050 7.2492 4.2122 1.000
C14 C 6.4528 7.9105 2.9500 1.000
C9 C 4.7920 6.8290 1.7363 1.000
C15 C 7.3497 1.1903 1.6838 1.000
C17 C 6.0771 3.3873 11.6584 1.000
C17 C -0.4028 -0.0136 1.8117 1.000
C16 C 2.2090 4.5436 -0.5388 1.000
C11 C 2.0817 2.0609 -0.5119 1.000
C10 C 2.9940 3.2513 -0.4903 1.000
C12 C 7.3966 5.5027 1.7848 1.000
C9 C 4.7920 0.0272 1.7363 1.000
C14 C 3.4581 1.1087 16.4201 1.000
C15 C -4.3139 7.9921 15.1539 1.000
C15 C 4.3551 1.1903 15.1539 1.000
C12 C 4.4020 5.5027 15.2549 1.000
C16 C 3.4653 7.9445 14.0089 1.000

C13 C 3.4371 5.3938 16.5144 1.000
C8 C 2.5440 -0.0680 16.4376 1.000
C4 C -0.6679 3.2785 18.4069 1.000
C17 C -2.5919 10.1891 11.6584 1.000
H11A H -1.5771 8.8829 13.8135 1.000
C10 C -0.0006 10.0531 12.9798 1.000
C8 C 0.1357 10.1347 10.5027 1.000
C8 C 2.5440 6.7338 16.4376 1.000
C5 C 10.1783 3.2717 5.9228 1.000
C7 C 9.4650 3.2581 8.3165 1.000
C14 C 7.8906 4.5096 10.5202 1.000
C13 C 7.9116 1.9929 10.4259 1.000
C6 C -4.8569 -0.0544 6.4441 1.000
H11B H -5.6554 -2.2545 13.9502 1.000
C3 C -6.7478 -0.0408 8.3555 1.000
C3 C -4.9159 3.3601 5.1146 1.000
C6 C -4.8569 6.7474 6.4441 1.000
C6 C -6.8068 3.3465 7.0260 1.000
C8 C -3.1304 -0.0680 2.9675 1.000
H14B H -2.8102 2.0117 3.0184 1.000
C16 C -2.2090 1.1427 0.5388 1.000
C17 C -0.4028 6.7882 1.8117 1.000
C11 C -2.0817 5.4618 0.5119 1.000
C8 C -3.1304 6.7338 2.9675 1.000
H13B H -2.8577 4.5085 2.9801 1.000
C3 C -6.7478 6.7610 8.3555 1.000
C8 C -6.1250 -0.0680 16.4376 1.000
H14A H -4.5797 1.0722 17.2994 1.000
H14B H -5.8048 2.0117 16.4885 1.000
H10 H -6.6052 -0.1776 13.0705 1.000
C9 C -6.8717 0.0272 15.2064 1.000
C8 C -6.1250 6.7338 16.4376 1.000
H13A H -4.6631 5.3559 17.4352 1.000
H13B H -5.8523 4.5085 16.4503 1.000
H10 H -6.6052 6.6242 13.0705 1.000
C9 C -6.8717 6.8290 15.2064 1.000
H16B H -5.8951 8.7766 14.0574 1.000
C13 C 6.4318 -1.4080 3.0443 1.000
N1 N 1.1170 -3.4961 9.3254 1.000
C13 C -0.7574 -4.8089 10.4259 1.000
C9 C 0.8824 -3.3737 11.7338 1.000
H17A H -3.1473 -3.3679 10.7299 1.000
H17B H -3.2976 -3.4324 12.4797 1.000
C12 C -1.7223 -4.7000 11.6853 1.000
C11 C -0.9130 -4.7409 12.9583 1.000
H10 H 0.6159 -3.5785 13.8698 1.000

H12 H -3.6049 -2.1562 15.2609 1.000
C13 C -5.2319 -1.4080 16.5144 1.000
C12 C 4.4020 -1.2991 15.2549 1.000
H11A H 4.2568 -1.3198 13.1268 1.000
H11B H 3.0136 -2.2545 13.9502 1.000
H14A H 7.0839 7.8740 3.8293 1.000
H14B H 5.8588 8.8135 3.0184 1.000
C15 C 7.3497 7.9921 1.6838 1.000
C10 C 5.6750 6.6522 0.4903 1.000
H9A H 4.3045 7.7949 1.6897 1.000
H15 H 7.9266 2.1069 1.6877 1.000
C17 C 8.2662 -0.0136 1.8117 1.000
C16 C 6.4600 1.1427 0.5388 1.000
C15 C 6.9936 4.5912 11.7864 1.000
H17B H 5.3714 3.3694 12.4797 1.000
C12 C 6.9467 2.1018 11.6853 1.000
H17B H 0.3030 -0.0315 0.9905 1.000
C12 C -1.2724 -1.2991 1.7848 1.000
C15 C 1.3193 4.5912 -1.6838 1.000
H16B H 2.9005 5.3757 -0.5873 1.000
C12 C 1.2724 2.1018 -1.7848 1.000
H11B H 2.6608 1.1464 -0.4801 1.000
C9 C 3.8770 3.4281 -1.7363 1.000
C17 C 8.2662 6.7882 1.8117 1.000
H12 H 8.0588 4.6456 1.7907 1.000
C11 C 6.5873 5.4618 0.5119 1.000
C10 C 5.6750 -0.1496 0.4903 1.000
H9B H 4.0219 -0.7343 1.7319 1.000
H14A H 4.0893 1.0722 17.2994 1.000
C14 C -5.2109 7.9105 16.4201 1.000
H15 H -3.7371 8.9087 15.1578 1.000
H15 H 4.9319 2.1069 15.1578 1.000
C17 C 5.2716 -0.0136 15.2819 1.000
C17 C 5.2716 6.7882 15.2819 1.000
H12 H 5.0641 4.6456 15.2609 1.000
C15 C 4.3551 7.9921 15.1539 1.000
H16A H 4.0454 8.0441 13.0997 1.000
H16B H 2.7739 8.7766 14.0574 1.000
H13A H 4.0059 5.3559 17.4352 1.000
N1 N 1.5627 -0.0952 17.6149 1.000
C13 C 3.4371 -1.4080 16.5144 1.000
C3 C 0.7585 3.3601 18.5847 1.000
C5 C -1.4853 3.2717 19.3930 1.000
C15 C -1.6754 11.3930 11.7864 1.000
H17A H -3.1473 10.2357 10.7299 1.000
H17B H -3.2976 10.1712 12.4797 1.000

C16 C -0.7856 11.3454 12.9313 1.000
H10 H 0.6159 10.0251 13.8698 1.000
C9 C 0.8824 10.2299 11.7338 1.000
N1 N 1.1170 10.1075 9.3254 1.000
C14 C -0.7784 11.3114 10.5202 1.000
N1 N 1.5627 6.7066 17.6149 1.000
C14 C 3.4581 7.9105 16.4201 1.000
C6 C 10.5312 3.3465 7.0260 1.000
C4 C 10.9957 3.2785 4.9368 1.000
N1 N 9.7860 3.3057 9.3254 1.000
C8 C 8.8047 3.3329 10.5027 1.000
H14B H 8.4846 5.4126 10.4517 1.000
H13B H 8.5321 1.1076 10.4900 1.000
C1 C -6.2449 -0.0884 5.9807 1.000
C7 C -3.7907 -0.1428 5.1537 1.000
H3 H -7.4098 0.0153 9.2107 1.000
C2 C -7.2343 -0.0408 7.1163 1.000
H3 H -4.2538 3.4162 4.2594 1.000
C4 C -6.3423 3.2785 4.9368 1.000
C1 C -6.2449 6.7134 5.9807 1.000
C7 C -3.7907 6.6590 5.1537 1.000
C5 C -7.1597 3.2717 5.9228 1.000
C7 C -7.8730 3.2581 8.3165 1.000
N1 N -4.1116 -0.0952 4.1448 1.000
C13 C -2.2372 -1.4080 3.0443 1.000
C9 C -3.8770 0.0272 1.7363 1.000
H16A H -1.6290 1.2423 -0.3704 1.000
H16B H -2.9005 1.9748 0.5873 1.000
C10 C -2.9940 -0.1496 0.4903 1.000
C15 C -1.3193 7.9921 1.6838 1.000
H17A H 0.1526 6.8348 2.7402 1.000
H17B H 0.3030 6.7703 0.9905 1.000
H11A H -1.4175 5.4820 -0.3434 1.000
H11B H -2.6608 4.5473 0.4801 1.000
C10 C -2.9940 6.6522 0.4903 1.000
N1 N -4.1116 6.7066 4.1448 1.000
C14 C -2.2162 7.9105 2.9500 1.000
C9 C -3.8770 6.8290 1.7363 1.000
H3 H -7.4098 6.8171 9.2107 1.000
C2 C -7.2343 6.7610 7.1163 1.000
N1 N -7.1063 -0.0952 17.6149 1.000
H9A H -7.3592 0.9931 15.1598 1.000
H9B H -7.6418 -0.7343 15.2020 1.000
N1 N -7.1063 6.7066 17.6149 1.000
H9A H -7.3592 7.7949 15.1598 1.000
H9B H -7.6418 6.0675 15.2020 1.000

C12 C 7.3966 -1.2991 1.7848 1.000
H13A H 7.0005 -1.4459 3.9651 1.000
H13B H 5.8113 -2.2933 2.9801 1.000
C7 C 0.7960 -3.5437 8.3165 1.000
H13A H -1.3262 -4.8468 9.5051 1.000
H13B H -0.1369 -5.6942 10.4900 1.000
H9A H 1.3699 -2.4078 11.7804 1.000
H9B H 1.6525 -4.1352 11.7383 1.000
H12 H -2.3844 -5.5571 11.6794 1.000
H11A H -1.5771 -4.7207 13.8135 1.000
H11B H -0.3339 -5.6554 12.9901 1.000
H13A H -4.6631 -1.4459 17.4352 1.000
H13B H -5.8523 -2.2933 16.4503 1.000
H12 H 5.0641 -2.1562 15.2609 1.000
H15 H 7.9266 8.9087 1.6877 1.000
C16 C 6.4600 7.9445 0.5388 1.000
H10 H 5.0584 6.6242 -0.3997 1.000
H17A H 8.8216 0.0330 2.7402 1.000
H17B H 8.9720 -0.0315 0.9905 1.000
H16A H 7.0400 1.2423 -0.3704 1.000
H16B H 5.7685 1.9748 0.5873 1.000
H15 H 6.4168 5.5078 11.7825 1.000
C16 C 7.8834 4.5436 12.9313 1.000
H12 H 6.2846 1.2447 11.6794 1.000
C11 C 7.7560 2.0609 12.9583 1.000
H12 H -0.6102 -2.1562 1.7907 1.000
C11 C -2.0817 -1.3400 0.5119 1.000
C14 C 2.2162 4.5096 -2.9500 1.000
H15 H 0.7424 5.5078 -1.6877 1.000
C17 C 0.4028 3.3873 -1.8117 1.000
H12 H 0.6102 1.2447 -1.7907 1.000
C13 C 2.2372 1.9929 -3.0443 1.000
C8 C 3.1304 3.3329 -2.9675 1.000
H9A H 4.3645 4.3940 -1.6897 1.000
H9B H 4.6471 2.6666 -1.7319 1.000
H17A H 8.8216 6.8348 2.7402 1.000
H17B H 8.9720 6.7703 0.9905 1.000
H11A H 7.2515 5.4820 -0.3434 1.000
H11B H 6.0082 4.5473 0.4801 1.000
C11 C 6.5873 -1.3400 0.5119 1.000
H10 H 5.0584 -0.1776 -0.3997 1.000
H14A H -4.5797 7.8740 17.2994 1.000
H14B H -5.8048 8.8135 16.4885 1.000
H17A H 5.8270 0.0330 16.2104 1.000
H17B H 5.9773 -0.0315 14.4606 1.000
H17A H 5.8270 6.8348 16.2104 1.000

H17B H 5.9773 6.7703 14.4606 1.000
H15 H 4.9319 8.9087 15.1578 1.000
C7 C 1.8837 -0.1428 18.6238 1.000
H13A H 4.0059 -1.4459 17.4352 1.000
H13B H 2.8167 -2.2933 16.4503 1.000
H3 H 1.4205 3.4162 17.7295 1.000
C2 C 1.2450 3.3601 19.8240 1.000
H5 H -2.5464 3.1957 19.1905 1.000
C6 C -1.1324 3.3465 20.4962 1.000
H15 H -2.2522 12.3096 11.7825 1.000
H16A H -1.3656 11.4450 13.8406 1.000
H16B H -0.0942 12.1775 12.8828 1.000
H9A H 1.3699 11.1958 11.7804 1.000
H9B H 1.6525 9.4684 11.7383 1.000
C7 C 0.7960 10.0599 8.3165 1.000
H14A H -1.4096 11.2749 9.6408 1.000
H14B H -0.1844 12.2144 10.4517 1.000
C7 C 1.8837 6.6590 18.6238 1.000
H14A H 4.0893 7.8740 17.2994 1.000
H14B H 2.8642 8.8135 16.4885 1.000
C1 C 11.9193 3.3125 7.4894 1.000
C3 C 12.4221 3.3601 5.1146 1.000
Cl1 CL 10.2765 3.3533 3.4106 1.000
C9 C 9.5514 3.4281 11.7338 1.000
O1 O -6.6649 -0.0952 4.7698 1.000
H7 H -2.7326 -0.2511 5.3582 1.000
Br1 BR -8.9859 -0.0721 6.8261 1.000
Cl1 CL -7.0615 3.3533 3.4106 1.000
O1 O -6.6649 6.7066 4.7698 1.000
H7 H -2.7326 6.5507 5.3582 1.000
H5 H -8.2208 3.1957 5.7204 1.000
N1 N -7.5520 3.3057 9.3254 1.000
H7 H -8.9310 3.1498 8.1120 1.000
H13A H -1.6685 -1.4459 3.9651 1.000
H13B H -2.8577 -2.2933 2.9801 1.000
H9A H -4.3645 0.9931 1.6897 1.000
H9B H -4.6471 -0.7343 1.7319 1.000
H10 H -3.6106 -0.1776 -0.3997 1.000
H15 H -0.7424 8.9087 1.6877 1.000
C16 C -2.2090 7.9445 0.5388 1.000
H10 H -3.6106 6.6242 -0.3997 1.000
H14A H -1.5851 7.8740 3.8293 1.000
H14B H -2.8102 8.8135 3.0184 1.000
H9A H -4.3645 7.7949 1.6897 1.000
H9B H -4.6471 6.0675 1.7319 1.000
Br1 BR -8.9859 6.7297 6.8261 1.000

C7 C -6.7853 -0.1428 18.6238 1.000
C7 C -6.7853 6.6590 18.6238 1.000
H12 H 8.0588 -2.1562 1.7907 1.000
C6 C 1.8622 -3.4553 7.0260 1.000
H7 H -0.2620 -3.6520 8.1120 1.000
H16A H 7.0400 8.0441 -0.3704 1.000
H16B H 5.7685 8.7766 0.5873 1.000
H16A H 7.3034 4.6432 13.8406 1.000
H16B H 8.5748 5.3757 12.8828 1.000
C10 C 8.6684 3.2513 12.9798 1.000
H11A H 7.0919 2.0811 13.8135 1.000
H11B H 8.3351 1.1464 12.9901 1.000
H11A H -1.4175 -1.3198 -0.3434 1.000
H11B H -2.6608 -2.2545 0.4801 1.000
H14A H 1.5851 4.4731 -3.8293 1.000
H14B H 2.8102 5.4126 -3.0184 1.000
H17A H -0.1526 3.4339 -2.7402 1.000
H17B H -0.3030 3.3694 -0.9905 1.000
H13A H 1.6685 1.9550 -3.9651 1.000
H13B H 2.8577 1.1076 -2.9801 1.000
N1 N 4.1116 3.3057 -4.1448 1.000
H11A H 7.2515 -1.3198 -0.3434 1.000
H11B H 6.0082 -2.2545 0.4801 1.000
C6 C 0.8175 -0.0544 19.9142 1.000
H7 H 2.9417 -0.2511 18.8283 1.000
Br1 BR 2.9966 3.3288 20.1141 1.000
C1 C 0.2557 3.3125 20.9595 1.000
C7 C -2.1986 3.2581 21.7866 1.000
C6 C 1.8622 10.1483 7.0260 1.000
H7 H -0.2620 9.9516 8.1120 1.000
C6 C 0.8175 6.7474 19.9142 1.000
H7 H 2.9417 6.5507 18.8283 1.000
O1 O 12.3393 3.3057 8.7004 1.000
C2 C 12.9086 3.3601 6.3539 1.000
H3 H 13.0842 3.4162 4.2594 1.000
H9A H 10.0389 4.3940 11.7804 1.000
H9B H 10.3215 2.6666 11.7383 1.000
H1 H -6.0640 0.4474 4.2122 1.000
H1 H -6.0640 7.2492 4.2122 1.000
C8 C -8.5333 3.3329 10.5027 1.000
H16A H -1.6290 8.0441 -0.3704 1.000
H16B H -2.9005 8.7766 0.5873 1.000
C6 C -7.8515 -0.0544 19.9142 1.000
H7 H -5.7273 -0.2511 18.8283 1.000
C6 C -7.8515 6.7474 19.9142 1.000
H7 H -5.7273 6.5507 18.8283 1.000

C1 C 3.2503 -3.4893 7.4894 1.000
C5 C 1.5093 -3.5301 5.9228 1.000
H10 H 9.2849 3.2233 13.8698 1.000
C7 C 3.7907 3.2581 -5.1537 1.000
C1 C -0.5706 -0.0884 19.4509 1.000
C5 C 1.1704 -0.1292 21.0175 1.000
O1 O 0.6756 3.3057 22.1705 1.000
N1 N -1.8776 3.3057 22.7955 1.000
H7 H -3.2566 3.1498 21.5821 1.000
C1 C 3.2503 10.1143 7.4894 1.000
C5 C 1.5093 10.0735 5.9228 1.000
C1 C -0.5706 6.7134 19.4509 1.000
C5 C 1.1704 6.6726 21.0175 1.000
H1 H 11.7384 3.8483 9.2579 1.000
Br1 BR 14.6603 3.3288 6.6440 1.000
C14 C -9.4474 4.5096 10.5202 1.000
C13 C -9.4264 1.9929 10.4259 1.000
C9 C -7.7866 3.4281 11.7338 1.000
C1 C -9.2396 -0.0884 19.4509 1.000
C5 C -7.4986 -0.1292 21.0175 1.000
C1 C -9.2396 6.7134 19.4509 1.000
C5 C -7.4986 6.6726 21.0175 1.000
O1 O 3.6703 -3.4961 8.7004 1.000
C2 C 4.2396 -3.4417 6.3539 1.000
H5 H 0.4482 -3.6061 5.7204 1.000
C4 C 2.3267 -3.5233 4.9368 1.000
C6 C 4.8569 3.3465 -6.4441 1.000
H7 H 2.7326 3.1498 -5.3582 1.000
O1 O -0.9906 -0.0952 18.2399 1.000
C2 C -1.5599 -0.0408 20.5864 1.000
H5 H 2.2315 -0.2052 21.2199 1.000
C4 C 0.3530 -0.1224 22.0035 1.000
H1 H 0.0747 3.8483 22.7280 1.000
C8 C -2.8589 3.3329 23.9728 1.000
O1 O 3.6703 10.1075 8.7004 1.000
C2 C 4.2396 10.1619 6.3539 1.000
H5 H 0.4482 9.9975 5.7204 1.000
C4 C 2.3267 10.0803 4.9368 1.000
O1 O -0.9906 6.7066 18.2399 1.000
C2 C -1.5599 6.7610 20.5864 1.000
H5 H 2.2315 6.5966 21.2199 1.000
C4 C 0.3530 6.6794 22.0035 1.000
H14A H -10.0786 4.4731 9.6408 1.000
H14B H -8.8534 5.4126 10.4517 1.000
C15 C -10.3444 4.5912 11.7864 1.000
C12 C -10.3913 2.1018 11.6853 1.000

H13A H -9.9952 1.9550 9.5051 1.000
H13B H -8.8059 1.1076 10.4900 1.000
C10 C -8.6696 3.2513 12.9798 1.000
H9A H -7.2991 4.3940 11.7804 1.000
H9B H -7.0165 2.6666 11.7383 1.000
O1 O -9.6596 -0.0952 18.2399 1.000
C2 C -10.2289 -0.0408 20.5864 1.000
H5 H -6.4375 -0.2052 21.2199 1.000
C4 C -8.3160 -0.1224 22.0035 1.000
O1 O -9.6596 6.7066 18.2399 1.000
C2 C -10.2289 6.7610 20.5864 1.000
H5 H -6.4375 6.5966 21.2199 1.000
C4 C -8.3160 6.6794 22.0035 1.000
H1 H 3.0694 -2.9535 9.2579 1.000
Br1 BR 5.9913 -3.4730 6.6440 1.000
C3 C 3.7531 -3.4417 5.1146 1.000
Cl1 CL 1.6075 -3.4485 3.4106 1.000
C1 C 6.2449 3.3125 -5.9807 1.000
C5 C 4.5040 3.2717 -7.5473 1.000
H1 H -0.3896 0.4474 17.6824 1.000
Br1 BR -3.3116 -0.0721 20.2963 1.000
C3 C -1.0734 -0.0408 21.8257 1.000
Cl1 CL 1.0722 -0.0476 23.5296 1.000
C14 C -3.7730 4.5096 23.9903 1.000
C13 C -3.7521 1.9929 23.8960 1.000
C9 C -2.1123 3.4281 25.2040 1.000
H1 H 3.0694 10.6501 9.2579 1.000
Br1 BR 5.9913 10.1306 6.6440 1.000
C3 C 3.7531 10.1619 5.1146 1.000
Cl1 CL 1.6075 10.1551 3.4106 1.000
H1 H -0.3896 7.2492 17.6824 1.000
Br1 BR -3.3116 6.7297 20.2963 1.000
C3 C -1.0734 6.7610 21.8257 1.000
Cl1 CL 1.0722 6.7542 23.5296 1.000
H15 H -10.9212 5.5078 11.7825 1.000
C17 C -11.2609 3.3873 11.6584 1.000
C16 C -9.4546 4.5436 12.9313 1.000
H12 H -11.0534 1.2447 11.6794 1.000
C11 C -9.5820 2.0609 12.9583 1.000
H10 H -8.0531 3.2233 13.8698 1.000
H1 H -9.0586 0.4474 17.6824 1.000
Br1 BR -11.9806 -0.0721 20.2963 1.000
C3 C -9.7424 -0.0408 21.8257 1.000
Cl1 CL -7.5968 -0.0476 23.5296 1.000
H1 H -9.0586 7.2492 17.6824 1.000
Br1 BR -11.9806 6.7297 20.2963 1.000

C3 C -9.7424 6.7610 21.8257 1.000
Cl1 CL -7.5968 6.7542 23.5296 1.000
H3 H 4.4152 -3.3856 4.2594 1.000
O1 O 6.6649 3.3057 -4.7698 1.000
C2 C 7.2343 3.3601 -7.1163 1.000
H5 H 3.4428 3.1957 -7.7498 1.000
C4 C 5.3214 3.2785 -8.5333 1.000
H3 H -1.7355 0.0153 22.6809 1.000
H14A H -4.4042 4.4731 23.1110 1.000
H14B H -3.1791 5.4126 23.9219 1.000
C15 C -4.6700 4.5912 25.2565 1.000
C12 C -4.7169 2.1018 25.1555 1.000
H13A H -4.3208 1.9550 22.9752 1.000
H13B H -3.1316 1.1076 23.9601 1.000
C10 C -2.9952 3.2513 26.4500 1.000
H9A H -1.6248 4.3940 25.2506 1.000
H9B H -1.3422 2.6666 25.2084 1.000
H3 H 4.4152 10.2180 4.2594 1.000
H3 H -1.7355 6.8171 22.6809 1.000
H17A H -11.8163 3.4339 10.7299 1.000
H17B H -11.9666 3.3694 12.4797 1.000
H16A H -10.0346 4.6432 13.8406 1.000
H16B H -8.7632 5.3757 12.8828 1.000
H11A H -10.2461 2.0811 13.8135 1.000
H11B H -9.0029 1.1464 12.9901 1.000
H3 H -10.4045 0.0153 22.6809 1.000
H3 H -10.4045 6.8171 22.6809 1.000
H1 H 6.0640 3.8483 -4.2122 1.000
Br1 BR 8.9859 3.3288 -6.8261 1.000
C3 C 6.7478 3.3601 -8.3555 1.000
Cl1 CL 4.6022 3.3533 -10.0595 1.000
H15 H -5.2469 5.5078 25.2526 1.000
C17 C -5.5865 3.3873 25.1285 1.000
C16 C -3.7803 4.5436 26.4015 1.000
H12 H -5.3790 1.2447 25.1495 1.000
C11 C -3.9076 2.0609 26.4284 1.000
H10 H -2.3787 3.2233 27.3399 1.000
H3 H 7.4098 3.4162 -9.2107 1.000
H17A H -6.1419 3.4339 24.2000 1.000
H17B H -6.2923 3.3694 25.9498 1.000
H16A H -4.3603 4.6432 27.3107 1.000
H16B H -3.0888 5.3757 26.3530 1.000
H11A H -4.5718 2.0811 27.2836 1.000
H11B H -3.3285 1.1464 26.4602 1.000

All Atoms [15 molecules]
600 atoms, Cartesian coordinates
Label Symbol x y z Occ.

[-x, y+1/2, -z]
H17A H 5.5217 3.4339 10.7299 1.000
C17 C 6.0771 3.3873 11.6584 1.000
C15 C 6.9936 4.5912 11.7864 1.000
H17B H 5.3714 3.3694 12.4797 1.000
C12 C 6.9467 2.1018 11.6853 1.000
C14 C 7.8906 4.5096 10.5202 1.000
H15 H 6.4168 5.5078 11.7825 1.000
C16 C 7.8834 4.5436 12.9313 1.000
C13 C 7.9116 1.9929 10.4259 1.000
H12 H 6.2846 1.2447 11.6794 1.000
C11 C 7.7560 2.0609 12.9583 1.000
H14A H 7.2594 4.4731 9.6408 1.000
C8 C 8.8047 3.3329 10.5027 1.000
H14B H 8.4846 5.4126 10.4517 1.000
H16A H 7.3034 4.6432 13.8406 1.000
H16B H 8.5748 5.3757 12.8828 1.000
C10 C 8.6684 3.2513 12.9798 1.000
H13A H 7.3428 1.9550 9.5051 1.000
H13B H 8.5321 1.1076 10.4900 1.000
H11A H 7.0919 2.0811 13.8135 1.000
H11B H 8.3351 1.1464 12.9901 1.000
N1 N 9.7860 3.3057 9.3254 1.000
C9 C 9.5514 3.4281 11.7338 1.000
H10 H 9.2849 3.2233 13.8698 1.000
C7 C 9.4650 3.2581 8.3165 1.000
H9A H 10.0389 4.3940 11.7804 1.000
H9B H 10.3215 2.6666 11.7383 1.000
H7 H 8.4070 3.1498 8.1120 1.000
C6 C 10.5312 3.3465 7.0260 1.000
C5 C 10.1783 3.2717 5.9228 1.000
C1 C 11.9193 3.3125 7.4894 1.000
H5 H 9.1172 3.1957 5.7204 1.000
C4 C 10.9957 3.2785 4.9368 1.000
O1 O 12.3393 3.3057 8.7004 1.000
C2 C 12.9086 3.3601 6.3539 1.000
C3 C 12.4221 3.3601 5.1146 1.000
Cl1 CL 10.2765 3.3533 3.4106 1.000
H1 H 11.7384 3.8483 9.2579 1.000
Br1 BR 14.6603 3.3288 6.6440 1.000
H3 H 13.0842 3.4162 4.2594 1.000

[-x, y+1/2, -z]
H16A H 1.6290 4.6432 0.3704 1.000
C16 C 2.2090 4.5436 -0.5388 1.000
C10 C 2.9940 3.2513 -0.4903 1.000
C15 C 1.3193 4.5912 -1.6838 1.000
H16B H 2.9005 5.3757 -0.5873 1.000
H10 H 3.6106 3.2233 0.3997 1.000
C11 C 2.0817 2.0609 -0.5119 1.000
C9 C 3.8770 3.4281 -1.7363 1.000
C14 C 2.2162 4.5096 -2.9500 1.000
H15 H 0.7424 5.5078 -1.6877 1.000
C17 C 0.4028 3.3873 -1.8117 1.000
H11A H 1.4175 2.0811 0.3434 1.000
C12 C 1.2724 2.1018 -1.7848 1.000
H11B H 2.6608 1.1464 -0.4801 1.000
C8 C 3.1304 3.3329 -2.9675 1.000
H9A H 4.3645 4.3940 -1.6897 1.000
H9B H 4.6471 2.6666 -1.7319 1.000
H14A H 1.5851 4.4731 -3.8293 1.000
H14B H 2.8102 5.4126 -3.0184 1.000
H17A H -0.1526 3.4339 -2.7402 1.000
H17B H -0.3030 3.3694 -0.9905 1.000
H12 H 0.6102 1.2447 -1.7907 1.000
C13 C 2.2372 1.9929 -3.0443 1.000
N1 N 4.1116 3.3057 -4.1448 1.000
H13A H 1.6685 1.9550 -3.9651 1.000
H13B H 2.8577 1.1076 -2.9801 1.000
C7 C 3.7907 3.2581 -5.1537 1.000
C6 C 4.8569 3.3465 -6.4441 1.000
H7 H 2.7326 3.1498 -5.3582 1.000
C1 C 6.2449 3.3125 -5.9807 1.000
C5 C 4.5040 3.2717 -7.5473 1.000
O1 O 6.6649 3.3057 -4.7698 1.000
C2 C 7.2343 3.3601 -7.1163 1.000
H5 H 3.4428 3.1957 -7.7498 1.000
C4 C 5.3214 3.2785 -8.5333 1.000
H1 H 6.0640 3.8483 -4.2122 1.000
Br1 BR 8.9859 3.3288 -6.8261 1.000
C3 C 6.7478 3.3601 -8.3555 1.000
Cl1 CL 4.6022 3.3533 -10.0595 1.000
H3 H 7.4098 3.4162 -9.2107 1.000

[x, y, z]
H10 H 2.0638 -0.1776 13.0705 1.000
C10 C 2.6803 -0.1496 13.9604 1.000
C16 C 3.4653 1.1427 14.0089 1.000

C9 C 1.7973 0.0272 15.2064 1.000
C11 C 3.5927 -1.3400 13.9820 1.000
H16A H 4.0454 1.2423 13.0997 1.000
H16B H 2.7739 1.9748 14.0574 1.000
C15 C 4.3551 1.1903 15.1539 1.000
H9B H 1.0272 -0.7343 15.2020 1.000
H9A H 1.3098 0.9931 15.1598 1.000
C8 C 2.5440 -0.0680 16.4376 1.000
C12 C 4.4020 -1.2991 15.2549 1.000
H11A H 4.2568 -1.3198 13.1268 1.000
H11B H 3.0136 -2.2545 13.9502 1.000
C14 C 3.4581 1.1087 16.4201 1.000
H15 H 4.9319 2.1069 15.1578 1.000
C17 C 5.2716 -0.0136 15.2819 1.000
N1 N 1.5627 -0.0952 17.6149 1.000
C13 C 3.4371 -1.4080 16.5144 1.000
H12 H 5.0641 -2.1562 15.2609 1.000
H14B H 2.8642 2.0117 16.4885 1.000
H14A H 4.0893 1.0722 17.2994 1.000
H17A H 5.8270 0.0330 16.2104 1.000
H17B H 5.9773 -0.0315 14.4606 1.000
C7 C 1.8837 -0.1428 18.6238 1.000
H13A H 4.0059 -1.4459 17.4352 1.000
H13B H 2.8167 -2.2933 16.4503 1.000
C6 C 0.8175 -0.0544 19.9142 1.000
H7 H 2.9417 -0.2511 18.8283 1.000
C1 C -0.5706 -0.0884 19.4509 1.000
C5 C 1.1704 -0.1292 21.0175 1.000
O1 O -0.9906 -0.0952 18.2399 1.000
C2 C -1.5599 -0.0408 20.5864 1.000
H5 H 2.2315 -0.2052 21.2199 1.000
C4 C 0.3530 -0.1224 22.0035 1.000
H1 H -0.3896 0.4474 17.6824 1.000
Br1 BR -3.3116 -0.0721 20.2963 1.000
C3 C -1.0734 -0.0408 21.8257 1.000
Cl1 CL 1.0722 -0.0476 23.5296 1.000
H3 H -1.7355 0.0153 22.6809 1.000

[x, y, z]
Br1 BR -0.3169 6.7297 6.8261 1.000
C2 C 1.4347 6.7610 7.1163 1.000
C3 C 1.9212 6.7610 8.3555 1.000
C1 C 2.4241 6.7134 5.9807 1.000
C4 C 3.3476 6.6794 8.5333 1.000
H3 H 1.2592 6.8171 9.2107 1.000
C6 C 3.8121 6.7474 6.4441 1.000

O1 O 2.0041 6.7066 4.7698 1.000
C5 C 4.1650 6.6726 7.5473 1.000
Cl1 CL 4.0668 6.7542 10.0595 1.000
C7 C 4.8783 6.6590 5.1537 1.000
H1 H 2.6050 7.2492 4.2122 1.000
H5 H 5.2262 6.5966 7.7498 1.000
N1 N 4.5574 6.7066 4.1448 1.000
H7 H 5.9364 6.5507 5.3582 1.000
C8 C 5.5386 6.7338 2.9675 1.000
C13 C 6.4318 5.3938 3.0443 1.000
C14 C 6.4528 7.9105 2.9500 1.000
C9 C 4.7920 6.8290 1.7363 1.000
H13A H 7.0005 5.3559 3.9651 1.000
H13B H 5.8113 4.5085 2.9801 1.000
C12 C 7.3966 5.5027 1.7848 1.000
H14A H 7.0839 7.8740 3.8293 1.000
H14B H 5.8588 8.8135 3.0184 1.000
C15 C 7.3497 7.9921 1.6838 1.000
H9B H 4.0219 6.0675 1.7319 1.000
C10 C 5.6750 6.6522 0.4903 1.000
H9A H 4.3045 7.7949 1.6897 1.000
C17 C 8.2662 6.7882 1.8117 1.000
H12 H 8.0588 4.6456 1.7907 1.000
C11 C 6.5873 5.4618 0.5119 1.000
H15 H 7.9266 8.9087 1.6877 1.000
C16 C 6.4600 7.9445 0.5388 1.000
H10 H 5.0584 6.6242 -0.3997 1.000
H17A H 8.8216 6.8348 2.7402 1.000
H17B H 8.9720 6.7703 0.9905 1.000
H11A H 7.2515 5.4820 -0.3434 1.000
H11B H 6.0082 4.5473 0.4801 1.000
H16A H 7.0400 8.0441 -0.3704 1.000
H16B H 5.7685 8.7766 0.5873 1.000

[-x, y+1/2, -z]
C12 C -1.7223 8.9036 11.6853 1.000
H12 H -2.3844 8.0465 11.6794 1.000
C11 C -0.9130 8.8627 12.9583 1.000
C13 C -0.7574 8.7947 10.4259 1.000
C17 C -2.5919 10.1891 11.6584 1.000
H11B H -0.3339 7.9482 12.9901 1.000
H11A H -1.5771 8.8829 13.8135 1.000
C10 C -0.0006 10.0531 12.9798 1.000
H13A H -1.3262 8.7568 9.5051 1.000
H13B H -0.1369 7.9094 10.4900 1.000
C8 C 0.1357 10.1347 10.5027 1.000

C15 C -1.6754 11.3930 11.7864 1.000
H17A H -3.1473 10.2357 10.7299 1.000
H17B H -3.2976 10.1712 12.4797 1.000
C16 C -0.7856 11.3454 12.9313 1.000
H10 H 0.6159 10.0251 13.8698 1.000
C9 C 0.8824 10.2299 11.7338 1.000
N1 N 1.1170 10.1075 9.3254 1.000
C14 C -0.7784 11.3114 10.5202 1.000
H15 H -2.2522 12.3096 11.7825 1.000
H16A H -1.3656 11.4450 13.8406 1.000
H16B H -0.0942 12.1775 12.8828 1.000
H9A H 1.3699 11.1958 11.7804 1.000
H9B H 1.6525 9.4684 11.7383 1.000
C7 C 0.7960 10.0599 8.3165 1.000
H14A H -1.4096 11.2749 9.6408 1.000
H14B H -0.1844 12.2144 10.4517 1.000
C6 C 1.8622 10.1483 7.0260 1.000
H7 H -0.2620 9.9516 8.1120 1.000
C1 C 3.2503 10.1143 7.4894 1.000
C5 C 1.5093 10.0735 5.9228 1.000
O1 O 3.6703 10.1075 8.7004 1.000
C2 C 4.2396 10.1619 6.3539 1.000
H5 H 0.4482 9.9975 5.7204 1.000
C4 C 2.3267 10.0803 4.9368 1.000
H1 H 3.0694 10.6501 9.2579 1.000
Br1 BR 5.9913 10.1306 6.6440 1.000
C3 C 3.7531 10.1619 5.1146 1.000
Cl1 CL 1.6075 10.1551 3.4106 1.000
H3 H 4.4152 10.2180 4.2594 1.000

[x, y, z]
H11A H 4.2568 5.4820 13.1268 1.000
C11 C 3.5927 5.4618 13.9820 1.000
H11B H 3.0136 4.5473 13.9502 1.000
C10 C 2.6803 6.6522 13.9604 1.000
C12 C 4.4020 5.5027 15.2549 1.000
H10 H 2.0638 6.6242 13.0705 1.000
C9 C 1.7973 6.8290 15.2064 1.000
C16 C 3.4653 7.9445 14.0089 1.000
C13 C 3.4371 5.3938 16.5144 1.000
C17 C 5.2716 6.7882 15.2819 1.000
H12 H 5.0641 4.6456 15.2609 1.000
H9B H 1.0272 6.0675 15.2020 1.000
H9A H 1.3098 7.7949 15.1598 1.000
C8 C 2.5440 6.7338 16.4376 1.000
C15 C 4.3551 7.9921 15.1539 1.000

H16A H 4.0454 8.0441 13.0997 1.000
H16B H 2.7739 8.7766 14.0574 1.000
H13B H 2.8167 4.5085 16.4503 1.000
H13A H 4.0059 5.3559 17.4352 1.000
H17A H 5.8270 6.8348 16.2104 1.000
H17B H 5.9773 6.7703 14.4606 1.000
N1 N 1.5627 6.7066 17.6149 1.000
C14 C 3.4581 7.9105 16.4201 1.000
H15 H 4.9319 8.9087 15.1578 1.000
C7 C 1.8837 6.6590 18.6238 1.000
H14A H 4.0893 7.8740 17.2994 1.000
H14B H 2.8642 8.8135 16.4885 1.000
C6 C 0.8175 6.7474 19.9142 1.000
H7 H 2.9417 6.5507 18.8283 1.000
C1 C -0.5706 6.7134 19.4509 1.000
C5 C 1.1704 6.6726 21.0175 1.000
O1 O -0.9906 6.7066 18.2399 1.000
C2 C -1.5599 6.7610 20.5864 1.000
H5 H 2.2315 6.5966 21.2199 1.000
C4 C 0.3530 6.6794 22.0035 1.000
H1 H -0.3896 7.2492 17.6824 1.000
Br1 BR -3.3116 6.7297 20.2963 1.000
C3 C -1.0734 6.7610 21.8257 1.000
Cl1 CL 1.0722 6.7542 23.5296 1.000
H3 H -1.7355 6.8171 22.6809 1.000

[-x, y+1/2, -z]
Cl1 CL -1.3871 3.3533 16.8808 1.000
C4 C -0.6679 3.2785 18.4069 1.000
C3 C 0.7585 3.3601 18.5847 1.000
C5 C -1.4853 3.2717 19.3930 1.000
H3 H 1.4205 3.4162 17.7295 1.000
C2 C 1.2450 3.3601 19.8240 1.000
H5 H -2.5464 3.1957 19.1905 1.000
C6 C -1.1324 3.3465 20.4962 1.000
Br1 BR 2.9966 3.3288 20.1141 1.000
C1 C 0.2557 3.3125 20.9595 1.000
C7 C -2.1986 3.2581 21.7866 1.000
O1 O 0.6756 3.3057 22.1705 1.000
N1 N -1.8776 3.3057 22.7955 1.000
H7 H -3.2566 3.1498 21.5821 1.000
H1 H 0.0747 3.8483 22.7280 1.000
C8 C -2.8589 3.3329 23.9728 1.000
C14 C -3.7730 4.5096 23.9903 1.000
C13 C -3.7521 1.9929 23.8960 1.000
C9 C -2.1123 3.4281 25.2040 1.000

H14A H -4.4042 4.4731 23.1110 1.000
H14B H -3.1791 5.4126 23.9219 1.000
C15 C -4.6700 4.5912 25.2565 1.000
C12 C -4.7169 2.1018 25.1555 1.000
H13A H -4.3208 1.9550 22.9752 1.000
H13B H -3.1316 1.1076 23.9601 1.000
C10 C -2.9952 3.2513 26.4500 1.000
H9A H -1.6248 4.3940 25.2506 1.000
H9B H -1.3422 2.6666 25.2084 1.000
H15 H -5.2469 5.5078 25.2526 1.000
C17 C -5.5865 3.3873 25.1285 1.000
C16 C -3.7803 4.5436 26.4015 1.000
H12 H -5.3790 1.2447 25.1495 1.000
C11 C -3.9076 2.0609 26.4284 1.000
H10 H -2.3787 3.2233 27.3399 1.000
H17A H -6.1419 3.4339 24.2000 1.000
H17B H -6.2923 3.3694 25.9498 1.000
H16A H -4.3603 4.6432 27.3107 1.000
H16B H -3.0888 5.3757 26.3530 1.000
H11A H -4.5718 2.0811 27.2836 1.000
H11B H -3.3285 1.1464 26.4602 1.000

[-x, y+1/2, -z]
Br1 BR -2.6777 3.3288 6.6440 1.000
C2 C -4.4294 3.3601 6.3539 1.000
C1 C -5.4187 3.3125 7.4894 1.000
C3 C -4.9159 3.3601 5.1146 1.000
O1 O -4.9987 3.3057 8.7004 1.000
C6 C -6.8068 3.3465 7.0260 1.000
H3 H -4.2538 3.4162 4.2594 1.000
C4 C -6.3423 3.2785 4.9368 1.000
H1 H -5.5996 3.8483 9.2579 1.000
C5 C -7.1597 3.2717 5.9228 1.000
C7 C -7.8730 3.2581 8.3165 1.000
Cl1 CL -7.0615 3.3533 3.4106 1.000
H5 H -8.2208 3.1957 5.7204 1.000
N1 N -7.5520 3.3057 9.3254 1.000
H7 H -8.9310 3.1498 8.1120 1.000
C8 C -8.5333 3.3329 10.5027 1.000
C14 C -9.4474 4.5096 10.5202 1.000
C13 C -9.4264 1.9929 10.4259 1.000
C9 C -7.7866 3.4281 11.7338 1.000
H14A H -10.0786 4.4731 9.6408 1.000
H14B H -8.8534 5.4126 10.4517 1.000
C15 C -10.3444 4.5912 11.7864 1.000
C12 C -10.3913 2.1018 11.6853 1.000

H13A H -9.9952 1.9550 9.5051 1.000
H13B H -8.8059 1.1076 10.4900 1.000
C10 C -8.6696 3.2513 12.9798 1.000
H9A H -7.2991 4.3940 11.7804 1.000
H9B H -7.0165 2.6666 11.7383 1.000
H15 H -10.9212 5.5078 11.7825 1.000
C17 C -11.2609 3.3873 11.6584 1.000
C16 C -9.4546 4.5436 12.9313 1.000
H12 H -11.0534 1.2447 11.6794 1.000
C11 C -9.5820 2.0609 12.9583 1.000
H10 H -8.0531 3.2233 13.8698 1.000
H17A H -11.8163 3.4339 10.7299 1.000
H17B H -11.9666 3.3694 12.4797 1.000
H16A H -10.0346 4.6432 13.8406 1.000
H16B H -8.7632 5.3757 12.8828 1.000
H11A H -10.2461 2.0811 13.8135 1.000
H11B H -9.0029 1.1464 12.9901 1.000

[x, y, z]
H5 H -3.4428 6.5966 7.7498 1.000
C5 C -4.5040 6.6726 7.5473 1.000
C4 C -5.3214 6.6794 8.5333 1.000
C6 C -4.8569 6.7474 6.4441 1.000
Cl1 CL -4.6022 6.7542 10.0595 1.000
C3 C -6.7478 6.7610 8.3555 1.000
C1 C -6.2449 6.7134 5.9807 1.000
C7 C -3.7907 6.6590 5.1537 1.000
H3 H -7.4098 6.8171 9.2107 1.000
C2 C -7.2343 6.7610 7.1163 1.000
O1 O -6.6649 6.7066 4.7698 1.000
N1 N -4.1116 6.7066 4.1448 1.000
H7 H -2.7326 6.5507 5.3582 1.000
Br1 BR -8.9859 6.7297 6.8261 1.000
H1 H -6.0640 7.2492 4.2122 1.000
C8 C -3.1304 6.7338 2.9675 1.000
C13 C -2.2372 5.3938 3.0443 1.000
C14 C -2.2162 7.9105 2.9500 1.000
C9 C -3.8770 6.8290 1.7363 1.000
H13A H -1.6685 5.3559 3.9651 1.000
C12 C -1.2724 5.5027 1.7848 1.000
H13B H -2.8577 4.5085 2.9801 1.000
C15 C -1.3193 7.9921 1.6838 1.000
H14A H -1.5851 7.8740 3.8293 1.000
H14B H -2.8102 8.8135 3.0184 1.000
C10 C -2.9940 6.6522 0.4903 1.000
H9A H -4.3645 7.7949 1.6897 1.000

H9B H -4.6471 6.0675 1.7319 1.000
H12 H -0.6102 4.6456 1.7907 1.000
C17 C -0.4028 6.7882 1.8117 1.000
C11 C -2.0817 5.4618 0.5119 1.000
H15 H -0.7424 8.9087 1.6877 1.000
C16 C -2.2090 7.9445 0.5388 1.000
H10 H -3.6106 6.6242 -0.3997 1.000
H17A H 0.1526 6.8348 2.7402 1.000
H17B H 0.3030 6.7703 0.9905 1.000
H11A H -1.4175 5.4820 -0.3434 1.000
H11B H -2.6608 4.5473 0.4801 1.000
H16A H -1.6290 8.0441 -0.3704 1.000
H16B H -2.9005 8.7766 0.5873 1.000

[x, y, z]
H5 H -3.4428 -0.2052 7.7498 1.000
C5 C -4.5040 -0.1292 7.5473 1.000
C4 C -5.3214 -0.1224 8.5333 1.000
C6 C -4.8569 -0.0544 6.4441 1.000
Cl1 CL -4.6022 -0.0476 10.0595 1.000
C3 C -6.7478 -0.0408 8.3555 1.000
C1 C -6.2449 -0.0884 5.9807 1.000
C7 C -3.7907 -0.1428 5.1537 1.000
H3 H -7.4098 0.0153 9.2107 1.000
C2 C -7.2343 -0.0408 7.1163 1.000
O1 O -6.6649 -0.0952 4.7698 1.000
N1 N -4.1116 -0.0952 4.1448 1.000
H7 H -2.7326 -0.2511 5.3582 1.000
Br1 BR -8.9859 -0.0721 6.8261 1.000
H1 H -6.0640 0.4474 4.2122 1.000
C8 C -3.1304 -0.0680 2.9675 1.000
C14 C -2.2162 1.1087 2.9500 1.000
C13 C -2.2372 -1.4080 3.0443 1.000
C9 C -3.8770 0.0272 1.7363 1.000
H14A H -1.5851 1.0722 3.8293 1.000
C15 C -1.3193 1.1903 1.6838 1.000
H14B H -2.8102 2.0117 3.0184 1.000
C12 C -1.2724 -1.2991 1.7848 1.000
H13A H -1.6685 -1.4459 3.9651 1.000
H13B H -2.8577 -2.2933 2.9801 1.000
C10 C -2.9940 -0.1496 0.4903 1.000
H9A H -4.3645 0.9931 1.6897 1.000
H9B H -4.6471 -0.7343 1.7319 1.000
H15 H -0.7424 2.1069 1.6877 1.000
C17 C -0.4028 -0.0136 1.8117 1.000
C16 C -2.2090 1.1427 0.5388 1.000

H12 H -0.6102 -2.1562 1.7907 1.000
C11 C -2.0817 -1.3400 0.5119 1.000
H10 H -3.6106 -0.1776 -0.3997 1.000
H17A H 0.1526 0.0330 2.7402 1.000
H17B H 0.3030 -0.0315 0.9905 1.000
H16A H -1.6290 1.2423 -0.3704 1.000
H16B H -2.9005 1.9748 0.5873 1.000
H11A H -1.4175 -1.3198 -0.3434 1.000
H11B H -2.6608 -2.2545 0.4801 1.000

[x, y, z]
H11A H -4.4122 -1.3198 13.1268 1.000
C11 C -5.0763 -1.3400 13.9820 1.000
C10 C -5.9887 -0.1496 13.9604 1.000
C12 C -4.2670 -1.2991 15.2549 1.000
H11B H -5.6554 -2.2545 13.9502 1.000
C16 C -5.2037 1.1427 14.0089 1.000
H10 H -6.6052 -0.1776 13.0705 1.000
C9 C -6.8717 0.0272 15.2064 1.000
C17 C -3.3974 -0.0136 15.2819 1.000
H12 H -3.6049 -2.1562 15.2609 1.000
C13 C -5.2319 -1.4080 16.5144 1.000
H16A H -4.6236 1.2423 13.0997 1.000
C15 C -4.3139 1.1903 15.1539 1.000
H16B H -5.8951 1.9748 14.0574 1.000
C8 C -6.1250 -0.0680 16.4376 1.000
H9A H -7.3592 0.9931 15.1598 1.000
H9B H -7.6418 -0.7343 15.2020 1.000
H17B H -2.6917 -0.0315 14.4606 1.000
H17A H -2.8420 0.0330 16.2104 1.000
H13A H -4.6631 -1.4459 17.4352 1.000
H13B H -5.8523 -2.2933 16.4503 1.000
H15 H -3.7371 2.1069 15.1578 1.000
C14 C -5.2109 1.1087 16.4201 1.000
N1 N -7.1063 -0.0952 17.6149 1.000
H14A H -4.5797 1.0722 17.2994 1.000
H14B H -5.8048 2.0117 16.4885 1.000
C7 C -6.7853 -0.1428 18.6238 1.000
C6 C -7.8515 -0.0544 19.9142 1.000
H7 H -5.7273 -0.2511 18.8283 1.000
C1 C -9.2396 -0.0884 19.4509 1.000
C5 C -7.4986 -0.1292 21.0175 1.000
O1 O -9.6596 -0.0952 18.2399 1.000
C2 C -10.2289 -0.0408 20.5864 1.000
H5 H -6.4375 -0.2052 21.2199 1.000
C4 C -8.3160 -0.1224 22.0035 1.000

H1 H -9.0586 0.4474 17.6824 1.000
Br1 BR -11.9806 -0.0721 20.2963 1.000
C3 C -9.7424 -0.0408 21.8257 1.000
Cl1 CL -7.5968 -0.0476 23.5296 1.000
H3 H -10.4045 0.0153 22.6809 1.000

[x, y, z]
Br1 BR -0.3169 -0.0721 6.8261 1.000
C2 C 1.4347 -0.0408 7.1163 1.000
C3 C 1.9212 -0.0408 8.3555 1.000
C1 C 2.4241 -0.0884 5.9807 1.000
C4 C 3.3476 -0.1224 8.5333 1.000
H3 H 1.2592 0.0153 9.2107 1.000
C6 C 3.8121 -0.0544 6.4441 1.000
O1 O 2.0041 -0.0952 4.7698 1.000
C5 C 4.1650 -0.1292 7.5473 1.000
Cl1 CL 4.0668 -0.0476 10.0595 1.000
C7 C 4.8783 -0.1428 5.1537 1.000
H1 H 2.6050 0.4474 4.2122 1.000
H5 H 5.2262 -0.2052 7.7498 1.000
N1 N 4.5574 -0.0952 4.1448 1.000
H7 H 5.9364 -0.2511 5.3582 1.000
C8 C 5.5386 -0.0680 2.9675 1.000
C14 C 6.4528 1.1087 2.9500 1.000
C9 C 4.7920 0.0272 1.7363 1.000
C13 C 6.4318 -1.4080 3.0443 1.000
H14A H 7.0839 1.0722 3.8293 1.000
H14B H 5.8588 2.0117 3.0184 1.000
C15 C 7.3497 1.1903 1.6838 1.000
H9A H 4.3045 0.9931 1.6897 1.000
C10 C 5.6750 -0.1496 0.4903 1.000
H9B H 4.0219 -0.7343 1.7319 1.000
C12 C 7.3966 -1.2991 1.7848 1.000
H13A H 7.0005 -1.4459 3.9651 1.000
H13B H 5.8113 -2.2933 2.9801 1.000
H15 H 7.9266 2.1069 1.6877 1.000
C17 C 8.2662 -0.0136 1.8117 1.000
C16 C 6.4600 1.1427 0.5388 1.000
C11 C 6.5873 -1.3400 0.5119 1.000
H10 H 5.0584 -0.1776 -0.3997 1.000
H12 H 8.0588 -2.1562 1.7907 1.000
H17A H 8.8216 0.0330 2.7402 1.000
H17B H 8.9720 -0.0315 0.9905 1.000
H16A H 7.0400 1.2423 -0.3704 1.000
H16B H 5.7685 1.9748 0.5873 1.000
H11A H 7.2515 -1.3198 -0.3434 1.000

H11B H 6.0082 -2.2545 0.4801 1.000

[-x, y+1/2, -z]
C14 C -0.7784 -2.2922 10.5202 1.000
H14A H -1.4096 -2.3287 9.6408 1.000
H14B H -0.1844 -1.3892 10.4517 1.000
C15 C -1.6754 -2.2106 11.7864 1.000
C8 C 0.1357 -3.4689 10.5027 1.000
H15 H -2.2522 -1.2940 11.7825 1.000
C16 C -0.7856 -2.2582 12.9313 1.000
C17 C -2.5919 -3.4145 11.6584 1.000
N1 N 1.1170 -3.4961 9.3254 1.000
C13 C -0.7574 -4.8089 10.4259 1.000
C9 C 0.8824 -3.3737 11.7338 1.000
H16B H -0.0942 -1.4261 12.8828 1.000
H16A H -1.3656 -2.1586 13.8406 1.000
C10 C -0.0006 -3.5505 12.9798 1.000
H17A H -3.1473 -3.3679 10.7299 1.000
H17B H -3.2976 -3.4324 12.4797 1.000
C12 C -1.7223 -4.7000 11.6853 1.000
C7 C 0.7960 -3.5437 8.3165 1.000
H13A H -1.3262 -4.8468 9.5051 1.000
H13B H -0.1369 -5.6942 10.4900 1.000
H9A H 1.3699 -2.4078 11.7804 1.000
H9B H 1.6525 -4.1352 11.7383 1.000
C11 C -0.9130 -4.7409 12.9583 1.000
H10 H 0.6159 -3.5785 13.8698 1.000
H12 H -2.3844 -5.5571 11.6794 1.000
C6 C 1.8622 -3.4553 7.0260 1.000
H7 H -0.2620 -3.6520 8.1120 1.000
H11A H -1.5771 -4.7207 13.8135 1.000
H11B H -0.3339 -5.6554 12.9901 1.000
C1 C 3.2503 -3.4893 7.4894 1.000
C5 C 1.5093 -3.5301 5.9228 1.000
O1 O 3.6703 -3.4961 8.7004 1.000
C2 C 4.2396 -3.4417 6.3539 1.000
H5 H 0.4482 -3.6061 5.7204 1.000
C4 C 2.3267 -3.5233 4.9368 1.000
H1 H 3.0694 -2.9535 9.2579 1.000
Br1 BR 5.9913 -3.4730 6.6440 1.000
C3 C 3.7531 -3.4417 5.1146 1.000
Cl1 CL 1.6075 -3.4485 3.4106 1.000
H3 H 4.4152 -3.3856 4.2594 1.000

[-x, y+1/2, -z]
H17A H -3.1473 3.4339 10.7299 1.000

C17 C -2.5919 3.3873 11.6584 1.000
H17B H -3.2976 3.3694 12.4797 1.000
C15 C -1.6754 4.5912 11.7864 1.000
C12 C -1.7223 2.1018 11.6853 1.000
C14 C -0.7784 4.5096 10.5202 1.000
H15 H -2.2522 5.5078 11.7825 1.000
C16 C -0.7856 4.5436 12.9313 1.000
H12 H -2.3844 1.2447 11.6794 1.000
C11 C -0.9130 2.0609 12.9583 1.000
C13 C -0.7574 1.9929 10.4259 1.000
C8 C 0.1357 3.3329 10.5027 1.000
H14A H -1.4096 4.4731 9.6408 1.000
H14B H -0.1844 5.4126 10.4517 1.000
H16B H -0.0942 5.3757 12.8828 1.000
C10 C -0.0006 3.2513 12.9798 1.000
H16A H -1.3656 4.6432 13.8406 1.000
H11B H -0.3339 1.1464 12.9901 1.000
H11A H -1.5771 2.0811 13.8135 1.000
H13A H -1.3262 1.9550 9.5051 1.000
H13B H -0.1369 1.1076 10.4900 1.000
N1 N 1.1170 3.3057 9.3254 1.000
C9 C 0.8824 3.4281 11.7338 1.000
H10 H 0.6159 3.2233 13.8698 1.000
C7 C 0.7960 3.2581 8.3165 1.000
H9A H 1.3699 4.3940 11.7804 1.000
H9B H 1.6525 2.6666 11.7383 1.000
C6 C 1.8622 3.3465 7.0260 1.000
H7 H -0.2620 3.1498 8.1120 1.000
C1 C 3.2503 3.3125 7.4894 1.000
C5 C 1.5093 3.2717 5.9228 1.000
O1 O 3.6703 3.3057 8.7004 1.000
C2 C 4.2396 3.3601 6.3539 1.000
H5 H 0.4482 3.1957 5.7204 1.000
C4 C 2.3267 3.2785 4.9368 1.000
H1 H 3.0694 3.8483 9.2579 1.000
Br1 BR 5.9913 3.3288 6.6440 1.000
C3 C 3.7531 3.3601 5.1146 1.000
Cl1 CL 1.6075 3.3533 3.4106 1.000
H3 H 4.4152 3.4162 4.2594 1.000

[x, y, z]
H11A H -4.4122 5.4820 13.1268 1.000
C11 C -5.0763 5.4618 13.9820 1.000
C12 C -4.2670 5.5027 15.2549 1.000
H11B H -5.6554 4.5473 13.9502 1.000
C10 C -5.9887 6.6522 13.9604 1.000

H12 H -3.6049 4.6456 15.2609 1.000
C17 C -3.3974 6.7882 15.2819 1.000
C13 C -5.2319 5.3938 16.5144 1.000
C16 C -5.2037 7.9445 14.0089 1.000
H10 H -6.6052 6.6242 13.0705 1.000
C9 C -6.8717 6.8290 15.2064 1.000
H17B H -2.6917 6.7703 14.4606 1.000
H17A H -2.8420 6.8348 16.2104 1.000
C15 C -4.3139 7.9921 15.1539 1.000
C8 C -6.1250 6.7338 16.4376 1.000
H13A H -4.6631 5.3559 17.4352 1.000
H13B H -5.8523 4.5085 16.4503 1.000
H16A H -4.6236 8.0441 13.0997 1.000
H16B H -5.8951 8.7766 14.0574 1.000
H9A H -7.3592 7.7949 15.1598 1.000
H9B H -7.6418 6.0675 15.2020 1.000
C14 C -5.2109 7.9105 16.4201 1.000
H15 H -3.7371 8.9087 15.1578 1.000
N1 N -7.1063 6.7066 17.6149 1.000
H14A H -4.5797 7.8740 17.2994 1.000
H14B H -5.8048 8.8135 16.4885 1.000
C7 C -6.7853 6.6590 18.6238 1.000
C6 C -7.8515 6.7474 19.9142 1.000
H7 H -5.7273 6.5507 18.8283 1.000
C1 C -9.2396 6.7134 19.4509 1.000
C5 C -7.4986 6.6726 21.0175 1.000
O1 O -9.6596 6.7066 18.2399 1.000
C2 C -10.2289 6.7610 20.5864 1.000
H5 H -6.4375 6.5966 21.2199 1.000
C4 C -8.3160 6.6794 22.0035 1.000
H1 H -9.0586 7.2492 17.6824 1.000
Br1 BR -11.9806 6.7297 20.2963 1.000
C3 C -9.7424 6.7610 21.8257 1.000
Cl1 CL -7.5968 6.7542 23.5296 1.000
H3 H -10.4045 6.8171 22.6809 1.000

Selected Atoms
40 atoms, fractional coordinates
Label Symbol x y z Occ.

H17A H -0.0879 0.5048 0.7966 1.000
H17B H -0.0604 0.4954 0.9265 1.000
Br1 BR 0.8615 0.4894 0.4932 1.000
C3 C 0.5641 0.4940 0.3797 1.000
H3 H 0.6185 0.5023 0.3162 1.000

N1 N 0.3680 0.4860 0.6923 1.000
C1 C 0.5670 0.4870 0.5560 1.000
C5 C 0.3260 0.4810 0.4397 1.000
H5 H 0.1984 0.4698 0.4247 1.000
C6 C 0.3950 0.4920 0.5216 1.000
O1 O 0.6465 0.4860 0.6459 1.000
H1 H 0.5915 0.5658 0.6873 1.000
C7 C 0.3051 0.4790 0.6174 1.000
H7 H 0.1778 0.4631 0.6022 1.000
C8 C 0.2850 0.4900 0.7797 1.000
C14 C 0.1800 0.6630 0.7810 1.000
H14A H 0.0846 0.6576 0.7157 1.000
H14B H 0.2468 0.7958 0.7759 1.000
C15 C 0.1090 0.6750 0.8750 1.000
H15 H 0.0424 0.8098 0.8747 1.000
C17 C 0.0000 0.4980 0.8655 1.000
C16 C 0.2410 0.6680 0.9600 1.000
H16B H 0.3195 0.7903 0.9564 1.000
C12 C 0.1010 0.3090 0.8675 1.000
H12 H 0.0245 0.1830 0.8671 1.000
C11 C 0.2270 0.3030 0.9620 1.000
H11B H 0.2946 0.1685 0.9644 1.000
C10 C 0.3328 0.4780 0.9636 1.000
C13 C 0.1800 0.2930 0.7740 1.000
H13A H 0.0908 0.2874 0.7056 1.000
H13B H 0.2532 0.1628 0.7788 1.000
C9 C 0.4027 0.5040 0.8711 1.000
H9A H 0.4601 0.6460 0.8746 1.000
H9B H 0.4916 0.3920 0.8714 1.000
C2 C 0.6520 0.4940 0.4717 1.000
C4 C 0.3950 0.4820 0.3665 1.000
Cl1 CL 0.2729 0.4930 0.2532 1.000
H16A H 0.1974 0.6827 1.0275 1.000
H11A H 0.1723 0.3060 1.0255 1.000
H10 H 0.4267 0.4739 1.0297 1.000

Unselected Atoms
560 atoms, fractional coordinates
Label Symbol x y z Occ.

H5 H -0.1984 -0.0302 0.5753 1.000
H11A H -0.1723 -0.1940 0.9745 1.000
Cl1 CL -0.2729 -0.0070 0.7468 1.000
Br1 BR -0.1385 0.4894 0.4932 1.000
H5 H -0.1984 0.9698 0.5753 1.000

O1 O -0.3535 0.4860 0.6459 1.000
H1 H -0.4085 0.5658 0.6873 1.000
H14A H -0.0846 0.1576 0.2843 1.000
H15 H -0.0424 0.3098 0.1253 1.000
H16A H -0.1974 0.1826 0.9725 1.000
H12 H -0.0245 0.6830 0.1329 1.000
H11A H -0.1723 0.8060 0.9745 1.000
H13A H -0.0908 0.7874 0.2944 1.000
Cl1 CL -0.2729 0.9930 0.7468 1.000
C15 C -0.1090 0.1750 1.1250 1.000
H15 H -0.0424 0.3098 1.1253 1.000
C16 C -0.2410 0.1680 1.0400 1.000
H16B H -0.3195 0.2903 1.0436 1.000
C12 C -0.1010 0.8090 1.1325 1.000
H12 H -0.0245 0.6830 1.1329 1.000
C11 C -0.2270 0.8030 1.0380 1.000
H11B H -0.2946 0.6685 1.0356 1.000
H16A H -0.1974 1.1826 0.9725 1.000
Br1 BR 0.1385 -0.0106 0.5068 1.000
C3 C 0.4359 -0.0060 0.6203 1.000
N1 N 0.6320 -0.0140 0.3077 1.000
C1 C 0.4330 -0.0130 0.4440 1.000
C5 C 0.6740 -0.0190 0.5603 1.000
H5 H 0.8016 -0.0302 0.5753 1.000
C6 C 0.6050 -0.0080 0.4784 1.000
O1 O 0.3535 -0.0140 0.3541 1.000
C7 C 0.6949 -0.0210 0.3826 1.000
C14 C 0.1800 -0.3370 0.7810 1.000
H14A H 0.0846 -0.3424 0.7157 1.000
H14B H 0.2468 -0.2042 0.7759 1.000
C15 C 0.1090 -0.3250 0.8750 1.000
H15 H 0.0424 -0.1902 0.8747 1.000
C16 C 0.2410 -0.3320 0.9600 1.000
H16B H 0.3195 -0.2097 0.9564 1.000
H10 H 0.5733 -0.0261 0.9703 1.000
C2 C 0.3480 -0.0060 0.5283 1.000
C4 C 0.6050 -0.0180 0.6335 1.000
Cl1 CL 0.7271 -0.0070 0.7468 1.000
C17 C 0.0000 -0.0020 1.1345 1.000
H17B H 0.0604 -0.0046 1.0735 1.000
H16A H 0.1974 -0.3173 1.0275 1.000
C10 C 0.6672 -0.0220 1.0364 1.000
H9B H 0.5084 -0.1079 1.1286 1.000
Br1 BR 0.1385 0.9894 0.5068 1.000
C3 C 0.4359 0.9940 0.6203 1.000
H3 H 0.3815 0.0022 0.6838 1.000

N1 N 0.6320 0.9860 0.3077 1.000
C1 C 0.4330 0.9870 0.4440 1.000
C5 C 0.6740 0.9810 0.5603 1.000
H5 H 0.8016 0.9698 0.5753 1.000
C6 C 0.6050 0.9920 0.4784 1.000
O1 O 0.3535 0.9860 0.3541 1.000
H1 H 0.4085 0.0658 0.3127 1.000
C7 C 0.6949 0.9790 0.3826 1.000
H7 H 0.8222 0.9631 0.3978 1.000
C8 C 0.7150 0.9900 0.2203 1.000
C14 C 0.8200 0.1630 0.2190 1.000
H14A H 0.9154 0.1576 0.2843 1.000
H14B H 0.7532 0.2958 0.2241 1.000
H17A H 0.9121 0.5048 0.7966 1.000
H17A H 0.0879 0.0049 0.2034 1.000
H16A H 0.1974 0.6827 0.0275 1.000
H16A H 0.8026 0.1826 0.9725 1.000
H11A H 0.1723 0.3060 0.0255 1.000
H11A H 0.8277 0.8060 0.9745 1.000
H10 H 0.4267 0.4739 0.0297 1.000
H10 H 0.5733 0.9739 0.9703 1.000
C13 C 0.8200 0.7930 0.2260 1.000
H13A H 0.9092 0.7874 0.2944 1.000
H13B H 0.7468 0.6628 0.2212 1.000
H9A H 0.5399 0.1460 0.1254 1.000
H9B H 0.5084 0.8921 0.1286 1.000
C2 C 0.3480 0.9940 0.5283 1.000
C4 C 0.6050 0.9820 0.6335 1.000
Cl1 CL 0.7271 0.9930 0.7468 1.000
H14B H 0.7532 0.2958 1.2241 1.000
C17 C 0.0000 0.9980 1.1345 1.000
H17A H 0.0879 0.0049 1.2034 1.000
H17B H 0.0604 0.9954 1.0735 1.000
C16 C 0.7590 0.1680 1.0400 1.000
H16B H 0.6805 0.2903 1.0436 1.000
C11 C 0.7730 0.8030 1.0380 1.000
H11B H 0.7054 0.6685 1.0356 1.000
C10 C 0.6672 0.9780 1.0364 1.000
H13B H 0.7468 0.6628 1.2212 1.000
C9 C 0.5973 0.0040 1.1289 1.000
H9A H 0.5399 0.1460 1.1254 1.000
H9B H 0.5084 0.8921 1.1286 1.000
Cl1 CL 0.2729 0.4930 1.2532 1.000
H3 H 0.3815 1.0022 0.6838 1.000
C12 C 0.1010 1.3090 0.8675 1.000
H12 H 0.0245 1.1830 0.8671 1.000

C11 C 0.2270 1.3030 0.9620 1.000
H11B H 0.2946 1.1685 0.9644 1.000
C13 C 0.1800 1.2930 0.7740 1.000
H13A H 0.0908 1.2874 0.7056 1.000
H13B H 0.2532 1.1628 0.7788 1.000
H17A H 0.0879 1.0049 1.2034 1.000
C9 C 0.5973 1.0040 1.1289 1.000
H9A H 0.5399 1.1460 1.1254 1.000
H5 H 1.1984 0.4698 0.4247 1.000
H7 H 1.1778 0.4631 0.6022 1.000
H14A H 1.0846 0.6576 0.7157 1.000
H13A H 1.0908 0.2874 0.7056 1.000
C5 C -0.3260 -0.0190 0.5603 1.000
C11 C -0.2270 -0.1970 1.0380 1.000
C4 C -0.3950 -0.0180 0.6335 1.000
C2 C -0.3480 0.4940 0.4717 1.000
C5 C -0.3260 0.9810 0.5603 1.000
C1 C -0.4330 0.4870 0.5560 1.000
C14 C -0.1800 0.1630 0.2190 1.000
C15 C -0.1090 0.1750 0.1250 1.000
C12 C -0.1010 0.8090 0.1325 1.000
C13 C -0.1800 0.7930 0.2260 1.000
C4 C -0.3950 0.9820 0.6335 1.000
C14 C -0.1800 0.1630 1.2190 1.000
C10 C -0.3328 -0.0220 1.0364 1.000
C13 C -0.1800 0.7930 1.2260 1.000
C10 C -0.3328 0.9780 1.0364 1.000
C16 C -0.2410 1.1680 1.0400 1.000
C8 C 0.7150 -0.0100 0.2203 1.000
H7 H 0.8222 -0.0369 0.3978 1.000
C8 C 0.2850 -0.5100 0.7797 1.000
C17 C 0.0000 -0.5020 0.8655 1.000
C10 C 0.3328 -0.5220 0.9636 1.000
C12 C -0.1010 -0.1910 1.1325 1.000
C11 C 0.7730 -0.1970 1.0380 1.000
H1 H 0.4085 1.0658 0.3127 1.000
C14 C 0.8200 1.1630 0.2190 1.000
C9 C 0.5973 1.0040 0.1289 1.000
C15 C 0.8910 0.1750 0.1250 1.000
C17 C 1.0000 0.4980 0.8655 1.000
C17 C 0.0000 -0.0020 0.1345 1.000
C16 C 0.2410 0.6680 -0.0400 1.000
C11 C 0.2270 0.3030 -0.0380 1.000
C10 C 0.3328 0.4780 -0.0364 1.000
C12 C 0.8990 0.8090 0.1325 1.000
C9 C 0.5973 0.0040 0.1289 1.000

C14 C 0.8200 0.1630 1.2190 1.000
C15 C -0.1090 1.1750 1.1250 1.000
C15 C 0.8910 0.1750 1.1250 1.000
C12 C 0.8990 0.8090 1.1325 1.000
C16 C 0.7590 1.1680 1.0400 1.000
C13 C 0.8200 0.7930 1.2260 1.000
C8 C 0.7150 -0.0100 1.2203 1.000
C4 C 0.3950 0.4820 1.3665 1.000
C17 C 0.0000 1.4980 0.8655 1.000
H11A H 0.1723 1.3060 1.0255 1.000
C10 C 0.3328 1.4780 0.9636 1.000
C8 C 0.2850 1.4900 0.7797 1.000
C8 C 0.7150 0.9900 1.2203 1.000
C5 C 1.3260 0.4810 0.4397 1.000
C7 C 1.3051 0.4790 0.6174 1.000
C14 C 1.1800 0.6630 0.7810 1.000
C13 C 1.1800 0.2930 0.7740 1.000
C6 C -0.3950 -0.0080 0.4784 1.000
H11B H -0.2946 -0.3315 1.0356 1.000
C3 C -0.5641 -0.0060 0.6203 1.000
C3 C -0.4359 0.4940 0.3797 1.000
C6 C -0.3950 0.9920 0.4784 1.000
C6 C -0.6050 0.4920 0.5216 1.000
C8 C -0.2850 -0.0100 0.2203 1.000
H14B H -0.2468 0.2958 0.2241 1.000
C16 C -0.2410 0.1680 0.0400 1.000
C17 C 0.0000 0.9980 0.1345 1.000
C11 C -0.2270 0.8030 0.0380 1.000
C8 C -0.2850 0.9900 0.2203 1.000
H13B H -0.2532 0.6628 0.2212 1.000
C3 C -0.5641 0.9940 0.6203 1.000
C8 C -0.2850 -0.0100 1.2203 1.000
H14A H -0.0846 0.1576 1.2843 1.000
H14B H -0.2468 0.2958 1.2241 1.000
H10 H -0.4267 -0.0261 0.9703 1.000
C9 C -0.4027 0.0040 1.1289 1.000
C8 C -0.2850 0.9900 1.2203 1.000
H13A H -0.0908 0.7874 1.2944 1.000
H13B H -0.2532 0.6628 1.2212 1.000
H10 H -0.4267 0.9739 0.9703 1.000
C9 C -0.4027 1.0040 1.1289 1.000
H16B H -0.3195 1.2903 1.0436 1.000
C13 C 0.8200 -0.2070 0.2260 1.000
N1 N 0.3680 -0.5140 0.6923 1.000
C13 C 0.1800 -0.7070 0.7740 1.000
C9 C 0.4027 -0.4960 0.8711 1.000

H17A H -0.0879 -0.4952 0.7966 1.000
H17B H -0.0604 -0.5046 0.9265 1.000
C12 C 0.1010 -0.6910 0.8675 1.000
C11 C 0.2270 -0.6970 0.9620 1.000
H10 H 0.4267 -0.5261 1.0297 1.000
H12 H -0.0245 -0.3170 1.1329 1.000
C13 C -0.1800 -0.2070 1.2260 1.000
C12 C 0.8990 -0.1910 1.1325 1.000
H11A H 0.8277 -0.1940 0.9745 1.000
H11B H 0.7054 -0.3315 1.0356 1.000
H14A H 0.9154 1.1576 0.2843 1.000
H14B H 0.7532 1.2958 0.2241 1.000
C15 C 0.8910 1.1750 0.1250 1.000
C10 C 0.6672 0.9780 0.0364 1.000
H9A H 0.5399 1.1460 0.1254 1.000
H15 H 0.9576 0.3098 0.1253 1.000
C17 C 1.0000 -0.0020 0.1345 1.000
C16 C 0.7590 0.1680 0.0400 1.000
C15 C 1.1090 0.6750 0.8750 1.000
H17B H 0.9396 0.4954 0.9265 1.000
C12 C 1.1010 0.3090 0.8675 1.000
H17B H 0.0604 -0.0046 0.0735 1.000
C12 C -0.1010 -0.1910 0.1325 1.000
C15 C 0.1090 0.6750 -0.1250 1.000
H16B H 0.3195 0.7903 -0.0436 1.000
C12 C 0.1010 0.3090 -0.1325 1.000
H11B H 0.2946 0.1685 -0.0356 1.000
C9 C 0.4027 0.5040 -0.1289 1.000
C17 C 1.0000 0.9980 0.1345 1.000
H12 H 0.9755 0.6830 0.1329 1.000
C11 C 0.7730 0.8030 0.0380 1.000
C10 C 0.6672 -0.0220 0.0364 1.000
H9B H 0.5084 -0.1079 0.1286 1.000
H14A H 0.9154 0.1576 1.2843 1.000
C14 C -0.1800 1.1630 1.2190 1.000
H15 H -0.0424 1.3098 1.1253 1.000
H15 H 0.9576 0.3098 1.1253 1.000
C17 C 1.0000 -0.0020 1.1345 1.000
C17 C 1.0000 0.9980 1.1345 1.000
H12 H 0.9755 0.6830 1.1329 1.000
C15 C 0.8910 1.1750 1.1250 1.000
H16A H 0.8026 1.1826 0.9725 1.000
H16B H 0.6805 1.2903 1.0436 1.000
H13A H 0.9092 0.7874 1.2944 1.000
N1 N 0.6320 -0.0140 1.3077 1.000
C13 C 0.8200 -0.2070 1.2260 1.000

C3 C 0.5641 0.4940 1.3797 1.000
C5 C 0.3260 0.4810 1.4397 1.000
C15 C 0.1090 1.6750 0.8750 1.000
H17A H -0.0879 1.5048 0.7966 1.000
H17B H -0.0604 1.4954 0.9265 1.000
C16 C 0.2410 1.6680 0.9600 1.000
H10 H 0.4267 1.4739 1.0297 1.000
C9 C 0.4027 1.5040 0.8711 1.000
N1 N 0.3680 1.4860 0.6923 1.000
C14 C 0.1800 1.6630 0.7810 1.000
N1 N 0.6320 0.9860 1.3077 1.000
C14 C 0.8200 1.1630 1.2190 1.000
C6 C 1.3950 0.4920 0.5216 1.000
C4 C 1.3950 0.4820 0.3665 1.000
N1 N 1.3680 0.4860 0.6923 1.000
C8 C 1.2850 0.4900 0.7797 1.000
H14B H 1.2468 0.7958 0.7759 1.000
H13B H 1.2532 0.1628 0.7788 1.000
C1 C -0.5670 -0.0130 0.4440 1.000
C7 C -0.3051 -0.0210 0.3826 1.000
H3 H -0.6185 0.0022 0.6838 1.000
C2 C -0.6520 -0.0060 0.5283 1.000
H3 H -0.3815 0.5023 0.3162 1.000
C4 C -0.6050 0.4820 0.3665 1.000
C1 C -0.5670 0.9870 0.4440 1.000
C7 C -0.3051 0.9790 0.3826 1.000
C5 C -0.6740 0.4810 0.4397 1.000
C7 C -0.6949 0.4790 0.6174 1.000
N1 N -0.3680 -0.0140 0.3077 1.000
C13 C -0.1800 -0.2070 0.2260 1.000
C9 C -0.4027 0.0040 0.1289 1.000
H16A H -0.1974 0.1826 -0.0275 1.000
H16B H -0.3195 0.2903 0.0436 1.000
C10 C -0.3328 -0.0220 0.0364 1.000
C15 C -0.1090 1.1750 0.1250 1.000
H17A H 0.0879 1.0049 0.2034 1.000
H17B H 0.0604 0.9954 0.0735 1.000
H11A H -0.1723 0.8060 -0.0255 1.000
H11B H -0.2946 0.6685 0.0356 1.000
C10 C -0.3328 0.9780 0.0364 1.000
N1 N -0.3680 0.9860 0.3077 1.000
C14 C -0.1800 1.1630 0.2190 1.000
C9 C -0.4027 1.0040 0.1289 1.000
H3 H -0.6185 1.0022 0.6838 1.000
C2 C -0.6520 0.9940 0.5283 1.000
N1 N -0.3680 -0.0140 1.3077 1.000

H9A H -0.4601 0.1460 1.1254 1.000
H9B H -0.4916 -0.1079 1.1286 1.000
N1 N -0.3680 0.9860 1.3077 1.000
H9A H -0.4601 1.1460 1.1254 1.000
H9B H -0.4916 0.8921 1.1286 1.000
C12 C 0.8990 -0.1910 0.1325 1.000
H13A H 0.9092 -0.2126 0.2944 1.000
H13B H 0.7468 -0.3372 0.2212 1.000
C7 C 0.3051 -0.5210 0.6174 1.000
H13A H 0.0908 -0.7126 0.7056 1.000
H13B H 0.2532 -0.8372 0.7788 1.000
H9A H 0.4601 -0.3540 0.8746 1.000
H9B H 0.4916 -0.6079 0.8714 1.000
H12 H 0.0245 -0.8170 0.8671 1.000
H11A H 0.1723 -0.6940 1.0255 1.000
H11B H 0.2946 -0.8315 0.9644 1.000
H13A H -0.0908 -0.2126 1.2944 1.000
H13B H -0.2532 -0.3372 1.2212 1.000
H12 H 0.9755 -0.3170 1.1329 1.000
H15 H 0.9576 1.3098 0.1253 1.000
C16 C 0.7590 1.1680 0.0400 1.000
H10 H 0.5733 0.9739 -0.0297 1.000
H17A H 1.0879 0.0049 0.2034 1.000
H17B H 1.0603 -0.0046 0.0735 1.000
H16A H 0.8026 0.1826 -0.0275 1.000
H16B H 0.6805 0.2903 0.0436 1.000
H15 H 1.0424 0.8098 0.8747 1.000
C16 C 1.2410 0.6680 0.9600 1.000
H12 H 1.0245 0.1830 0.8671 1.000
C11 C 1.2270 0.3030 0.9620 1.000
H12 H -0.0245 -0.3170 0.1329 1.000
C11 C -0.2270 -0.1970 0.0380 1.000
C14 C 0.1800 0.6630 -0.2190 1.000
H15 H 0.0424 0.8098 -0.1253 1.000
C17 C 0.0000 0.4980 -0.1345 1.000
H12 H 0.0245 0.1830 -0.1329 1.000
C13 C 0.1800 0.2930 -0.2260 1.000
C8 C 0.2850 0.4900 -0.2203 1.000
H9A H 0.4601 0.6460 -0.1254 1.000
H9B H 0.4916 0.3920 -0.1286 1.000
H17A H 1.0879 1.0049 0.2034 1.000
H17B H 1.0603 0.9954 0.0735 1.000
H11A H 0.8277 0.8060 -0.0255 1.000
H11B H 0.7054 0.6685 0.0356 1.000
C11 C 0.7730 -0.1970 0.0380 1.000
H10 H 0.5733 -0.0261 -0.0297 1.000

H14A H -0.0846 1.1576 1.2843 1.000
H14B H -0.2468 1.2958 1.2241 1.000
H17A H 1.0879 0.0049 1.2034 1.000
H17B H 1.0603 -0.0046 1.0735 1.000
H17A H 1.0879 1.0049 1.2034 1.000
H17B H 1.0603 0.9954 1.0735 1.000
H15 H 0.9576 1.3098 1.1253 1.000
C7 C 0.6949 -0.0210 1.3826 1.000
H13A H 0.9092 -0.2126 1.2944 1.000
H13B H 0.7468 -0.3372 1.2212 1.000
H3 H 0.6185 0.5023 1.3162 1.000
C2 C 0.6520 0.4940 1.4717 1.000
H5 H 0.1984 0.4698 1.4247 1.000
C6 C 0.3950 0.4920 1.5216 1.000
H15 H 0.0424 1.8098 0.8747 1.000
H16A H 0.1974 1.6827 1.0275 1.000
H16B H 0.3195 1.7903 0.9564 1.000
H9A H 0.4601 1.6460 0.8746 1.000
H9B H 0.4916 1.3921 0.8714 1.000
C7 C 0.3051 1.4790 0.6174 1.000
H14A H 0.0846 1.6576 0.7157 1.000
H14B H 0.2468 1.7958 0.7759 1.000
C7 C 0.6949 0.9790 1.3826 1.000
H14A H 0.9154 1.1576 1.2843 1.000
H14B H 0.7532 1.2958 1.2241 1.000
C1 C 1.5670 0.4870 0.5560 1.000
C3 C 1.5641 0.4940 0.3797 1.000
Cl1 CL 1.2729 0.4930 0.2532 1.000
C9 C 1.4027 0.5040 0.8711 1.000
O1 O -0.6465 -0.0140 0.3541 1.000
H7 H -0.1778 -0.0369 0.3978 1.000
Br1 BR -0.8615 -0.0106 0.5068 1.000
Cl1 CL -0.7271 0.4930 0.2532 1.000
O1 O -0.6465 0.9860 0.3541 1.000
H7 H -0.1778 0.9631 0.3978 1.000
H5 H -0.8016 0.4698 0.4247 1.000
N1 N -0.6320 0.4860 0.6923 1.000
H7 H -0.8222 0.4631 0.6022 1.000
H13A H -0.0908 -0.2126 0.2944 1.000
H13B H -0.2532 -0.3372 0.2212 1.000
H9A H -0.4601 0.1460 0.1254 1.000
H9B H -0.4916 -0.1079 0.1286 1.000
H10 H -0.4267 -0.0261 -0.0297 1.000
H15 H -0.0424 1.3098 0.1253 1.000
C16 C -0.2410 1.1680 0.0400 1.000
H10 H -0.4267 0.9739 -0.0297 1.000

H14A H -0.0846 1.1576 0.2843 1.000
H14B H -0.2468 1.2958 0.2241 1.000
H9A H -0.4601 1.1460 0.1254 1.000
H9B H -0.4916 0.8921 0.1286 1.000
Br1 BR -0.8615 0.9894 0.5068 1.000
C7 C -0.3051 -0.0210 1.3826 1.000
C7 C -0.3051 0.9790 1.3826 1.000
H12 H 0.9755 -0.3170 0.1329 1.000
C6 C 0.3950 -0.5080 0.5216 1.000
H7 H 0.1778 -0.5369 0.6022 1.000
H16A H 0.8026 1.1826 -0.0275 1.000
H16B H 0.6805 1.2903 0.0436 1.000
H16A H 1.1974 0.6827 1.0275 1.000
H16B H 1.3195 0.7903 0.9564 1.000
C10 C 1.3328 0.4780 0.9636 1.000
H11A H 1.1723 0.3060 1.0255 1.000
H11B H 1.2946 0.1685 0.9644 1.000
H11A H -0.1723 -0.1940 -0.0255 1.000
H11B H -0.2946 -0.3315 0.0356 1.000
H14A H 0.0846 0.6576 -0.2843 1.000
H14B H 0.2468 0.7958 -0.2241 1.000
H17A H -0.0879 0.5048 -0.2034 1.000
H17B H -0.0604 0.4954 -0.0735 1.000
H13A H 0.0908 0.2874 -0.2944 1.000
H13B H 0.2532 0.1628 -0.2212 1.000
N1 N 0.3680 0.4860 -0.3077 1.000
H11A H 0.8277 -0.1940 -0.0255 1.000
H11B H 0.7054 -0.3315 0.0356 1.000
C6 C 0.6050 -0.0080 1.4784 1.000
H7 H 0.8222 -0.0369 1.3978 1.000
Br1 BR 0.8615 0.4894 1.4932 1.000
C1 C 0.5670 0.4870 1.5560 1.000
C7 C 0.3051 0.4790 1.6174 1.000
C6 C 0.3950 1.4920 0.5216 1.000
H7 H 0.1778 1.4631 0.6022 1.000
C6 C 0.6050 0.9920 1.4784 1.000
H7 H 0.8222 0.9631 1.3978 1.000
O1 O 1.6465 0.4860 0.6459 1.000
C2 C 1.6520 0.4940 0.4717 1.000
H3 H 1.6185 0.5023 0.3162 1.000
H9A H 1.4601 0.6460 0.8746 1.000
H9B H 1.4916 0.3920 0.8714 1.000
H1 H -0.5915 0.0658 0.3127 1.000
H1 H -0.5915 1.0658 0.3127 1.000
C8 C -0.7150 0.4900 0.7797 1.000
H16A H -0.1974 1.1826 -0.0275 1.000

H16B H -0.3195 1.2903 0.0436 1.000
C6 C -0.3950 -0.0080 1.4784 1.000
H7 H -0.1778 -0.0369 1.3978 1.000
C6 C -0.3950 0.9920 1.4784 1.000
H7 H -0.1778 0.9631 1.3978 1.000
C1 C 0.5670 -0.5130 0.5560 1.000
C5 C 0.3260 -0.5190 0.4397 1.000
H10 H 1.4267 0.4739 1.0297 1.000
C7 C 0.3051 0.4790 -0.3826 1.000
C1 C 0.4330 -0.0130 1.4440 1.000
C5 C 0.6740 -0.0190 1.5603 1.000
O1 O 0.6465 0.4860 1.6459 1.000
N1 N 0.3680 0.4860 1.6923 1.000
H7 H 0.1778 0.4631 1.6022 1.000
C1 C 0.5670 1.4870 0.5560 1.000
C5 C 0.3260 1.4810 0.4397 1.000
C1 C 0.4330 0.9870 1.4440 1.000
C5 C 0.6740 0.9810 1.5603 1.000
H1 H 1.5915 0.5658 0.6873 1.000
Br1 BR 1.8615 0.4894 0.4932 1.000
C14 C -0.8200 0.6630 0.7810 1.000
C13 C -0.8200 0.2930 0.7740 1.000
C9 C -0.5973 0.5040 0.8711 1.000
C1 C -0.5670 -0.0130 1.4440 1.000
C5 C -0.3260 -0.0190 1.5603 1.000
C1 C -0.5670 0.9870 1.4440 1.000
C5 C -0.3260 0.9810 1.5603 1.000
O1 O 0.6465 -0.5140 0.6459 1.000
C2 C 0.6520 -0.5060 0.4717 1.000
H5 H 0.1984 -0.5302 0.4247 1.000
C4 C 0.3950 -0.5180 0.3665 1.000
C6 C 0.3950 0.4920 -0.4784 1.000
H7 H 0.1778 0.4631 -0.3978 1.000
O1 O 0.3535 -0.0140 1.3541 1.000
C2 C 0.3480 -0.0060 1.5283 1.000
H5 H 0.8016 -0.0302 1.5753 1.000
C4 C 0.6050 -0.0180 1.6335 1.000
H1 H 0.5915 0.5658 1.6873 1.000
C8 C 0.2850 0.4900 1.7797 1.000
O1 O 0.6465 1.4860 0.6459 1.000
C2 C 0.6520 1.4940 0.4717 1.000
H5 H 0.1984 1.4698 0.4247 1.000
C4 C 0.3950 1.4820 0.3665 1.000
O1 O 0.3535 0.9860 1.3541 1.000
C2 C 0.3480 0.9940 1.5283 1.000
H5 H 0.8016 0.9698 1.5753 1.000

C4 C 0.6050 0.9820 1.6335 1.000
H14A H -0.9154 0.6576 0.7157 1.000
H14B H -0.7532 0.7958 0.7759 1.000
C15 C -0.8910 0.6750 0.8750 1.000
C12 C -0.8990 0.3090 0.8675 1.000
H13A H -0.9092 0.2874 0.7056 1.000
H13B H -0.7468 0.1628 0.7788 1.000
C10 C -0.6672 0.4780 0.9636 1.000
H9A H -0.5399 0.6460 0.8746 1.000
H9B H -0.5084 0.3920 0.8714 1.000
O1 O -0.6465 -0.0140 1.3541 1.000
C2 C -0.6520 -0.0060 1.5283 1.000
H5 H -0.1984 -0.0302 1.5753 1.000
C4 C -0.3950 -0.0180 1.6335 1.000
O1 O -0.6465 0.9860 1.3541 1.000
C2 C -0.6520 0.9940 1.5283 1.000
H5 H -0.1984 0.9698 1.5753 1.000
C4 C -0.3950 0.9820 1.6335 1.000
H1 H 0.5915 -0.4342 0.6873 1.000
Br1 BR 0.8615 -0.5106 0.4932 1.000
C3 C 0.5641 -0.5060 0.3797 1.000
Cl1 CL 0.2729 -0.5070 0.2532 1.000
C1 C 0.5670 0.4870 -0.4440 1.000
C5 C 0.3260 0.4810 -0.5603 1.000
H1 H 0.4085 0.0658 1.3127 1.000
Br1 BR 0.1385 -0.0106 1.5068 1.000
C3 C 0.4359 -0.0060 1.6203 1.000
Cl1 CL 0.7271 -0.0070 1.7468 1.000
C14 C 0.1800 0.6630 1.7810 1.000
C13 C 0.1800 0.2930 1.7740 1.000
C9 C 0.4027 0.5040 1.8711 1.000
H1 H 0.5915 1.5658 0.6873 1.000
Br1 BR 0.8615 1.4894 0.4932 1.000
C3 C 0.5641 1.4940 0.3797 1.000
Cl1 CL 0.2729 1.4930 0.2532 1.000
H1 H 0.4085 1.0658 1.3127 1.000
Br1 BR 0.1385 0.9894 1.5068 1.000
C3 C 0.4359 0.9940 1.6203 1.000
Cl1 CL 0.7271 0.9930 1.7468 1.000
H15 H -0.9576 0.8098 0.8747 1.000
C17 C -1.0000 0.4980 0.8655 1.000
C16 C -0.7590 0.6680 0.9600 1.000
H12 H -0.9755 0.1830 0.8671 1.000
C11 C -0.7730 0.3030 0.9620 1.000
H10 H -0.5733 0.4739 1.0297 1.000
H1 H -0.5915 0.0658 1.3127 1.000

Br1 BR -0.8615 -0.0106 1.5068 1.000
C3 C -0.5641 -0.0060 1.6203 1.000
Cl1 CL -0.2729 -0.0070 1.7468 1.000
H1 H -0.5915 1.0658 1.3127 1.000
Br1 BR -0.8615 0.9894 1.5068 1.000
C3 C -0.5641 0.9940 1.6203 1.000
Cl1 CL -0.2729 0.9930 1.7468 1.000
H3 H 0.6185 -0.4977 0.3162 1.000
O1 O 0.6465 0.4860 -0.3541 1.000
C2 C 0.6520 0.4940 -0.5283 1.000
H5 H 0.1984 0.4698 -0.5753 1.000
C4 C 0.3950 0.4820 -0.6335 1.000
H3 H 0.3815 0.0022 1.6838 1.000
H14A H 0.0846 0.6576 1.7157 1.000
H14B H 0.2468 0.7958 1.7759 1.000
C15 C 0.1090 0.6750 1.8750 1.000
C12 C 0.1010 0.3090 1.8675 1.000
H13A H 0.0908 0.2874 1.7056 1.000
H13B H 0.2532 0.1628 1.7788 1.000
C10 C 0.3328 0.4780 1.9636 1.000
H9A H 0.4601 0.6460 1.8746 1.000
H9B H 0.4916 0.3920 1.8714 1.000
H3 H 0.6185 1.5022 0.3162 1.000
H3 H 0.3815 1.0022 1.6838 1.000
H17A H -1.0879 0.5048 0.7966 1.000
H17B H -1.0603 0.4954 0.9265 1.000
H16A H -0.8026 0.6827 1.0275 1.000
H16B H -0.6805 0.7903 0.9564 1.000
H11A H -0.8277 0.3060 1.0255 1.000
H11B H -0.7054 0.1685 0.9644 1.000
H3 H -0.6185 0.0022 1.6838 1.000
H3 H -0.6185 1.0022 1.6838 1.000
H1 H 0.5915 0.5658 -0.3127 1.000
Br1 BR 0.8615 0.4894 -0.5068 1.000
C3 C 0.5641 0.4940 -0.6203 1.000
Cl1 CL 0.2729 0.4930 -0.7468 1.000
H15 H 0.0424 0.8098 1.8747 1.000
C17 C 0.0000 0.4980 1.8655 1.000
C16 C 0.2410 0.6680 1.9600 1.000
H12 H 0.0245 0.1830 1.8671 1.000
C11 C 0.2270 0.3030 1.9620 1.000
H10 H 0.4267 0.4739 2.0297 1.000
H3 H 0.6185 0.5023 -0.6838 1.000
H17A H -0.0879 0.5048 1.7966 1.000
H17B H -0.0604 0.4954 1.9265 1.000
H16A H 0.1974 0.6827 2.0275 1.000

H16B H 0.3195 0.7903 1.9564 1.000
H11A H 0.1723 0.3060 2.0255 1.000
H11B H 0.2946 0.1685 1.9644 1.000

All Atoms [15 molecules]
600 atoms, fractional coordinates
Label Symbol x y z Occ.

[-x, y+1/2, -z]
H17A H 0.9121 0.5048 0.7966 1.000
C17 C 1.0000 0.4980 0.8655 1.000
C15 C 1.1090 0.6750 0.8750 1.000
H17B H 0.9396 0.4954 0.9265 1.000
C12 C 1.1010 0.3090 0.8675 1.000
C14 C 1.1800 0.6630 0.7810 1.000
H15 H 1.0424 0.8098 0.8747 1.000
C16 C 1.2410 0.6680 0.9600 1.000
C13 C 1.1800 0.2930 0.7740 1.000
H12 H 1.0245 0.1830 0.8671 1.000
C11 C 1.2270 0.3030 0.9620 1.000
H14A H 1.0846 0.6576 0.7157 1.000
C8 C 1.2850 0.4900 0.7797 1.000
H14B H 1.2468 0.7958 0.7759 1.000
H16A H 1.1974 0.6827 1.0275 1.000
H16B H 1.3195 0.7903 0.9564 1.000
C10 C 1.3328 0.4780 0.9636 1.000
H13A H 1.0908 0.2874 0.7056 1.000
H13B H 1.2532 0.1628 0.7788 1.000
H11A H 1.1723 0.3060 1.0255 1.000
H11B H 1.2946 0.1685 0.9644 1.000
N1 N 1.3680 0.4860 0.6923 1.000
C9 C 1.4027 0.5040 0.8711 1.000
H10 H 1.4267 0.4739 1.0297 1.000
C7 C 1.3051 0.4790 0.6174 1.000
H9A H 1.4601 0.6460 0.8746 1.000
H9B H 1.4916 0.3920 0.8714 1.000
H7 H 1.1778 0.4631 0.6022 1.000
C6 C 1.3950 0.4920 0.5216 1.000
C5 C 1.3260 0.4810 0.4397 1.000
C1 C 1.5670 0.4870 0.5560 1.000
H5 H 1.1984 0.4698 0.4247 1.000
C4 C 1.3950 0.4820 0.3665 1.000
O1 O 1.6465 0.4860 0.6459 1.000
C2 C 1.6520 0.4940 0.4717 1.000
C3 C 1.5641 0.4940 0.3797 1.000

Cl1 CL 1.2729 0.4930 0.2532 1.000
H1 H 1.5915 0.5658 0.6873 1.000
Br1 BR 1.8615 0.4894 0.4932 1.000
H3 H 1.6185 0.5023 0.3162 1.000

[-x, y+1/2, -z]
H16A H 0.1974 0.6827 0.0275 1.000
C16 C 0.2410 0.6680 -0.0400 1.000
C10 C 0.3328 0.4780 -0.0364 1.000
C15 C 0.1090 0.6750 -0.1250 1.000
H16B H 0.3195 0.7903 -0.0436 1.000
H10 H 0.4267 0.4739 0.0297 1.000
C11 C 0.2270 0.3030 -0.0380 1.000
C9 C 0.4027 0.5040 -0.1289 1.000
C14 C 0.1800 0.6630 -0.2190 1.000
H15 H 0.0424 0.8098 -0.1253 1.000
C17 C 0.0000 0.4980 -0.1345 1.000
H11A H 0.1723 0.3060 0.0255 1.000
C12 C 0.1010 0.3090 -0.1325 1.000
H11B H 0.2946 0.1685 -0.0356 1.000
C8 C 0.2850 0.4900 -0.2203 1.000
H9A H 0.4601 0.6460 -0.1254 1.000
H9B H 0.4916 0.3920 -0.1286 1.000
H14A H 0.0846 0.6576 -0.2843 1.000
H14B H 0.2468 0.7958 -0.2241 1.000
H17A H -0.0879 0.5048 -0.2034 1.000
H17B H -0.0604 0.4954 -0.0735 1.000
H12 H 0.0245 0.1830 -0.1329 1.000
C13 C 0.1800 0.2930 -0.2260 1.000
N1 N 0.3680 0.4860 -0.3077 1.000
H13A H 0.0908 0.2874 -0.2944 1.000
H13B H 0.2532 0.1628 -0.2212 1.000
C7 C 0.3051 0.4790 -0.3826 1.000
C6 C 0.3950 0.4920 -0.4784 1.000
H7 H 0.1778 0.4631 -0.3978 1.000
C1 C 0.5670 0.4870 -0.4440 1.000
C5 C 0.3260 0.4810 -0.5603 1.000
O1 O 0.6465 0.4860 -0.3541 1.000
C2 C 0.6520 0.4940 -0.5283 1.000
H5 H 0.1984 0.4698 -0.5753 1.000
C4 C 0.3950 0.4820 -0.6335 1.000
H1 H 0.5915 0.5658 -0.3127 1.000
Br1 BR 0.8615 0.4894 -0.5068 1.000
C3 C 0.5641 0.4940 -0.6203 1.000
Cl1 CL 0.2729 0.4930 -0.7468 1.000
H3 H 0.6185 0.5023 -0.6838 1.000

[x, y, z]
H10 H 0.5733 -0.0261 0.9703 1.000
C10 C 0.6672 -0.0220 1.0364 1.000
C16 C 0.7590 0.1680 1.0400 1.000
C9 C 0.5973 0.0040 1.1289 1.000
C11 C 0.7730 -0.1970 1.0380 1.000
H16A H 0.8026 0.1826 0.9725 1.000
H16B H 0.6805 0.2903 1.0436 1.000
C15 C 0.8910 0.1750 1.1250 1.000
H9B H 0.5084 -0.1079 1.1286 1.000
H9A H 0.5399 0.1460 1.1254 1.000
C8 C 0.7150 -0.0100 1.2203 1.000
C12 C 0.8990 -0.1910 1.1325 1.000
H11A H 0.8277 -0.1940 0.9745 1.000
H11B H 0.7054 -0.3315 1.0356 1.000
C14 C 0.8200 0.1630 1.2190 1.000
H15 H 0.9576 0.3098 1.1253 1.000
C17 C 1.0000 -0.0020 1.1345 1.000
N1 N 0.6320 -0.0140 1.3077 1.000
C13 C 0.8200 -0.2070 1.2260 1.000
H12 H 0.9755 -0.3170 1.1329 1.000
H14B H 0.7532 0.2958 1.2241 1.000
H14A H 0.9154 0.1576 1.2843 1.000
H17A H 1.0879 0.0049 1.2034 1.000
H17B H 1.0603 -0.0046 1.0735 1.000
C7 C 0.6949 -0.0210 1.3826 1.000
H13A H 0.9092 -0.2126 1.2944 1.000
H13B H 0.7468 -0.3372 1.2212 1.000
C6 C 0.6050 -0.0080 1.4784 1.000
H7 H 0.8222 -0.0369 1.3978 1.000
C1 C 0.4330 -0.0130 1.4440 1.000
C5 C 0.6740 -0.0190 1.5603 1.000
O1 O 0.3535 -0.0140 1.3541 1.000
C2 C 0.3480 -0.0060 1.5283 1.000
H5 H 0.8016 -0.0302 1.5753 1.000
C4 C 0.6050 -0.0180 1.6335 1.000
H1 H 0.4085 0.0658 1.3127 1.000
Br1 BR 0.1385 -0.0106 1.5068 1.000
C3 C 0.4359 -0.0060 1.6203 1.000
Cl1 CL 0.7271 -0.0070 1.7468 1.000
H3 H 0.3815 0.0022 1.6838 1.000

[x, y, z]
Br1 BR 0.1385 0.9894 0.5068 1.000
C2 C 0.3480 0.9940 0.5283 1.000

C3 C 0.4359 0.9940 0.6203 1.000
C1 C 0.4330 0.9870 0.4440 1.000
C4 C 0.6050 0.9820 0.6335 1.000
H3 H 0.3815 1.0022 0.6838 1.000
C6 C 0.6050 0.9920 0.4784 1.000
O1 O 0.3535 0.9860 0.3541 1.000
C5 C 0.6740 0.9810 0.5603 1.000
Cl1 CL 0.7271 0.9930 0.7468 1.000
C7 C 0.6949 0.9790 0.3826 1.000
H1 H 0.4085 1.0658 0.3127 1.000
H5 H 0.8016 0.9698 0.5753 1.000
N1 N 0.6320 0.9860 0.3077 1.000
H7 H 0.8222 0.9631 0.3978 1.000
C8 C 0.7150 0.9900 0.2203 1.000
C13 C 0.8200 0.7930 0.2260 1.000
C14 C 0.8200 1.1630 0.2190 1.000
C9 C 0.5973 1.0040 0.1289 1.000
H13A H 0.9092 0.7874 0.2944 1.000
H13B H 0.7468 0.6628 0.2212 1.000
C12 C 0.8990 0.8090 0.1325 1.000
H14A H 0.9154 1.1576 0.2843 1.000
H14B H 0.7532 1.2958 0.2241 1.000
C15 C 0.8910 1.1750 0.1250 1.000
H9B H 0.5084 0.8921 0.1286 1.000
C10 C 0.6672 0.9780 0.0364 1.000
H9A H 0.5399 1.1460 0.1254 1.000
C17 C 1.0000 0.9980 0.1345 1.000
H12 H 0.9755 0.6830 0.1329 1.000
C11 C 0.7730 0.8030 0.0380 1.000
H15 H 0.9576 1.3098 0.1253 1.000
C16 C 0.7590 1.1680 0.0400 1.000
H10 H 0.5733 0.9739 -0.0297 1.000
H17A H 1.0879 1.0049 0.2034 1.000
H17B H 1.0603 0.9954 0.0735 1.000
H11A H 0.8277 0.8060 -0.0255 1.000
H11B H 0.7054 0.6685 0.0356 1.000
H16A H 0.8026 1.1826 -0.0275 1.000
H16B H 0.6805 1.2903 0.0436 1.000

[-x, y+1/2, -z]
C12 C 0.1010 1.3090 0.8675 1.000
H12 H 0.0245 1.1830 0.8671 1.000
C11 C 0.2270 1.3030 0.9620 1.000
C13 C 0.1800 1.2930 0.7740 1.000
C17 C 0.0000 1.4980 0.8655 1.000
H11B H 0.2946 1.1685 0.9644 1.000

H11A H 0.1723 1.3060 1.0255 1.000
C10 C 0.3328 1.4780 0.9636 1.000
H13A H 0.0908 1.2874 0.7056 1.000
H13B H 0.2532 1.1628 0.7788 1.000
C8 C 0.2850 1.4900 0.7797 1.000
C15 C 0.1090 1.6750 0.8750 1.000
H17A H -0.0879 1.5048 0.7966 1.000
H17B H -0.0604 1.4954 0.9265 1.000
C16 C 0.2410 1.6680 0.9600 1.000
H10 H 0.4267 1.4739 1.0297 1.000
C9 C 0.4027 1.5040 0.8711 1.000
N1 N 0.3680 1.4860 0.6923 1.000
C14 C 0.1800 1.6630 0.7810 1.000
H15 H 0.0424 1.8098 0.8747 1.000
H16A H 0.1974 1.6827 1.0275 1.000
H16B H 0.3195 1.7903 0.9564 1.000
H9A H 0.4601 1.6460 0.8746 1.000
H9B H 0.4916 1.3921 0.8714 1.000
C7 C 0.3051 1.4790 0.6174 1.000
H14A H 0.0846 1.6576 0.7157 1.000
H14B H 0.2468 1.7958 0.7759 1.000
C6 C 0.3950 1.4920 0.5216 1.000
H7 H 0.1778 1.4631 0.6022 1.000
C1 C 0.5670 1.4870 0.5560 1.000
C5 C 0.3260 1.4810 0.4397 1.000
O1 O 0.6465 1.4860 0.6459 1.000
C2 C 0.6520 1.4940 0.4717 1.000
H5 H 0.1984 1.4698 0.4247 1.000
C4 C 0.3950 1.4820 0.3665 1.000
H1 H 0.5915 1.5658 0.6873 1.000
Br1 BR 0.8615 1.4894 0.4932 1.000
C3 C 0.5641 1.4940 0.3797 1.000
Cl1 CL 0.2729 1.4930 0.2532 1.000
H3 H 0.6185 1.5022 0.3162 1.000

[x, y, z]
H11A H 0.8277 0.8060 0.9745 1.000
C11 C 0.7730 0.8030 1.0380 1.000
H11B H 0.7054 0.6685 1.0356 1.000
C10 C 0.6672 0.9780 1.0364 1.000
C12 C 0.8990 0.8090 1.1325 1.000
H10 H 0.5733 0.9739 0.9703 1.000
C9 C 0.5973 1.0040 1.1289 1.000
C16 C 0.7590 1.1680 1.0400 1.000
C13 C 0.8200 0.7930 1.2260 1.000
C17 C 1.0000 0.9980 1.1345 1.000

H12 H 0.9755 0.6830 1.1329 1.000
H9B H 0.5084 0.8921 1.1286 1.000
H9A H 0.5399 1.1460 1.1254 1.000
C8 C 0.7150 0.9900 1.2203 1.000
C15 C 0.8910 1.1750 1.1250 1.000
H16A H 0.8026 1.1826 0.9725 1.000
H16B H 0.6805 1.2903 1.0436 1.000
H13B H 0.7468 0.6628 1.2212 1.000
H13A H 0.9092 0.7874 1.2944 1.000
H17A H 1.0879 1.0049 1.2034 1.000
H17B H 1.0603 0.9954 1.0735 1.000
N1 N 0.6320 0.9860 1.3077 1.000
C14 C 0.8200 1.1630 1.2190 1.000
H15 H 0.9576 1.3098 1.1253 1.000
C7 C 0.6949 0.9790 1.3826 1.000
H14A H 0.9154 1.1576 1.2843 1.000
H14B H 0.7532 1.2958 1.2241 1.000
C6 C 0.6050 0.9920 1.4784 1.000
H7 H 0.8222 0.9631 1.3978 1.000
C1 C 0.4330 0.9870 1.4440 1.000
C5 C 0.6740 0.9810 1.5603 1.000
O1 O 0.3535 0.9860 1.3541 1.000
C2 C 0.3480 0.9940 1.5283 1.000
H5 H 0.8016 0.9698 1.5753 1.000
C4 C 0.6050 0.9820 1.6335 1.000
H1 H 0.4085 1.0658 1.3127 1.000
Br1 BR 0.1385 0.9894 1.5068 1.000
C3 C 0.4359 0.9940 1.6203 1.000
Cl1 CL 0.7271 0.9930 1.7468 1.000
H3 H 0.3815 1.0022 1.6838 1.000

[-x, y+1/2, -z]
Cl1 CL 0.2729 0.4930 1.2532 1.000
C4 C 0.3950 0.4820 1.3665 1.000
C3 C 0.5641 0.4940 1.3797 1.000
C5 C 0.3260 0.4810 1.4397 1.000
H3 H 0.6185 0.5023 1.3162 1.000
C2 C 0.6520 0.4940 1.4717 1.000
H5 H 0.1984 0.4698 1.4247 1.000
C6 C 0.3950 0.4920 1.5216 1.000
Br1 BR 0.8615 0.4894 1.4932 1.000
C1 C 0.5670 0.4870 1.5560 1.000
C7 C 0.3051 0.4790 1.6174 1.000
O1 O 0.6465 0.4860 1.6459 1.000
N1 N 0.3680 0.4860 1.6923 1.000
H7 H 0.1778 0.4631 1.6022 1.000

H1 H 0.5915 0.5658 1.6873 1.000
C8 C 0.2850 0.4900 1.7797 1.000
C14 C 0.1800 0.6630 1.7810 1.000
C13 C 0.1800 0.2930 1.7740 1.000
C9 C 0.4027 0.5040 1.8711 1.000
H14A H 0.0846 0.6576 1.7157 1.000
H14B H 0.2468 0.7958 1.7759 1.000
C15 C 0.1090 0.6750 1.8750 1.000
C12 C 0.1010 0.3090 1.8675 1.000
H13A H 0.0908 0.2874 1.7056 1.000
H13B H 0.2532 0.1628 1.7788 1.000
C10 C 0.3328 0.4780 1.9636 1.000
H9A H 0.4601 0.6460 1.8746 1.000
H9B H 0.4916 0.3920 1.8714 1.000
H15 H 0.0424 0.8098 1.8747 1.000
C17 C 0.0000 0.4980 1.8655 1.000
C16 C 0.2410 0.6680 1.9600 1.000
H12 H 0.0245 0.1830 1.8671 1.000
C11 C 0.2270 0.3030 1.9620 1.000
H10 H 0.4267 0.4739 2.0297 1.000
H17A H -0.0879 0.5048 1.7966 1.000
H17B H -0.0604 0.4954 1.9265 1.000
H16A H 0.1974 0.6827 2.0275 1.000
H16B H 0.3195 0.7903 1.9564 1.000
H11A H 0.1723 0.3060 2.0255 1.000
H11B H 0.2946 0.1685 1.9644 1.000

[-x, y+1/2, -z]
Br1 BR -0.1385 0.4894 0.4932 1.000
C2 C -0.3480 0.4940 0.4717 1.000
C1 C -0.4330 0.4870 0.5560 1.000
C3 C -0.4359 0.4940 0.3797 1.000
O1 O -0.3535 0.4860 0.6459 1.000
C6 C -0.6050 0.4920 0.5216 1.000
H3 H -0.3815 0.5023 0.3162 1.000
C4 C -0.6050 0.4820 0.3665 1.000
H1 H -0.4085 0.5658 0.6873 1.000
C5 C -0.6740 0.4810 0.4397 1.000
C7 C -0.6949 0.4790 0.6174 1.000
Cl1 CL -0.7271 0.4930 0.2532 1.000
H5 H -0.8016 0.4698 0.4247 1.000
N1 N -0.6320 0.4860 0.6923 1.000
H7 H -0.8222 0.4631 0.6022 1.000
C8 C -0.7150 0.4900 0.7797 1.000
C14 C -0.8200 0.6630 0.7810 1.000
C13 C -0.8200 0.2930 0.7740 1.000

C9 C -0.5973 0.5040 0.8711 1.000
H14A H -0.9154 0.6576 0.7157 1.000
H14B H -0.7532 0.7958 0.7759 1.000
C15 C -0.8910 0.6750 0.8750 1.000
C12 C -0.8990 0.3090 0.8675 1.000
H13A H -0.9092 0.2874 0.7056 1.000
H13B H -0.7468 0.1628 0.7788 1.000
C10 C -0.6672 0.4780 0.9636 1.000
H9A H -0.5399 0.6460 0.8746 1.000
H9B H -0.5084 0.3920 0.8714 1.000
H15 H -0.9576 0.8098 0.8747 1.000
C17 C -1.0000 0.4980 0.8655 1.000
C16 C -0.7590 0.6680 0.9600 1.000
H12 H -0.9755 0.1830 0.8671 1.000
C11 C -0.7730 0.3030 0.9620 1.000
H10 H -0.5733 0.4739 1.0297 1.000
H17A H -1.0879 0.5048 0.7966 1.000
H17B H -1.0603 0.4954 0.9265 1.000
H16A H -0.8026 0.6827 1.0275 1.000
H16B H -0.6805 0.7903 0.9564 1.000
H11A H -0.8277 0.3060 1.0255 1.000
H11B H -0.7054 0.1685 0.9644 1.000

[x, y, z]
H5 H -0.1984 0.9698 0.5753 1.000
C5 C -0.3260 0.9810 0.5603 1.000
C4 C -0.3950 0.9820 0.6335 1.000
C6 C -0.3950 0.9920 0.4784 1.000
Cl1 CL -0.2729 0.9930 0.7468 1.000
C3 C -0.5641 0.9940 0.6203 1.000
C1 C -0.5670 0.9870 0.4440 1.000
C7 C -0.3051 0.9790 0.3826 1.000
H3 H -0.6185 1.0022 0.6838 1.000
C2 C -0.6520 0.9940 0.5283 1.000
O1 O -0.6465 0.9860 0.3541 1.000
N1 N -0.3680 0.9860 0.3077 1.000
H7 H -0.1778 0.9631 0.3978 1.000
Br1 BR -0.8615 0.9894 0.5068 1.000
H1 H -0.5915 1.0658 0.3127 1.000
C8 C -0.2850 0.9900 0.2203 1.000
C13 C -0.1800 0.7930 0.2260 1.000
C14 C -0.1800 1.1630 0.2190 1.000
C9 C -0.4027 1.0040 0.1289 1.000
H13A H -0.0908 0.7874 0.2944 1.000
C12 C -0.1010 0.8090 0.1325 1.000
H13B H -0.2532 0.6628 0.2212 1.000

C15 C -0.1090 1.1750 0.1250 1.000
H14A H -0.0846 1.1576 0.2843 1.000
H14B H -0.2468 1.2958 0.2241 1.000
C10 C -0.3328 0.9780 0.0364 1.000
H9A H -0.4601 1.1460 0.1254 1.000
H9B H -0.4916 0.8921 0.1286 1.000
H12 H -0.0245 0.6830 0.1329 1.000
C17 C 0.0000 0.9980 0.1345 1.000
C11 C -0.2270 0.8030 0.0380 1.000
H15 H -0.0424 1.3098 0.1253 1.000
C16 C -0.2410 1.1680 0.0400 1.000
H10 H -0.4267 0.9739 -0.0297 1.000
H17A H 0.0879 1.0049 0.2034 1.000
H17B H 0.0604 0.9954 0.0735 1.000
H11A H -0.1723 0.8060 -0.0255 1.000
H11B H -0.2946 0.6685 0.0356 1.000
H16A H -0.1974 1.1826 -0.0275 1.000
H16B H -0.3195 1.2903 0.0436 1.000

[x, y, z]
H5 H -0.1984 -0.0302 0.5753 1.000
C5 C -0.3260 -0.0190 0.5603 1.000
C4 C -0.3950 -0.0180 0.6335 1.000
C6 C -0.3950 -0.0080 0.4784 1.000
Cl1 CL -0.2729 -0.0070 0.7468 1.000
C3 C -0.5641 -0.0060 0.6203 1.000
C1 C -0.5670 -0.0130 0.4440 1.000
C7 C -0.3051 -0.0210 0.3826 1.000
H3 H -0.6185 0.0022 0.6838 1.000
C2 C -0.6520 -0.0060 0.5283 1.000
O1 O -0.6465 -0.0140 0.3541 1.000
N1 N -0.3680 -0.0140 0.3077 1.000
H7 H -0.1778 -0.0369 0.3978 1.000
Br1 BR -0.8615 -0.0106 0.5068 1.000
H1 H -0.5915 0.0658 0.3127 1.000
C8 C -0.2850 -0.0100 0.2203 1.000
C14 C -0.1800 0.1630 0.2190 1.000
C13 C -0.1800 -0.2070 0.2260 1.000
C9 C -0.4027 0.0040 0.1289 1.000
H14A H -0.0846 0.1576 0.2843 1.000
C15 C -0.1090 0.1750 0.1250 1.000
H14B H -0.2468 0.2958 0.2241 1.000
C12 C -0.1010 -0.1910 0.1325 1.000
H13A H -0.0908 -0.2126 0.2944 1.000
H13B H -0.2532 -0.3372 0.2212 1.000
C10 C -0.3328 -0.0220 0.0364 1.000

H9A H -0.4601 0.1460 0.1254 1.000
H9B H -0.4916 -0.1079 0.1286 1.000
H15 H -0.0424 0.3098 0.1253 1.000
C17 C 0.0000 -0.0020 0.1345 1.000
C16 C -0.2410 0.1680 0.0400 1.000
H12 H -0.0245 -0.3170 0.1329 1.000
C11 C -0.2270 -0.1970 0.0380 1.000
H10 H -0.4267 -0.0261 -0.0297 1.000
H17A H 0.0879 0.0049 0.2034 1.000
H17B H 0.0604 -0.0046 0.0735 1.000
H16A H -0.1974 0.1826 -0.0275 1.000
H16B H -0.3195 0.2903 0.0436 1.000
H11A H -0.1723 -0.1940 -0.0255 1.000
H11B H -0.2946 -0.3315 0.0356 1.000

[x, y, z]
H11A H -0.1723 -0.1940 0.9745 1.000
C11 C -0.2270 -0.1970 1.0380 1.000
C10 C -0.3328 -0.0220 1.0364 1.000
C12 C -0.1010 -0.1910 1.1325 1.000
H11B H -0.2946 -0.3315 1.0356 1.000
C16 C -0.2410 0.1680 1.0400 1.000
H10 H -0.4267 -0.0261 0.9703 1.000
C9 C -0.4027 0.0040 1.1289 1.000
C17 C 0.0000 -0.0020 1.1345 1.000
H12 H -0.0245 -0.3170 1.1329 1.000
C13 C -0.1800 -0.2070 1.2260 1.000
H16A H -0.1974 0.1826 0.9725 1.000
C15 C -0.1090 0.1750 1.1250 1.000
H16B H -0.3195 0.2903 1.0436 1.000
C8 C -0.2850 -0.0100 1.2203 1.000
H9A H -0.4601 0.1460 1.1254 1.000
H9B H -0.4916 -0.1079 1.1286 1.000
H17B H 0.0604 -0.0046 1.0735 1.000
H17A H 0.0879 0.0049 1.2034 1.000
H13A H -0.0908 -0.2126 1.2944 1.000
H13B H -0.2532 -0.3372 1.2212 1.000
H15 H -0.0424 0.3098 1.1253 1.000
C14 C -0.1800 0.1630 1.2190 1.000
N1 N -0.3680 -0.0140 1.3077 1.000
H14A H -0.0846 0.1576 1.2843 1.000
H14B H -0.2468 0.2958 1.2241 1.000
C7 C -0.3051 -0.0210 1.3826 1.000
C6 C -0.3950 -0.0080 1.4784 1.000
H7 H -0.1778 -0.0369 1.3978 1.000
C1 C -0.5670 -0.0130 1.4440 1.000

C5 C -0.3260 -0.0190 1.5603 1.000
O1 O -0.6465 -0.0140 1.3541 1.000
C2 C -0.6520 -0.0060 1.5283 1.000
H5 H -0.1984 -0.0302 1.5753 1.000
C4 C -0.3950 -0.0180 1.6335 1.000
H1 H -0.5915 0.0658 1.3127 1.000
Br1 BR -0.8615 -0.0106 1.5068 1.000
C3 C -0.5641 -0.0060 1.6203 1.000
Cl1 CL -0.2729 -0.0070 1.7468 1.000
H3 H -0.6185 0.0022 1.6838 1.000

[x, y, z]
Br1 BR 0.1385 -0.0106 0.5068 1.000
C2 C 0.3480 -0.0060 0.5283 1.000
C3 C 0.4359 -0.0060 0.6203 1.000
C1 C 0.4330 -0.0130 0.4440 1.000
C4 C 0.6050 -0.0180 0.6335 1.000
H3 H 0.3815 0.0022 0.6838 1.000
C6 C 0.6050 -0.0080 0.4784 1.000
O1 O 0.3535 -0.0140 0.3541 1.000
C5 C 0.6740 -0.0190 0.5603 1.000
Cl1 CL 0.7271 -0.0070 0.7468 1.000
C7 C 0.6949 -0.0210 0.3826 1.000
H1 H 0.4085 0.0658 0.3127 1.000
H5 H 0.8016 -0.0302 0.5753 1.000
N1 N 0.6320 -0.0140 0.3077 1.000
H7 H 0.8222 -0.0369 0.3978 1.000
C8 C 0.7150 -0.0100 0.2203 1.000
C14 C 0.8200 0.1630 0.2190 1.000
C9 C 0.5973 0.0040 0.1289 1.000
C13 C 0.8200 -0.2070 0.2260 1.000
H14A H 0.9154 0.1576 0.2843 1.000
H14B H 0.7532 0.2958 0.2241 1.000
C15 C 0.8910 0.1750 0.1250 1.000
H9A H 0.5399 0.1460 0.1254 1.000
C10 C 0.6672 -0.0220 0.0364 1.000
H9B H 0.5084 -0.1079 0.1286 1.000
C12 C 0.8990 -0.1910 0.1325 1.000
H13A H 0.9092 -0.2126 0.2944 1.000
H13B H 0.7468 -0.3372 0.2212 1.000
H15 H 0.9576 0.3098 0.1253 1.000
C17 C 1.0000 -0.0020 0.1345 1.000
C16 C 0.7590 0.1680 0.0400 1.000
C11 C 0.7730 -0.1970 0.0380 1.000
H10 H 0.5733 -0.0261 -0.0297 1.000
H12 H 0.9755 -0.3170 0.1329 1.000

H17A H 1.0879 0.0049 0.2034 1.000
H17B H 1.0603 -0.0046 0.0735 1.000
H16A H 0.8026 0.1826 -0.0275 1.000
H16B H 0.6805 0.2903 0.0436 1.000
H11A H 0.8277 -0.1940 -0.0255 1.000
H11B H 0.7054 -0.3315 0.0356 1.000

[-x, y+1/2, -z]
C14 C 0.1800 -0.3370 0.7810 1.000
H14A H 0.0846 -0.3424 0.7157 1.000
H14B H 0.2468 -0.2042 0.7759 1.000
C15 C 0.1090 -0.3250 0.8750 1.000
C8 C 0.2850 -0.5100 0.7797 1.000
H15 H 0.0424 -0.1902 0.8747 1.000
C16 C 0.2410 -0.3320 0.9600 1.000
C17 C 0.0000 -0.5020 0.8655 1.000
N1 N 0.3680 -0.5140 0.6923 1.000
C13 C 0.1800 -0.7070 0.7740 1.000
C9 C 0.4027 -0.4960 0.8711 1.000
H16B H 0.3195 -0.2097 0.9564 1.000
H16A H 0.1974 -0.3173 1.0275 1.000
C10 C 0.3328 -0.5220 0.9636 1.000
H17A H -0.0879 -0.4952 0.7966 1.000
H17B H -0.0604 -0.5046 0.9265 1.000
C12 C 0.1010 -0.6910 0.8675 1.000
C7 C 0.3051 -0.5210 0.6174 1.000
H13A H 0.0908 -0.7126 0.7056 1.000
H13B H 0.2532 -0.8372 0.7788 1.000
H9A H 0.4601 -0.3540 0.8746 1.000
H9B H 0.4916 -0.6079 0.8714 1.000
C11 C 0.2270 -0.6970 0.9620 1.000
H10 H 0.4267 -0.5261 1.0297 1.000
H12 H 0.0245 -0.8170 0.8671 1.000
C6 C 0.3950 -0.5080 0.5216 1.000
H7 H 0.1778 -0.5369 0.6022 1.000
H11A H 0.1723 -0.6940 1.0255 1.000
H11B H 0.2946 -0.8315 0.9644 1.000
C1 C 0.5670 -0.5130 0.5560 1.000
C5 C 0.3260 -0.5190 0.4397 1.000
O1 O 0.6465 -0.5140 0.6459 1.000
C2 C 0.6520 -0.5060 0.4717 1.000
H5 H 0.1984 -0.5302 0.4247 1.000
C4 C 0.3950 -0.5180 0.3665 1.000
H1 H 0.5915 -0.4342 0.6873 1.000
Br1 BR 0.8615 -0.5106 0.4932 1.000
C3 C 0.5641 -0.5060 0.3797 1.000

Cl1 CL 0.2729 -0.5070 0.2532 1.000
H3 H 0.6185 -0.4977 0.3162 1.000

[-x, y+1/2, -z]
H17A H -0.0879 0.5048 0.7966 1.000
C17 C 0.0000 0.4980 0.8655 1.000
H17B H -0.0604 0.4954 0.9265 1.000
C15 C 0.1090 0.6750 0.8750 1.000
C12 C 0.1010 0.3090 0.8675 1.000
C14 C 0.1800 0.6630 0.7810 1.000
H15 H 0.0424 0.8098 0.8747 1.000
C16 C 0.2410 0.6680 0.9600 1.000
H12 H 0.0245 0.1830 0.8671 1.000
C11 C 0.2270 0.3030 0.9620 1.000
C13 C 0.1800 0.2930 0.7740 1.000
C8 C 0.2850 0.4900 0.7797 1.000
H14A H 0.0846 0.6576 0.7157 1.000
H14B H 0.2468 0.7958 0.7759 1.000
H16B H 0.3195 0.7903 0.9564 1.000
C10 C 0.3328 0.4780 0.9636 1.000
H16A H 0.1974 0.6827 1.0275 1.000
H11B H 0.2946 0.1685 0.9644 1.000
H11A H 0.1723 0.3060 1.0255 1.000
H13A H 0.0908 0.2874 0.7056 1.000
H13B H 0.2532 0.1628 0.7788 1.000
N1 N 0.3680 0.4860 0.6923 1.000
C9 C 0.4027 0.5040 0.8711 1.000
H10 H 0.4267 0.4739 1.0297 1.000
C7 C 0.3051 0.4790 0.6174 1.000
H9A H 0.4601 0.6460 0.8746 1.000
H9B H 0.4916 0.3920 0.8714 1.000
C6 C 0.3950 0.4920 0.5216 1.000
H7 H 0.1778 0.4631 0.6022 1.000
C1 C 0.5670 0.4870 0.5560 1.000
C5 C 0.3260 0.4810 0.4397 1.000
O1 O 0.6465 0.4860 0.6459 1.000
C2 C 0.6520 0.4940 0.4717 1.000
H5 H 0.1984 0.4698 0.4247 1.000
C4 C 0.3950 0.4820 0.3665 1.000
H1 H 0.5915 0.5658 0.6873 1.000
Br1 BR 0.8615 0.4894 0.4932 1.000
C3 C 0.5641 0.4940 0.3797 1.000
Cl1 CL 0.2729 0.4930 0.2532 1.000
H3 H 0.6185 0.5023 0.3162 1.000

[x, y, z]

H11A H -0.1723 0.8060 0.9745 1.000
C11 C -0.2270 0.8030 1.0380 1.000
C12 C -0.1010 0.8090 1.1325 1.000
H11B H -0.2946 0.6685 1.0356 1.000
C10 C -0.3328 0.9780 1.0364 1.000
H12 H -0.0245 0.6830 1.1329 1.000
C17 C 0.0000 0.9980 1.1345 1.000
C13 C -0.1800 0.7930 1.2260 1.000
C16 C -0.2410 1.1680 1.0400 1.000
H10 H -0.4267 0.9739 0.9703 1.000
C9 C -0.4027 1.0040 1.1289 1.000
H17B H 0.0604 0.9954 1.0735 1.000
H17A H 0.0879 1.0049 1.2034 1.000
C15 C -0.1090 1.1750 1.1250 1.000
C8 C -0.2850 0.9900 1.2203 1.000
H13A H -0.0908 0.7874 1.2944 1.000
H13B H -0.2532 0.6628 1.2212 1.000
H16A H -0.1974 1.1826 0.9725 1.000
H16B H -0.3195 1.2903 1.0436 1.000
H9A H -0.4601 1.1460 1.1254 1.000
H9B H -0.4916 0.8921 1.1286 1.000
C14 C -0.1800 1.1630 1.2190 1.000
H15 H -0.0424 1.3098 1.1253 1.000
N1 N -0.3680 0.9860 1.3077 1.000
H14A H -0.0846 1.1576 1.2843 1.000
H14B H -0.2468 1.2958 1.2241 1.000
C7 C -0.3051 0.9790 1.3826 1.000
C6 C -0.3950 0.9920 1.4784 1.000
H7 H -0.1778 0.9631 1.3978 1.000
C1 C -0.5670 0.9870 1.4440 1.000
C5 C -0.3260 0.9810 1.5603 1.000
O1 O -0.6465 0.9860 1.3541 1.000
C2 C -0.6520 0.9940 1.5283 1.000
H5 H -0.1984 0.9698 1.5753 1.000
C4 C -0.3950 0.9820 1.6335 1.000
H1 H -0.5915 1.0658 1.3127 1.000
Br1 BR -0.8615 0.9894 1.5068 1.000
C3 C -0.5641 0.9940 1.6203 1.000
Cl1 CL -0.2729 0.9930 1.7468 1.000
H3 H -0.6185 1.0022 1.6838 1.000
